# Global trends, decomposition analysis, inequality assessment, and economic projections of tracheal, bronchus, and lung cancer

**DOI:** 10.3389/fpubh.2025.1745506

**Published:** 2026-01-26

**Authors:** Wenxuan Li, Yue Cong, Xinyu Liu, Zhangyan Lyu, Kexin Chen

**Affiliations:** Department of Epidemiology and Biostatistics, Key Laboratory of Prevention and Control of Human Major Diseases of the Ministry of Education, Key Laboratory of Molecular Cancer Epidemiology of Tianjin, National Clinical Research Center for Cancer, Tianjin's Clinical Research Center For Cancer, Tianjin Medical University Cancer Institute and Hospital, Tianjin Medical University, Tianjin, China.

**Keywords:** decomposed analysis, economic burden, global disease burden, health inequality, tracheal, bronchus, lung cancer

## Abstract

**Background:**

This study analyzed global trends in incidence, mortality, disability-adjusted life years (DALYs), and economic burden of tracheal, bronchus, and lung (TBL) cancer from 1990 to 2021, focusing on regional and sex differences, with projections to 2050.

**Methods:**

Using joinpoint regression to assess temporal trends. Decomposition analysis quantified the effects of population growth, aging, and changes in disease fatality. Health inequalities were evaluated using the concentration index (CI), and the economic burden was estimated through a value of statistical life (VSL).

**Results:**

Globally, TBL cancer deaths increased from 1.08 million in 1990 to 2.02 million in 2021, and DALYs rose from 28.46 million to 46.54 million, with a sharper rise among females. Population growth (94.18%) and population aging (36.07%) were the major components to the observed increase in the global TBL cancer burden. East Asia accounted for over half of the global increase, with China contributing the largest national share. The global economic burden is projected to rise from $3.86 trillion in 2021 to $7.15 trillion by 2050. China's economic loss from TBL cancer is estimated to increase from $688 billion in 2021 to $2.49 trillion by 2050, surpassing the United States and reflecting the rapid escalation of burden in Asia.

**Conclusion:**

TBL cancer remains a major global health challenge, requiring urgent, region-specific action to reduce its growing impact.

## Background

Tracheal, bronchus, and lung (TBL) cancer is the leading cause of cancer-related mortality, accounting for approximately 1.8 million deaths worldwide in 2022 (1). Despite advances in prevention and treatment, the 5-year survival rate for patients with TBL cancer is still relatively poor, ranging between 10% and 20% in most countries (2). Although COVID-19 infection itself is not established as a direct risk factor for lung cancer, the COVID-19 pandemic had substantial indirect effects on cancer epidemiology through widespread disruptions to screening, diagnostic pathways, and timely access to treatment (3, 4). These system-level delays and interruptions have been shown to influence the detection and outcomes of multiple cancers, including those of the respiratory system.

Previous studies have explored global trends in the TBL disease burden, as well as examining specific countries or regions and investigating modifiable risk factors such as smoking, ambient pollution, and high fasting plasma glucose etc. (5–7). These findings highlighted regional and sex-based heterogeneity, likely influenced by variations in risk factor distribution. However, although previous studies have described disparities in TBL cancer burden, few have examined demographic factors—such as population size, age structure, and disease severity—specifically contribute to the observed variations. Furthermore, various literature indicates that TBL cancer is prohibitively costly regarding direct medical expenses and indirect costs (8, 9). Evaluation of the economic burden of TBL cancer is essential for building investment cases for universal cancer control and informing public health decision-making. The United Nations Sustainable Development Goals (SDGs) emphasize the need to address health disparities, promote gender equality, and reduce inequalities within and among countries (10). However, there is still limited research analyzing TBL cancer burden through the lens of these global goals.

To address existing knowledge gaps, this study characterizes the observed changes in the TBL cancer burden using a decomposition analysis and quantified cross-country inequalities in disease outcomes. We further estimate the temporal and spatial patterns of the economic burden of TBL cancer through 2050 using a value of statistical life (VSL) framework. Building on these objectives, this research provides an integrative quantitative assessment of how population aging indicators, health status, and regional variation collectively shape the global TBL cancer burden. By simultaneously examining the economic consequences across diverse geographic and demographic contexts, the study offers insights that have been largely overlooked in prior work. Through identifying and quantifying the substantial financial losses attributable to TBL cancer, our findings underscore the need for region-specific and data-driven prevention and control strategies informed by the most current evidence.

## Method

### Data source

We obtained the GBD 2021 datasets from the Global Health Data Exchange (GHDx) query tool (https://vizhub.healthdata.org/gbd-results/). According to SDI, GBD 2021 divides the world into five regions (11). The study used the International Classification of Diseases Tenth Revision (ICD-10) diagnosis codes to classify TBL cancer cases. Following the Global Burden of Disease cause-mapping framework, incidence was defined using ICD-10 codes C33, C34–C34.92, Z12.2, Z80.1–Z80.2, and Z85.1–Z85.20, whereas mortality was identified using codes C33–C34.9, D02.1–D02.3, D14.2–D14.3, and D38.1 (12). TBL cancer-specific incidence, prevalence, deaths, DALYs, and corresponding population data from 1990 to 2021 at the global and regional levels were also obtained (13). Age-standardized death rate (ASDR) and age-standardized disability-adjusted life year rate (ASDAR) were computed based on the GBD standard population.


$$\begin{array}{l} ASDR=\overset{n}{\underset{agei}{\sum }}{\left(\frac{{DALY\;number}_{agei}}{population_{agei}}\right)}^{*}{standard\text{\_}popuation}_{agei} \end{array}$$

where agei represent the i^−*th*^ age range with a span of 5 years old. Similar calculation methods were used to calculate ASDAR (14).

All estimates contain 95% uncertainty intervals (UIs) for each metric, which were calculated in the posterior simulation of 500 drawings to estimate uncertain distributions from random and systematic errors (15).

The University of Washington Institutional Review Board has approved a waiver of informed consent for the use of deidentified data in the GBD study.

### Decomposed analysis

TBL cancer is categorized as an “age-related disease” in the GBD study. This categorization stems from the observation that the incidence of TBL cancer in adults (aged over 25 years) increases exponentially with age (11). To analyze the changing factors behind the changes in age-related TBL cancer burden, we adopted the decomposition method developed by Gupta (16). Further details are specified in the Supplementary Section 1.

### Measures of health inequality

To assess the health disparity associated with TBL cancer across socio-economic strata, the concentration index (CI) and concentration curves were employed, following World Health Organization (WHO) recommendation (17). The covariance approach was used to compute the CI (18).


$$\begin{array}{l} CI=\frac{2}{\mu }cov\left(h_{i},r_{i}\right) \end{array}$$

where *cov* represents covariance, *r* means the rank of region *i* in socio-economic distribution (from the poorest to the richest), *h* represents health outcome and μ is considered the mean health outcome. The CI ranged between −1 and 1, with negative values indicating a higher concentration of disease burden in countries with lower socio-economic development and vice versa (14). The Bootstrap method was applied to compute the 95% CI. We used a non-parametric percentile bootstrap with 1,000 resamples. The 2.5th and 97.5th percentiles of the bootstrap distribution were taken as the bounds of the 95% CI. The average annual percentage change (AAPC) of CI to illustrate the temporal trend of health inequality between 1990 and 2021. The Joinpoint Regression Program (version 5.0) automatically detects these joinpoints by analyzing the data for the best-fitting points with statistically significant shifts (19). In this study, we allowed a maximum of five joinpoints, and model selection was based on the Monte Carlo permutation test. The final model was chosen using permutation test results and the Bayesian Information Criterion (BIC) to ensure optimal fit and parsimony. These joinpoints define periods for computing the Annual Percentage Change (APC) and the Average Annual Percentage Change (AAPC) and corresponding 95% Confidence Intervals (CIs) (20).

### Economic burden

We projected the global, regional, and national economic burdens of TBL cancer by 2050 using the VSL approach. This method, based on willingness-to-pay philosophy, is extensively utilized in health economics research (21, 22). In this study, we first calculated country-specific VSL values and then derived the value of a statistical life year (VSLY) based on VSL and life expectancy. Finally, the economic burden was estimated based on VSLY values and disease burden. The 95% uncertainty intervals for economic burden estimates were generated using the uncertainty intervals of GBD DALYs. To account for uncertainty in income–VSL relationships, we evaluated two additional scenarios in which income elasticities of E = 1 and E = 1.5 were assumed for all countries. Further details of the economic burden analysis methodology are provided in the Supplementary Section 2.

## Result

### Disease burden of TBL cancer between 1990 and 2021

Compared to 1990 (deaths: 1.08 million; DALYs: 28.46 million), the number of TBL cancer-related deaths (2.02 million) and DALYs (46.54 million) in 2021 increased by 0.87 and 0.64, respectively (Table 1, Supplementary Table 1). Regions with middle and low-middle SDI saw the largest increases in both deaths and DALYs, with increases of 1.72 and 1.50, respectively. Between 1990 and 2021, both cancer-related deaths and DALYs from TBL increased more among females (1.40 and 1.10, respectively) than males (0.68 and 0.48, respectively). Additionally, the rise in TBL cancer-related deaths and DALYs was consistently greater among females than males across all SDI regions (Table 1, Supplementary Tables 2–5).

**Table 1:** The DALYs number and percentage change for tracheal, bronchus, and lung cancer between 1990 and 2021.

**Location**	**DALYs number (in 1,000) 95%UI**		**Percentage change**
	**1990**	**2021**	
Global	28,459.84 (26,973.71, 29,909.11)	46,536.27 (41,903.41, 51,205.05)	0.64 (0.44, 0.84)
**Socio-demographic index region**			
High SDI	10,397.78 (10,111.51, 10,587.65)	12,049.99 (11,255.22, 12,566.24)	0.16 (0.11, 0.20)
High-middle SDI	9,891.90 (9,240.77, 10,518.60)	15,192.41 (13,294.99, 17,333.21)	0.54 (0.31, 0.78)
Middle SDI	6,275.48 (5,632.24, 6,951.18)	14,737.80 (12,360.03, 17,016.25)	1.35 (0.89, 1.84)
Low-middle SDI	1,472.28 (1,299.25, 1,710.94)	3,686.13 (3,342.29, 4,067.40)	1.50 (1.05, 1.93)
Low SDI	383.61 (321.17, 489.63)	820.69 (691.13, 974.35)	1.14 (0.79, 1.66)
Region			
**Southeast Asia, East Asia, and Oceania**			
East Asia	7,990.80 (6,825.88, 9,207.73)	19,392.78 (15,619.68, 23,515.09)	1.43 (0.84, 2.18)
Southeast Asia	1,387.85 (1,230.91, 1,548.88)	3,556.36 (2,921.60, 4,139.20)	1.56 (0.99, 2.01)
Oceania	13.67 (9.93, 20.51)	36.02 (27.03, 50.97)	1.64 (0.98, 2.51)
**Central Europe, Eastern Europe, and Central Asia**			
Central Asia	402.57 (381.89, 424.96)	307.27 (271.52, 341.69)	−0.24 (−0.33, −0.14)
Central Europe	1,649.47 (1,596.57, 1,703.16)	1,890.39 (1,751.62, 2,013.63)	0.15 (0.06, 0.24)
Eastern Europe	2,914.02 (2,848.35, 2,973.45)	1,903.31 (1,721.44, 2,076.53)	−0.35 (−0.41, −0.28)
**High-income**			
High-income Asia Pacific	1,185.51 (1,143.73, 1224.42)	1,959.36 (1,743.09, 2094.91)	0.65 (0.51, 0.76)
Australasia	186.99 (178.27, 195.65)	251.30 (229.4, 273.02)	0.34 (0.23, 0.47)
Western Europe	4,927.98 (4,795.22, 5,031.74)	5,138.16 (4,809.13, 5375.90)	0.04 (-0.01, 0.09)
Southern Latin America	355.15 (334.40, 378.10)	399.69 (367.21, 436.55)	0.13 (0.01, 0.25)
High-income North America	4,163.64 (4,020.96, 4,267.08)	4,077.29 (3,822.85, 4,251.01)	−0.02 (-0.06, 0.01)
**Latin America and Caribbean**			
Caribbean	140.62 (133.16, 150.08)	250.03 (220.66, 282.64)	0.78 (0.56, 1.03)
Andean Latin America	72.49 (63.14, 83.30)	149.31 (114.99, 185.52)	1.06 (0.54, 1.61)
Central Latin America	310.65 (302.12, 318.39)	580.68 (510.22, 661.89)	0.87 (0.65, 1.13)
Tropical Latin America	405.78 (392.60, 418.45)	937.92 (885.78, 985.95)	1.31 (1.20, 1.43)
**North Africa and Middle East**			
North Africa and Middle East	887.77 (738.76, 1,054.11)	1,963 (1,702.4, 2,256.01)	1.21 (0.77, 1.86)
**South Asia**			
South Asia	1,030.09 (897.74, 1,187.81)	2,794.3 (2,359.35, 3,179.66)	1.71 (1.05, 2.33)
**Sub-Saharan Africa**			
Central Sub-Saharan Africa	65.26 (47.15, 97.50)	149.97 (104.68, 227.36)	1.3 (0.64, 2.30)
Southern Sub-Saharan Africa	134.73 (116.6, 161.18)	300.68 (270.78, 336.98)	1.23 (0.89, 1.67)
Eastern Sub-Saharan Africa	154.25 (129.51, 192.96)	292.38 (250.53, 353.7)	0.9 (0.47, 1.44)
Western Sub-Saharan Africa	80.55 (67.33, 95.75)	206.1 (167.39, 250.33)	1.56 (1.07, 2.22)

DALYs, disability-adjusted life-years; SDI, Socio-demographic index; UIs, uncertainty intervals.

At the GBD regional level, East Asia experienced the largest increase in TBL-related deaths, rising from 0.29 million in 1990 to 0.83 million in 2021. Meanwhile, South Asia saw the greatest increase in TBL-related DALYs, from 1.03 million in 1990 to 2.79 million in 2021. In contrast, both TBL-related deaths and DALYs in Central Asia and Eastern Europe exhibited a downward trend between 1990 and 2021. Additionally, the gender disparity in TBL cancer burden trends remained consistent across all regions, with a more pronounced decrease observed in males (Table 1, Supplementary Tables 2–5).

### Decomposed analysis on TBL cancer deaths and DALYs

**Figure 1:**
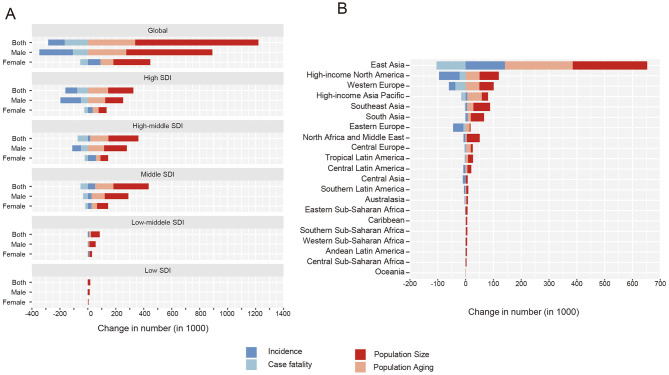
**(A)** Decomposition of change in age-related deaths for tracheal, bronchus, and lung cancer in 5 SDI regions between 1990 and 2021. **(B)** Decomposition of change in age-related deaths for tracheal, bronchus, and lung cancer in 21 GBD regions between 1990 and 2021.

**Figure 2:**
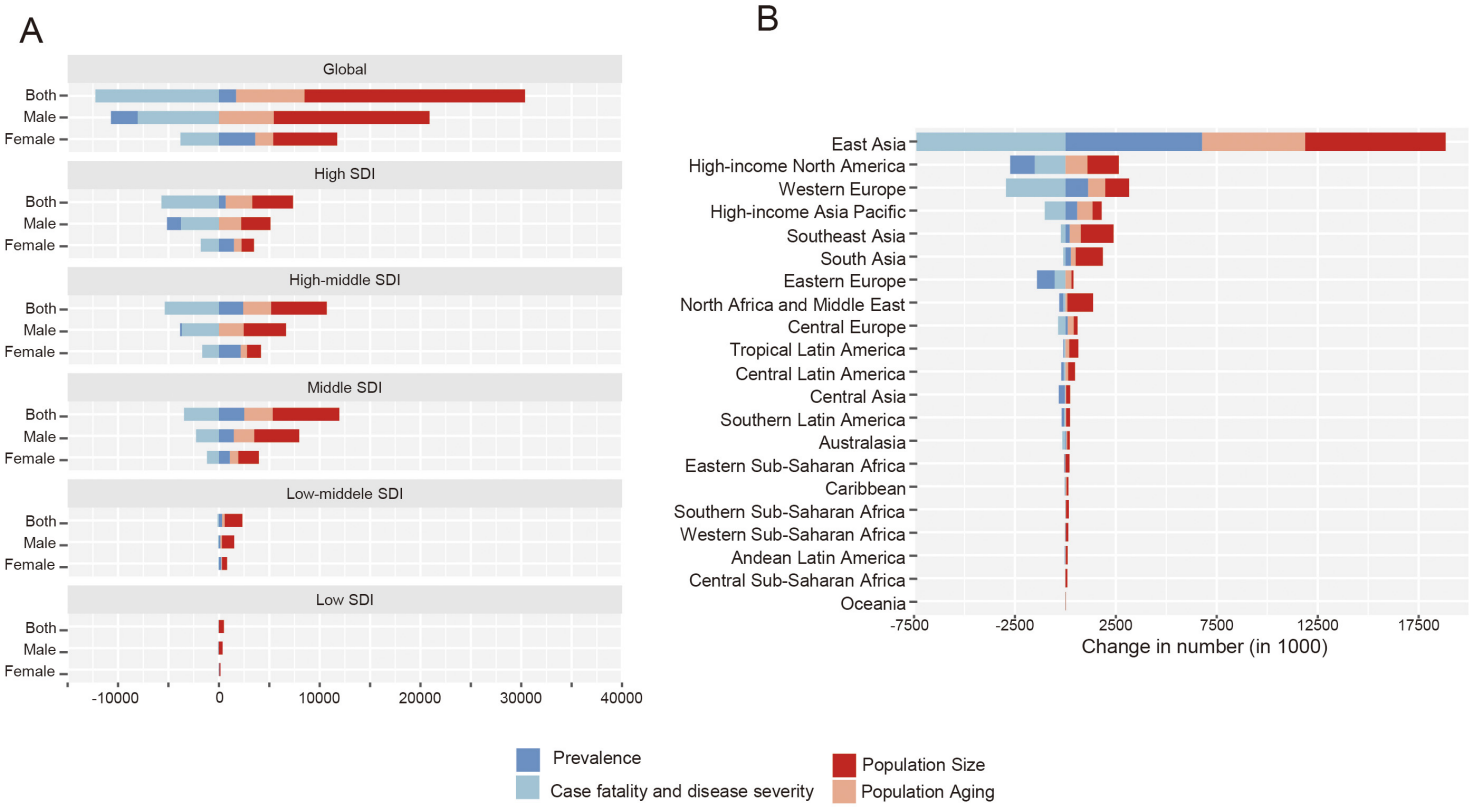
**(A)** Decomposition of change in age-related DALYs for tracheal, bronchus, and lung cancer in 5 SDI regions between 1990 and 2021. **(B)** Decomposition of change in age-related DALYs for tracheal, bronchus, and lung cancer in 21 GBD regions between 1990 and 2021. DALYs, disability-adjusted life-years; SDI, Socio-demographic index.

Within the decomposition framework, changes in adult population size constituted the largest numerical component of the observed increase in TBL cancer deaths, corresponding to 0.88 million deaths (94.18% of the total decomposition components) and 21.88 million DALYs (120.73%). Population aging followed, accounting for 0.34 million deaths (36.07%) and 6.79 million DALYs (37.47%). Concomitantly, reductions in disease fatality and severity were reflected as negative numerical components in the decomposition analysis, representing 164.80 thousand deaths (17.59%) and 12.24 million DALYs (67.59%), respectively. Notably, some decomposition components exceeded ±100%, which reflects the mathematical structure of the method and occurs when the numerical change attributed to a component outweighs or moves in the opposite direction of the net observed change, rather than indicating an epidemiologic magnitude (Figures 1, 2, Supplementary Tables 6, 7). Sex-stratified analyses showed that the decomposition assigned larger negative components to fatality and severity among males than females, both for deaths (fatality: −19.59% vs. −13.68%) and for DALYs (fatality and severity: −78.98% vs. −48.03%) (Supplementary Tables 8–11).

The proportion of population aging to the elevated TBL cancer-related deaths (high: 87.99%, high-middle: 45.13%, middle: 34.30%, low-middle: 14.09%, low: −7.44%) and DALYs (high: 159.22%, high-middle: 52.07%, middle: 33.20%, low-middle: 11.18%, low: −7.57%) increased with SDI levels. Similarly, the effect of optimized fatality and severity on lowered TBL cancer-related deaths (low-middle: −1.72%, low: −1.02%) and DALYs (low-middle: −5.83%, low: −4.83%) were most faint at lower SDI countries (Figures 1, 2, Supplementary Tables 6, 7) Across GBD regions, East Asia accounted for more than half of the global variation in TBL cancer deaths (58.53%) and DALYs (63.23%) between 1990 and 2021. Additionally, the proportion of fatality and severity on lowered TBL cancer-related burden was smaller than the global level (death: −17.59%, DALYs: −67.59%) in almost all regions except for high-income areas (Supplementary Tables 8–11).

### Health inequalities in TBL cancer burden

**Figure 3:**
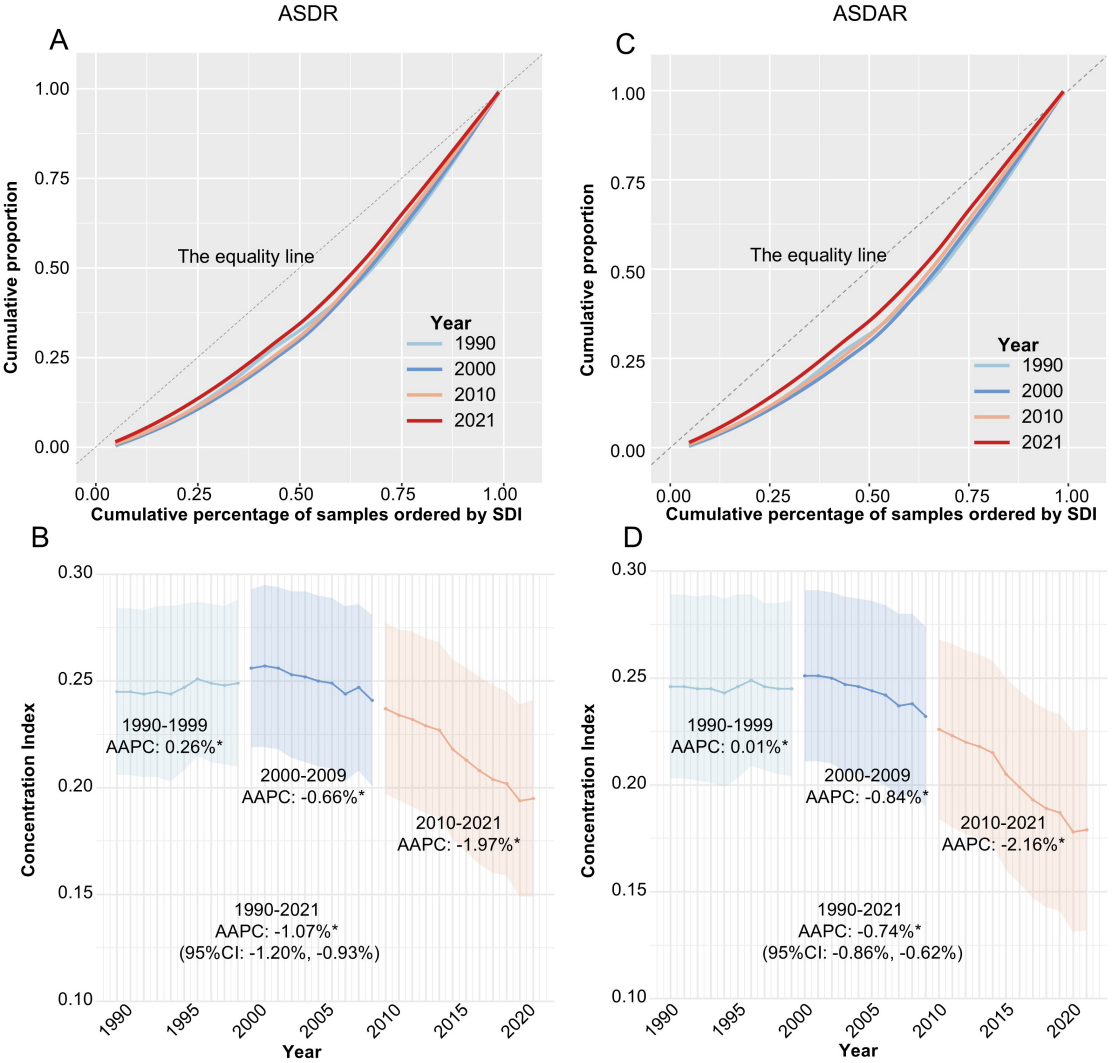
Concentration curves and AAPC of concentration indices of tracheal, bronchus, and lung cancer across 21 GBD region between 1990 and 2021. **(A)** Concentration curves of ASDR; **(B)** Concentration indices of ASDR; **(C)** Concentration curves of ASADR; **(D)** Concentration indices of ASADR. ASDR, Age- standardized rate of Deaths; ASDAR, Age-standardized rate of DALYs; DALYs, disability-adjusted life-years; SDI, Socio-demographic index; AAPC, average annual percentage change.

Health inequality analysis found a positive CI for TBL cancer in ASDR (0.20 ± 0.05) and ASDAR (0.18 ± 0.05) in 2021. The TBL cancer burden remains unequally distributed in recent years, with higher SDI regions bearing a disproportionately high burden (Figures 3A, B).

Further analysis of the temporal trends of the CI revealed that TBL cancer-related health inequality has gradually decreased from 1990 to 2021, with statistically significant AAPC for CI in ASDR (−1.07%, 95% CIs: −1.20%, −0.93%) and ASDAR (−0.74%, 95% CIs: −0.86%, −0.62%). This trend was consistent across both sexes (CI of ASDR: males, −0.96, 95%CIs: −1.13, −0.78; females, −0.30, 95%CIs: −0.58, −0.03) (Supplementary Table 12). Interestingly, we noticed that, compared to the period 1990–2009, the magnitude of the decline in CI in the last decade (2010–2021) has become greater, with AAPCs of −1.97% (95%CIs: −2.20%, −1.74%) for ASDR and −2.16% (95%CIs: −3.00%, −1.32%) for ASDAR (Figures 3C, D).

### Projected economic burden of TBL cancer (2021–2050)

If the rates remain the same as those in 2021, it is projected that approximately 3,400,000 TBL cancer-related deaths and 64,000,000 TBL cancer-related DALYs will occur by 2050, indicating increases of 70.81% and 38.08%, respectively. The estimated global economic burden of TBL cancer is anticipated to rise from $3.86 trillion (95%UIs: 3.37, 4.32) in 2021, to $4.88 trillion (95%UIs: 4.07, 5.70) by 2030, $5.95 trillion (95%UIs: 4.75, 7.20) by 2040, and $7.15 trillion (95%UIs: 5.35, 9.17) yielding an 85.23% increase. In the future, as the disease burden evolves differently between male and female populations, the gap in economic burden between sexes is projected to gradually narrow (Table 2).

**Table 2:** Estimated economic burden of tracheal, bronchus, and lung cancer by SDI regions through 2050, billions of 2023 US$.

**Region**	**2021 economic burden 95%UI**	**2030 economic burden 95%UI**	**2040 economic burden 95%UI**	**2050 economic burden 95%UI**
**World**				
Both	3,857.46 (3,373.57, 4,315.36)	4,880.10 (4,066.89, 5,698.57)	5,954.91 (4,750.44, 7,200.07)	7,145.44 (5,347.33, 9,166.25)
Male	2,485.45 (2,141.2, 2,841.03)	3,048.52 (2,490.52, 3,628.24)	3,595.9 (2,819.70, 4,431.27)	4,155.34 (3,068.10, 5,381.16)
Female	1,372.01 (1,168.77, 1,555.12)	1,831.58 (1,490.26, 2,181.85)	2,359 (1,830.36, 2,940.28)	2,990.11 (2,170.94, 3,954.50)
**Socio-demographic index region**				
**High SDI**				
Both	2,278.10 (2,087.59, 2,432.84)	2,662.15 (2,307.84, 2,997.31)	2,887.00 (2,430.19, 3,355.51)	3,058.79 (2,478.44, 3,694.99)
Male	1,382.73 (1,276.31, 1,480.75)	1,554.09 (1,350.26, 1,768.46)	1,613.72 (1,363.02, 1,899.15)	1,634.42 (1,320.45, 1,997.57)
Female	895.37 (799.44, 964.59)	1,108.06 (943.03, 1,258.45)	1,273.29 (1,045.72, 1,502.49)	1,424.37 (1,118.91, 1,755.82)
**High-middle SDI**				
Both	1,219.46 (1,025.05, 1,425.01)	1,713.62 (1,399.36, 2,052.92)	2,399.44 (1,866.92, 2,961.28)	3,241.4 (2,323.91, 4,296.41)
Male	860.09 (690.66, 1,043.71)	1,164.13 (901.51, 1,430.06)	1,558.53 (1,163.44, 1,965.24)	2,003.52 (1,404.82, 2,664.90)
Female	359.37 (292.74, 431.27)	549.5 (435.28, 681.00)	840.9 (635.10, 1,090.51)	1,237.88 (864.28, 1,716.23)
**Middle SDI**				
Both	331.64 (238.64, 422.83)	461.9 (327.34, 594.04)	605.77 (408.79, 799.09)	755.25 (486.65, 1,043.37)
Male	222.4 (158.73, 290.96)	301.23 (217.18, 392.04)	382.76 (264.87, 510.83)	461.59 (307.46, 636.02)
Female	109.24 (70.32, 149.05)	160.67 (102.05, 224.77)	223.02 (134.49, 317.01)	293.66 (166.03, 431.46)
**Low-middle SDI**				
Both	27.34 (21.62, 33.38)	41.02 (31.37, 52.32)	60.31 (42.91, 80.75)	85.57 (55.42, 124.9)
Male	19.58 (15.06, 24.65)	28.1 (20.91, 36.28)	39.29 (27.31, 53.65)	52.84 (33.49, 78.15)
Female	7.76 (6.10, 9.82)	12.92 (9.62, 16.98)	21.02 (14.56, 29.09)	32.73 (20.81, 48.71)
**Low SDI**				
Both	0.93 (0.66, 1.30)	1.4 (0.97, 1.99)	2.38 (1.62, 3.43)	4.43 (2.9, 6.58)
Male	0.66 (0.45, 0.95)	0.97 (0.65, 1.42)	1.6 (1.06, 2.39)	2.96 (1.88, 4.53)
Female	0.27 (0.18, 0.39)	0.43 (0.28, 0.64)	0.77 (0.49, 1.17)	1.48 (0.91, 2.28)

SDI, Socio-demographic index. Data were available for 169 countries covering more than 99% of the world population. Estimates were adjusted to 2023 constant US$ using consumer price index data from the U.S. Bureau of Labor Statistics. All future year estimates were discounted at a 3% annual discount rate.

Between 2021 and 2050, the areas with the highest economic burden of TBL cancer will shift from high SDI region to high-middle SDI regions. Specifically, in 2021, the TBL cancer economic burden in the high SDI region was the heaviest at $2.27 trillion (95%UIs: 2.09, 2.43), which is 1.87, 6.87, 83.33, and 2,451.98 times that in high-middle ($1.22 trillion, 95%UIs: 1.03, 1.43), middle ($0.33 trillion, 95%UIs: 0.24, 0.42), low-middle ($0.03 trillion, 95%UIs: 0.02, 0.03) and low SDI region ($0.93 billion, 95%UIs: 0.33, 1.30), respectively. By 2050, the pivotal change is anticipated, with the high-middle SDI region bearing the heaviest economic burden globally due to TBL cancer ($3.24 trillion, 95%UIs: 2.32, 4.30) (Table 2). These projected patterns remained consistent when alternative income elasticity assumptions (E = 1.0 and E = 1.5) were applied, indicating that the overall regional ranking of future economic burden was robust across different VSL scenarios (Supplementary Tables 13, 14).

**Figure 4:**
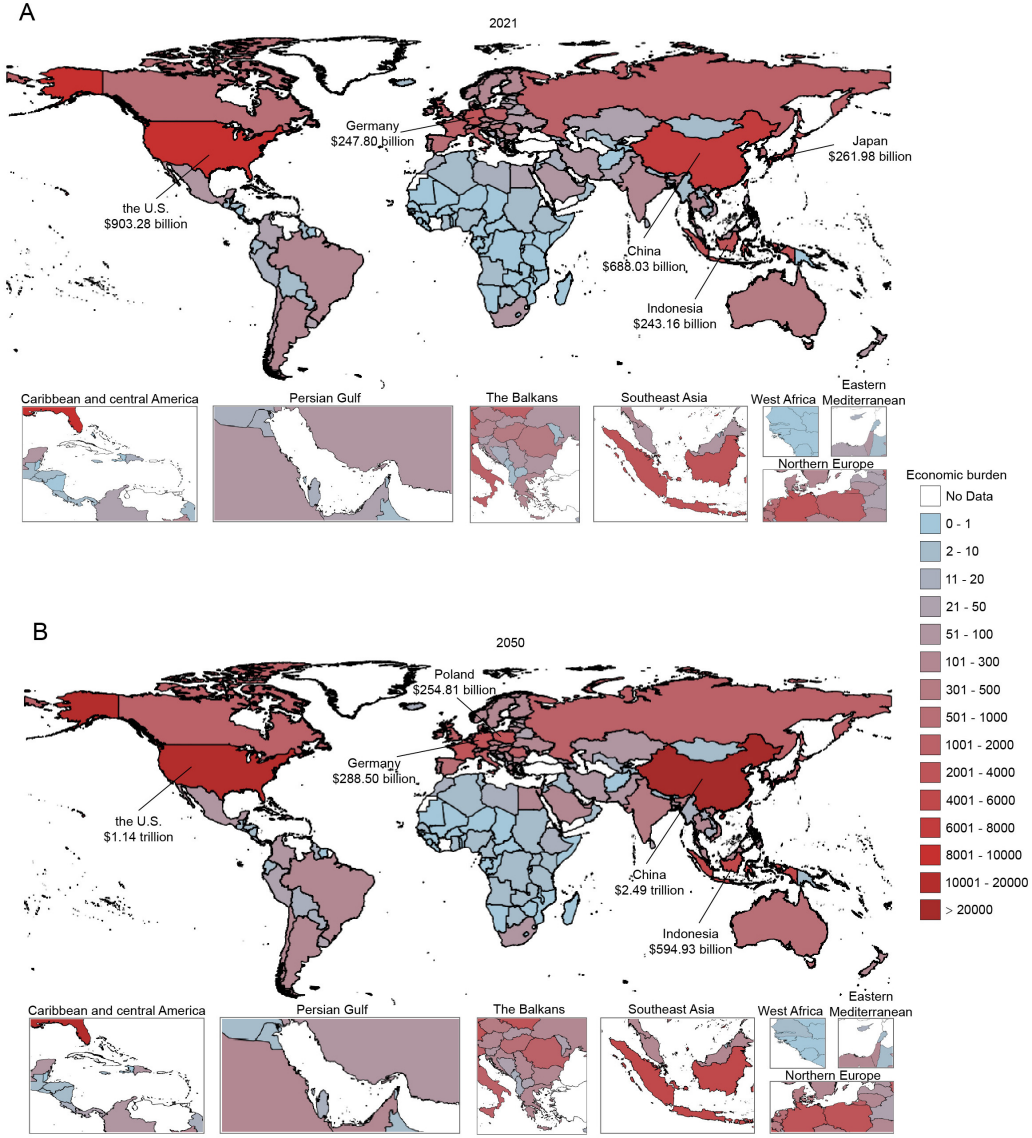
Estimated economic burden of tracheal, bronchus, and lung cancer by country through 2050, 100 millions of 2023 US$. **(A)** Estimated economic burden of tracheal, bronchus, and lung cancer by country in 2021. **(B)** Estimated economic burden of tracheal, bronchus, and lung cancer by country in 2050. Data were available for 169 countries covering more than 99% of the world population. Estimates were adjusted to 2023 constant US$ using consumer price index data from the U.S. Bureau of Labor Statistics. All future year estimates were discounted at a 3% annual discount rate. Countries without data are marked with white color.

In terms of national-level economic burden, the five countries with the most considerable economic burden due to TBL cancer were the US ($903.28 billion, 95%UIs: 844.86, 942.83), China ($688.03 billion, 95%UIs: 549.13, 840.44), Japan ($261.98 billion, 95%UIs: 233.71, 277.26), Germany ($247.80 billion, 95%UIs: 227.66, 267.70), and Indonesia ($243.16 billion, 95%UIs: 164.99, 318.58) in 2021. By 2050, the country with the highest TBL cancer economic burden globally will be China with $2.49 trillion (95%UIs: 1.76, 3.32), followed by the US with $1.14 trillion (95%UIs: 0.95, 1.35), Indonesia with $594.93 billion (95%UIs: 376.23, 821.99), Germany with $288.50 billion (95%UIs: 238.90, 338.14), and Poland with $254.81 billion (95%UIs: 198.02, 318.50) (Figure 4, Supplementary Tables 15–20).

## Discussion

In this study, we provided the first comprehensive quantification of global and regional trends in the disease and economic burden of TBL cancer, including an analysis of the change factors behind the growth in disease burden and the changing patterns of economic burden. Importantly, the decomposition results showed that the component representing adult population growth constituted the largest share of the observed changes in the global TBL cancer burden, exceeding the share attributed to population aging. Meanwhile, reductions in fatality and severity appeared as larger negative components in higher-SDI regions and among males. We also observed a measurable decline in health burden inequality over the past decade. Additionally, we anticipated a shift in the areas with the highest economic liabilities from high to high-middle SDI regions. Over time, the economic burdens of TBL cancer for males and females are expected to converge. Overall, by combining trend evaluation, decomposition analyses, inequality assessments, and economic projections, our study delivers a holistic and structurally informed assessment of how the global TBL cancer burden is changing. These innovative insights not only deepen our understanding of the dynamics surrounding TBL cancer but also offer essential guidance for prioritizing regions, populations, and strategies for effective prevention and treatment.

Females exhibited a substantial increase in the disease and economic burden associated with TBL cancer in almost all areas compared to males. The burden of smoking-associated TBL cancer has shown a downward trend, thus reflecting a global decline in smoking prevalence. Conversely, the burden of TBL cancer in never-smokers is on the rise (23), it is more frequently diagnosed in women and is preferentially prevalent among individuals of Asian descent. In East Asia, the higher burden among women has been consistently linked to a distinct molecular and etiological profile, characterized by a high prevalence of oncogenic driver mutations such as *EGFR* mutations and *ALK* rearrangements (23, 24), which occur more frequently in East Asia female than in other populations. These molecular features contribute to both disease susceptibility and clinical presentation and partly explain the elevated burden observed in East Asian women. Recent researches have increasingly highlighted the role of sex hormones, particularly estrogens, in the pathogenesis of lung cancer. Estrogen receptors are expressed in lung tissues, and their activation can influence crucial cellular processes, including proliferation, apoptosis, and angiogenesis, thereby contributing to tumor development (25). Moreover, females might be more exposed to certain environmental and occupational risk factors for TBL cancer, including indoor radon (26), secondhand smoke, and carcinogens related to cooking and heating practices than males, especially in low- and middle-income countries (27). Finally, our first analysis of TBL cancer showed that fatality and severity impact females less than males, suggesting that early detection and survival efforts may have somewhat overlooked women.

Existing studies have shown that the global burden of TBL cancer, as reflected in ASDR and ASDAR, has declined over recent decades (5). This trend is primarily driven by the success of smoking reduction campaigns (28) and advances in early detection and targeted therapy (29), particularly the global decline in smoking rates and the widespread adoption of low-dose CT screening, which have facilitated earlier diagnosis and significantly reduced mortality (30, 31). Complementing these risk-factor based findings, our decomposition analysis illustrates how demographic and disease-severity components have jointly shaped the observed increases in absolute deaths and DALYs. The decomposition results showed that, after the component representing adult population growth, the aging component accounted for the next largest share of the observed increases in deaths and DALYs. This trend was particularly pronounced in high SDI regions. World Health Organization (WHO) has championed the concept of “healthy aging,” a pivotal initiative to enhance the quality of life for older individuals (32). The statistic is that only about 10% of individuals over 65 in high-SDI countries like the United States and Europe are achieving healthy aging (33). To address these challenges, strengthening interventions that promote healthy lifestyles, mitigate cancer risk factors, and enhance the wellbeing of older population is essential. These measures not only reduce the disease burden but also contribute significantly to the realization of SDGs, which emphasizes ensuring healthy lives and promoting wellbeing for all at all ages. Therefore, these regions necessitate targeted prevention and control strategies specifically for the older population. Implementing measures that promote healthy lifestyles, mitigate cancer risk factors, and enhance the overall wellbeing of the older adults is crucial to alleviating the burden of TBL cancer.

Another significant finding from our inequality analysis is that regional differences in TBL cancer burden have decreased. This reduction was partly due to opposing trends in disease burden between higher and lower SDI areas and partly caused by an increase in population size in middle and low SDI areas, as revealed by the decomposition analysis. The mitigating effect of reduced TBL cancer fatality and severity was weaker in regions with middle and low SDI levels, whereas it significantly affected TBL cancer-related deaths and DALY numbers in high and high-middle SDI regions. This phenomenon may be attributed to higher SDI areas that often have more comprehensive healthcare coverage and insurance schemes (34), ensuring that a significant portion of the population has access to healthcare services. However, middle and low SDI countries often have limited healthcare infrastructure, fewer financial resources, and a higher prevalence of other competing health priorities (34). The SDGs explicitly call for reducing inequalities within and among countries, as outlined in SDG 10. Achieving this goal requires addressing disparities in healthcare access, outcomes, and resources, particularly in resource-constrained settings. Our findings highlight the importance of strengthening healthcare systems in middle and low SDI regions by strategically allocating medical budgets to implement early screening programs, improving treatment quality, and expanding medical insurance coverage. These targeted interventions not only help to reduce TBL cancer mortality, disease severity, and economic burden but also play a critical role in bridging the gap between higher and lower SDI regions. By aligning with SDG 10′s mandate to promote equitable healthcare access and outcomes, these measures contribute to reducing global health disparities and ensuring more inclusive progress toward sustainable health systems. This approach underscores the need for international collaboration and resource-sharing to address persistent inequalities, ultimately supporting the global commitment to achieving health equity.

Moreover, we focus on changes in absolute numbers, rather than just rates, as these figures provide a clearer, more direct understanding of the magnitude of the disease burden. While changes in rates are important for epidemiological studies, the absolute numbers of deaths and DALYs offer more concrete evidence of the scale of the challenge faced by health systems, particularly in terms of resource allocation and prioritization (35). This is especially relevant for policymaking, where absolute numbers are often more critical in informing decisions regarding healthcare investments, interventions, and long-term strategies.

Economic evaluations further suggest that by 2050, the region facing the highest TBL cancer economic burden will shift from the high SDI region to the high-middle SDI region. While high SDI countries often have the financial flexibility and resources to allocate to comprehensive cancer prevention strategies, early detection, and advanced treatment options, the socioeconomic pressures arising from a growing disease burden are expected to increase (36). Additionally, as high-middle SDI countries experience economic growth, their healthcare expenditures and the value they place on life years (as reflected in the VSL) tend to increase (37). Moreover, high- and high-middle-SDI regions demonstrate the largest absolute economic burden in our results, this pattern primarily reflects system-level factors rather than a disproportionately higher disease burden (38). Health systems in these settings typically achieve greater diagnosis coverage, wider access to treatment, and longer survival, all of which increase medical spending and raise the monetized value of health losses (39). In addition, high-SDI countries often utilize more advanced—and therefore more costly—technologies for screening, diagnosis, and therapy, resulting in higher per-patient expenditures (40). These patterns remained stable across multiple elasticity scenarios economic projections, underscoring the robustness of the anticipated geographic and demographic shift in financial burden.

To the best of our knowledge, this is the first study to quantitatively explore the complex relationship among population aging indicators, health status, and the global burden of TBL cancer across diverse regions and sexes. This analysis offers a foundation for devising precise TBL cancer prevention and control strategies, tailored to the economic realities of diverse regions. Furthermore, it emphasizes the necessity of adjusting our response plans based on the latest disease burden data. By quantifying the economic burden of TBL cancer across various geographic and demographic contexts worldwide, we estimated the significant financial losses to society caused by this disease. This approach emphasizes the importance of using current data to inform and refine strategies for effective TBL cancer management and prevention. The findings and recommendations from this study contribute directly to achieving the SDGs by promoting equitable healthcare access, reducing health disparities, and supporting the development of sustainable and inclusive health systems.

We acknowledge certain limitations in our research, primarily the statistical uncertainties associated with the GBD study, which stem from the use of various subnational data sources. The decomposition approach used in this study showed the observed changes in deaths and DALYs into mathematical components. These components reflect the quantitative share of change rather than causal effects and therefore should not be interpreted as determinants of disease burden. Although the VSL approach quantifies the monetary and non-monetary impacts of TBL cancer, it does not account for broader economic effects on caregivers, including opportunity costs and financial strain on caregivers (41). Our reliance on the World Bank's 2023 income classification of countries may constrain the forward-looking accuracy of our analysis, particularly considering potential economic fluctuations. Additionally, our predictions of future economic burdens assume that current levels will remain stable, which may introduce inaccuracies. This approach does not consider the possible variations in economic conditions, advancements in healthcare, or shifts in population demographics that could significantly impact the economic landscape over time. In particular, future trends in TBL cancer incidence were not explicitly modeled in our projections. Instead, the economic burden estimates for 2030–2050 were derived under the assumption that age-specific disease rates observed in 2021 remain constant over time. As such, potential future changes in incidence driven by evolving risk-factor profiles, screening uptake, treatment innovations, or health system performance were not incorporated. Therefore, while our forecasts offer a valuable starting point for understanding the economic implications of TBL cancer, they should be interpreted with caution, as real-world dynamics may lead to deviations from these projections. Moreover, although the COVID-19 pandemic has been shown to disrupt cancer screening, diagnosis, and treatment pathways globally, potentially altering long-term incidence and mortality patterns (3), current evidence suggests that no immediate change in the burden of TBL cancer was observed during the pandemic period (5). Nevertheless, delayed diagnoses and interruptions in care may exert lagged effects on cancer outcomes that are not yet fully captured in available data. Continued population-level surveillance and longitudinal analyses will therefore be essential to assess the longer-term impact of the pandemic on TBL cancer burden.

## Conclusions

Overall, our study provides a comprehensive analysis of the global, regional, and sex-specific disease and economic burden of TBL cancer, offering novel insights into its key components and disparities. We identified adult population growth as the primary factor contributing to the increasing global burden of TBL cancer, while improvements in fatality and severity rates had a more limited impact, particularly among females and in lower SDI regions. Additionally, our findings highlight a sharp rise in the burden among females and a dramatic shift in the highest economic impact from high-SDI to high-middle SDI regions by 2050, underscoring the urgent need for targeted public health strategies. Policymakers should prioritize tailored prevention efforts, expanded access to early detection, and equitable healthcare interventions to mitigate the social and economic consequences of TBL cancer, particularly in vulnerable regions and populations.

## Data Availability

Publicly available datasets were analyzed in this study. This data can be found from the Global Health Data Exchange GBD (https://vizhub.healthdata.org/gbd-results/).
