# Supplementary material for: Global trends, decomposition analysis, inequality assessment, and economic projections of tracheal, bronchus, and lung cancer
Source: Front Public Health. 2026 Jan 26;13:1745506. doi: 10.3389/fpubh.2025.1745506 (PMC12883795; doi:10.3389/fpubh.2025.1745506)
Supplement: Supplementary file 1 [file Table_1.DOCX]

**Supplementary material**

**[Supplementary Table 1](#_Toc216118953)** [The deaths number and percentage change for tracheal, bronchus, and lung cancer in 5 SDI and 21 GBD regions between 1990 and 2021 2](#_Toc216118953)

**[Supplementary Table 2](#_Toc216118954)** [The deaths number and percentage change for tracheal, bronchus, and lung cancer in 5 SDI and 21 GBD regions between 1990 and 2021 in males. 3](#_Toc216118954)

**[Supplementary Table 3](#_Toc216118955)** [The DALYs number and percentage change for tracheal, bronchus, and lung cancer in 5 SDI and 21 GBD regions between 1990 and 2021 in males. 4](#_Toc216118955)

**[Supplementary Table 4](#_Toc216118956)** [The deaths number and percentage change for tracheal, bronchus, and lung cancer in 5 SDI and 21 GBD regions between 1990 and 2021 in females. 5](#_Toc216118956)

**[Supplementary Table 5](#_Toc216118957)** [The DALYs number and percentage change for tracheal, bronchus, and lung cancer in 5 SDI and 21 GBD regions between 1990 and 2021 in females. 6](#_Toc216118957)

**[Supplementary Table 6](#_Toc216118958)** [Decomposition of change in age‐related deaths for TBL cancer in 5 SDI and 21 GBD regions between 1990 and 2021. 7](#_Toc216118958)

**[Supplementary Table 7](#_Toc216118959)** [Decomposition of change in age‐related DALYs for TBL cancer in 5 SDI and 21 GBD regions between 1990 and 2021. 9](#_Toc216118959)

**[Supplementary Table 8](#_Toc216118960)** [Decomposition of change in age‐related deaths for TBL cancer in 5 SDI and 21 GBD regions between 1990 and 2021 in female. 11](#_Toc216118960)

**[Supplementary Table 9](#_Toc216118961)** [Decomposition of change in age‐related deaths for TBL cancer in 5 SDI and 21 GBD regions between 1990 and 2021 in male. 13](#_Toc216118961)

**[Supplementary Table 10](#_Toc216118962)** [Decomposition of change in age‐related DALYs for TBL cancer in 5 SDI and 21 GBD regions between 1990 and 2021 in female. 15](#_Toc216118962)

**[Supplementary Table 11](#_Toc216118963)** [Decomposition of change in age‐related DALYs for TBL cancer in 5 SDI and 21 GBD regions between 1990 and 2021 in male. 17](#_Toc216118963)

**[Supplementary Table 12](#_Toc216118964)** [AAPC of concentration indices of tracheal, bronchus, and lung cancer across 21 GBD region between 1990 and 2021 by gender. 19](#_Toc216118964)

**[Supplementary Table 13](#_Toc216118965)**  [Estimated economic burden of tracheal, bronchus, and lung cancer by SDI regions through 2050, billions of 2023 US$. (E=1.5) 20](#_Toc216118965)

**[Supplementary Table 14](#_Toc216118966)** [Estimated economic burden of tracheal, bronchus, and lung cancer by SDI regions through 2050, billions of 2023 US$. (E=1.0) 21](#_Toc216118966)

**[Supplementary Table 15](#_Toc216118967)** [Estimated economic burden of tracheal, bronchus, and lung cancer by country through 2050, millions of 2023 US$. 22](#_Toc216118967)

**[Supplementary Table 16](#_Toc216118968)** [Estimated economic burden of tracheal, bronchus, and lung cancer by country through 2050 by sex, millions of 2023 US$. 29](#_Toc216118968)

**[Supplementary Table 17](#_Toc216118969)** [Estimated economic burden of tracheal, bronchus, and lung cancer by country through 2050, millions of 2023 US$. (E=1.5) 36](#_Toc216118969)

**[Supplementary Table 18](#_Toc216118970)** [Estimated economic burden of tracheal, bronchus, and lung cancer by country through 2050, millions of 2023 US$. (E=1.0) 43](#_Toc216118970)

**[Supplementary Table 19](#_Toc216118971)** [Estimated economic burden of tracheal, bronchus, and lung cancer by country through 2050 by sex, millions of 2023 US$. (E=1.5) 50](#_Toc216118971)

**[Supplementary Table 20](#_Toc216118972)** [Estimated economic burden of tracheal, bronchus, and lung cancer by country through 2050 by sex, millions of 2023 US$. (E=1.0) 56](#_Toc216118972)

**[Supplementary section 1:](#_Toc216118973)** [The methodology of decomposed analysis. 64](#_Toc216118973)

**[Supplementary section 2:](#_Toc216118974)** [The methodology of economic burden analysis. 65](#_Toc216118974)

**Supplementary Table 1** The deaths number and percentage change for tracheal, bronchus, and lung cancer in 5 SDI and 21 GBD regions between 1990 and 2021

| **Location** | **Deaths number (in 1000), 95%UI** | | |
| --- | --- | --- | --- |
|  | **1990** | **2021** | **Total percentage change** |
| **Global** | 1080.13 (1023.33, 1135.56) | 2016.55 (1820.5, 2218.37) | 0.87 (0.64, 1.08) |
| **Socio-demographic index** |  |  |  |
| High SDI | 431.61 (415.25, 440.83) | 596.53 (543.55, 627.88) | 0.38 (0.30, 0.44) |
| High-middle SDI | 359.53 (336.76, 381.72) | 648.62 (570.10, 736.85) | 0.8 (0.54, 1.08) |
| Middle SDI | 222.73 (200.73, 245.88) | 605.10 (507.10, 698.72) | 1.72 (1.19, 2.28) |
| Low-middle SDI | 51.41 (45.5, 59.66) | 135.26 (122.97, 148.77) | 1.63 (1.15, 2.07) |
| Low SDI | 13.42 (11.16, 17.01) | 28.96 (24.64, 34.24) | 1.16 (0.81, 1.66) |
| **GBD Region** |  |  |  |
| **Southeast Asia, East Asia, and Oceania** | |  |  |
| East Asia | 286.46 (246.28, 327.3) | 834.11 (673.13, 1005.11) | 1.91 (1.21, 2.76) |
| Southeast Asia | 49.57 (43.83, 55.54) | 134.95 (110.67, 155.97) | 1.72 (1.12, 2.18) |
| Oceania | 0.48 (0.35, 0.71) | 1.27 (0.96, 1.77) | 1.67 (1.03, 2.51) |
| **Central Europe, Eastern Europe, and Central Asia** | |  |  |
| Central Asia | 13.36 (12.68, 14.06) | 10.98 (9.78, 12.15) | -0.18 (-0.28, -0.08) |
| Central Europe | 59.20 (57.30, 61.08) | 80.66 (74.87, 85.83) | 0.36 (0.26, 0.46) |
| Eastern Europe | 101.79 (99.34, 103.82) | 74.97 (68.12, 81.57) | -0.26 (-0.33, -0.2) |
| **High-income** |  |  |  |
| High-income Asia Pacific | 51.25 (48.77, 53.07) | 116.19 (99.37, 125.88) | 1.27 (1.04, 1.43) |
| Australasia | 7.9 (7.49, 8.29) | 12.26 (10.96, 13.47) | 0.55 (0.40, 0.71) |
| Western Europe | 206.75 (199.67, 211.7) | 247.74 (226.54, 261.74) | 0.2 (0.13, 0.25) |
| Southern Latin America | 13.34 (12.61, 14.12) | 17.31 (15.83, 18.87) | 0.3 (0.18, 0.44) |
| High-income North America | 173.47 (165.52, 178.67) | 197.32 (181.48, 207.76) | 0.14 (0.08, 0.18) |
| **Latin America and Caribbean** |  |  |  |
| Caribbean | 5.74 (5.44, 6.11) | 10.69 (9.46, 12.06) | 0.86 (0.64, 1.11) |
| Andean Latin America | 2.76 (2.39, 3.16) | 6.31 (4.91, 7.73) | 1.29 (0.73, 1.89) |
| Central Latin America | 11.91 (11.52, 12.22) | 24.35 (21.58, 27.54) | 1.04 (0.82, 1.32) |
| Tropical Latin America | 14.79 (14.23, 15.27) | 38.73 (36.05, 40.85) | 1.62 (1.47, 1.76) |
| **North Africa and Middle East** |  |  |  |
| North Africa and Middle East | 31.2 (25.96, 37.06) | 74.56 (65.32, 86.14) | 1.39 (0.91, 2.06) |
| **South Asia** |  |  |  |
| South Asia | 34.92 (30.31, 40.23) | 100.18 (84.75, 113.99) | 1.87 (1.17, 2.51) |
| **Sub-Saharan Africa** |  |  |  |
| Central Sub-Saharan Africa | 2.25 (1.62, 3.39) | 5.08 (3.59, 7.65) | 1.26 (0.63, 2.19) |
| Southern Sub-Saharan Africa | 4.62 (4.02, 5.55) | 10.87 (9.83, 12.06) | 1.35 (0.99, 1.81) |
| Eastern Sub-Saharan Africa | 5.36 (4.53, 6.63) | 10.33 (8.93, 12.36) | 0.93 (0.50, 1.45) |
| Western Sub-Saharan Africa | 3.02 (2.52, 3.57) | 7.69 (6.37, 9.21) | 1.55 (1.09, 2.17) |

SDI: Socio-demographic index; UIs: uncertainty intervals

**Supplementary Table 2** The deaths number and percentage change for tracheal, bronchus, and lung cancer in 5 SDI and 21 GBD regions between 1990 and 2021 in males.

| **Location** | **Deaths number (in 1000), 95%UI** | | |
| --- | --- | --- | --- |
|  | **1990** | **2021** | **Total percentage change** |
| **Global** | 798.82 (750.84, 853.58) | 1342.54 (1180.49, 1513.56) | 0.68 (0.43, 0.94) |
| **Socio-demographic index** |  |  |  |
| High SDI | 306.85 (298.38, 312.54) | 363.23 (338.42, 380.10) | 0.18 (0.13, 0.23) |
| High-middle SDI | 282.65 (261.16, 302.79) | 449.52 (375.98, 529.38) | 0.59 (0.28, 0.91) |
| Middle SDI | 158.31 (137.98, 180.51) | 413.82 (329.86, 499.86) | 1.61 (0.96, 2.32) |
| Low-middle SDI | 39.27 (33.84, 47.5) | 93.83 (85.70, 103.24) | 1.39 (0.89, 1.90) |
| Low SDI | 10.60 (8.53, 14.07) | 20.73 (17.41, 25.04) | 0.96 (0.60, 1.51) |
| **GBD Region** |  |  |  |
| **Southeast Asia, East Asia, and Oceania** | |  |  |
| East Asia | 197.95 (157.94, 237.99) | 558.49 (416.20, 714.42) | 1.82 (0.91, 2.95) |
| Southeast Asia | 35.69 (30.78, 41.89) | 91.88 (76.40, 108.06) | 1.57 (0.94, 2.13) |
| Oceania | 0.36 (0.26, 0.53) | 0.91 (0.66, 1.31) | 1.52 (0.90, 2.45) |
| **Central Europe, Eastern Europe, and Central Asia** | |  |  |
| Central Asia | 10.74 (10.13, 11.37) | 8.59 (7.58, 9.54) | -0.20 (-0.30, -0.10) |
| Central Europe | 48.86 (47.19, 50.59) | 55.02 (50.52, 58.85) | 0.13 (0.03, 0.22) |
| Eastern Europe | 84.58 (82.59, 86.33) | 59.30 (53.03, 65.36) | -0.30 (-0.37, -0.23) |
| **High-income** |  |  |  |
| High-income Asia Pacific | 37.34 (35.93, 38.55) | 81.28 (73.05, 86.28) | 1.18 (1.01, 1.30) |
| Australasia | 5.68 (5.36, 6.00) | 7.12 (6.40, 7.88) | 0.25 (0.13, 0.40) |
| Western Europe | 163.03 (157.94, 166.8) | 160.7 (149.08, 169.03) | -0.01 (-0.07, 0.03) |
| Southern Latin America | 10.85 (10.24, 11.51) | 11.23 (10.29, 12.22) | 0.03 (-0.07, 0.15) |
| High-income North America | 111.6 (107.6, 114.87) | 106.19 (99.78, 111.00) | -0.05 (-0.09, -0.01) |
| **Latin America and Caribbean** |  |  |  |
| Caribbean | 4.13 (3.89, 4.46) | 6.82 (6.02, 7.68) | 0.65 (0.45, 0.88) |
| Andean Latin America | 1.81 (1.54, 2.10) | 3.43 (2.68, 4.21) | 0.9 (0.42, 1.41) |
| Central Latin America | 7.88 (7.63, 8.10) | 14.31 (12.52, 16.33) | 0.82 (0.6, 1.07) |
| Tropical Latin America | 10.77 (10.38, 11.13) | 22.24 (20.81, 23.43) | 1.07 (0.94, 1.18) |
| **North Africa and Middle East** |  |  |  |
| North Africa and Middle East | 27.03 (22.13, 32.52) | 59.49 (51.37, 70.1) | 1.2 (0.73, 1.91) |
| **South Asia** |  |  |  |
| South Asia | 28.8 (24.49, 34.02) | 71.78 (58.26, 83.17) | 1.49 (0.77, 2.13) |
| **Sub-Saharan Africa** |  |  |  |
| Central Sub-Saharan Africa | 1.76 (1.20, 2.88) | 3.72 (2.47, 5.97) | 1.11 (0.52, 2.03) |
| Southern Sub-Saharan Africa | 3.35 (2.84, 4.08) | 7.18 (6.33, 8.12) | 1.14 (0.79, 1.58) |
| Eastern Sub-Saharan Africa | 4.25 (3.43, 5.54) | 7.23 (6.14, 8.74) | 0.70 (0.21, 1.29) |
| Western Sub-Saharan Africa | 2.36 (1.93, 2.81) | 5.62 (4.69, 6.75) | 1.38 (0.95, 2.06) |

DALYs: disability-adjusted life-years SDI: Socio-demographic index; UIs: uncertainty intervals

| **Location** | **DALYs number (in 1000), 95%UI** | | |
| --- | --- | --- | --- |
|  | **1990** | **2021** | **Total percentage change** |
| **Global** | 21284.74 (19931.20, 22827.66) | 31439.63 (27496.3, 35558.59) | 0.48 (0.25, 0.72) |
| **Socio-demographic index** |  |  |  |
| High SDI | 7471.71 (7310.96, 7604.34) | 7433.01 (7031.32, 7771.99) | -0.01 (-0.05, 0.04) |
| High-middle SDI | 7888.54 (7264.32, 8465.29) | 10680.27 (8936.76, 12643.99) | 0.35 (0.09, 0.64) |
| Middle SDI | 4474.93 (3902.48, 5109.25) | 10150.61 (8122.26, 12267.23) | 1.27 (0.69, 1.90) |
| Low-middle SDI | 1117.09 (967.21, 1355.20) | 2556.97 (2312.98, 2827.26) | 1.29 (0.80, 1.79) |
| Low SDI | 301.02 (242.87, 402.42) | 585.07 (485.74, 713.63) | 0.94 (0.58, 1.51) |
| **GBD Region** |  |  |  |
| **Southeast Asia, East Asia, and Oceania** | |  |  |
| East Asia | 5558.78 (4416.73, 6711.29) | 13138.85 (9701.86, 17009.16) | 1.36 (0.58, 2.33) |
| Southeast Asia | 991.25 (858.93, 1162) | 2430.48 (2006.73, 2890.12) | 1.45 (0.85, 2.00) |
| Oceania | 10.30 (7.32, 15.29) | 25.59 (18.61, 36.89) | 1.48 (0.84, 2.40) |
| **Central Europe, Eastern Europe, and Central Asia** | |  |  |
| Central Asia | 329.84 (310.62, 349.70) | 241.65 (212.29, 269.97) | -0.27 (-0.36, -0.18) |
| Central Europe | 1382.07 (1334.87, 1432.77) | 1311.97 (1203.62, 1404.59) | -0.05 (-0.13, 0.03) |
| Eastern Europe | 2488.19 (2425.84, 2540.51) | 1543.37 (1372.38, 1710.69) | -0.38 (-0.45, -0.31) |
| **High-income** |  |  |  |
| High-income Asia Pacific | 872.69 (844.05, 901.69) | 1418.68 (1300.97, 1497.89) | 0.63 (0.51, 0.72) |
| Australasia | 134.10 (126.81, 141.28) | 145.03 (132.84, 158.82) | 0.08 (-0.02, 0.20) |
| Western Europe | 3944.05 (3845.08, 4029.92) | 3345.99 (3147.53, 3499.13) | -0.15 (-0.19, -0.11) |
| Southern Latin America | 292.74 (274.91, 311.71) | 261.60 (239.04, 285.65) | -0.11 (-0.19, 0.00) |
| High-income North America | 2688.57 (2609.11, 2761.83) | 2231.54 (2120.53, 2318.70) | -0.17 (-0.20, -0.14) |
| **Latin America and Caribbean** |  |  |  |
| Caribbean | 100.96 (94.62, 109.93) | 160.35 (140.58, 182.36) | 0.59 (0.38, 0.81) |
| Andean Latin America | 46.96 (40.08, 54.9) | 80.39 (62.09, 99.98) | 0.71 (0.28, 1.21) |
| Central Latin America | 204.68 (198.57, 210.17) | 340.16 (295.76, 390.03) | 0.66 (0.44, 0.91) |
| Tropical Latin America | 295.95 (286.18, 305.64) | 533.33 (502.96, 561.88) | 0.80 (0.70, 0.90) |
| **North Africa and Middle East** |  |  |  |
| North Africa and Middle East | 765.11 (622.11, 922.21) | 1562.84 (1351.81, 1833.32) | 1.04 (0.60, 1.70) |
| **South Asia** |  |  |  |
| South Asia | 843.01 (717.45, 994.14) | 1997.8 (1621.15, 2329.55) | 1.37 (0.67, 2.00) |
| **Sub-Saharan Africa** |  |  |  |
| Central Sub-Saharan Africa | 50.81 (34.47, 82.94) | 110.46 (73.39, 179.72) | 1.17 (0.54, 2.17) |
| Southern Sub-Saharan Africa | 99.64 (84.67, 120.76) | 205.92 (181.05, 233.94) | 1.07 (0.73, 1.51) |
| Eastern Sub-Saharan Africa | 122.42 (98.08, 162.61) | 205.16 (172.95, 253.28) | 0.68 (0.18, 1.31) |
| Western Sub-Saharan Africa | 62.60 (51.30, 74.57) | 148.47 (121.53, 181.00) | 1.37 (0.92, 2.09) |

**Supplementary Table 3** The DALYs number and percentage change for tracheal, bronchus, and lung cancer in 5 SDI and 21 GBD regions between 1990 and 2021 in males.

DALYs: disability-adjusted life-years SDI: Socio-demographic index; UIs: uncertainty intervals

**Supplementary Table 4** The deaths number and percentage change for tracheal, bronchus, and lung cancer in 5 SDI and 21 GBD regions between 1990 and 2021 in females.

| **Location** | **Deaths number (in 1000), 95%UI** | | |
| --- | --- | --- | --- |
|  | **1990** | **2021** | **Total percentage change** |
| **Global** | 281.31 (259.31, 301.72) | 674.01 (595.39, 747.51) | 1.4 (1.11, 1.68) |
| **Socio-demographic index** |  |  |  |
| High SDI | 124.76 (116.35, 128.95) | 233.30 (203.96, 249.46) | 0.87 (0.73, 0.95) |
| High-middle SDI | 76.89 (69.00, 85.54) | 199.10 (168.79, 234.49) | 1.59 (1.11, 2.14) |
| Middle SDI | 64.42 (56.11, 74.27) | 191.28 (162.22, 224.16) | 1.97 (1.40, 2.64) |
| Low-middle SDI | 12.14 (10.67, 13.98) | 41.43 (36.07, 46.97) | 2.41 (1.86, 3.1) |
| Low SDI | 2.82 (2.29, 3.45) | 8.23 (6.75, 9.61) | 1.92 (1.17, 2.73) |
| **GBD Region** |  |  |  |
| **Southeast Asia, East Asia, and Oceania** | |  |  |
| East Asia | 88.50 (73.10, 105.92) | 275.62 (218.15, 338.98) | 2.11 (1.32, 3.18) |
| Southeast Asia | 13.88 (11.83, 16.58) | 43.06 (33.43, 51.66) | 2.10 (1.53, 2.88) |
| Oceania | 0.12 (0.08, 0.18) | 0.36 (0.26, 0.52) | 2.10 (1.34, 3.17) |
| **Central Europe, Eastern Europe, and Central Asia** | |  |  |
| Central Asia | 2.62 (2.47, 2.77) | 2.39 (2.10, 2.69) | -0.09 (-0.19, 0.04) |
| Central Europe | 10.34 (9.82, 10.84) | 25.64 (23.45, 27.75) | 1.48 (1.27, 1.69) |
| Eastern Europe | 17.21 (16.57, 17.69) | 15.66 (14.07, 17.27) | -0.09 (-0.18, 0.00) |
| **High-income** |  |  |  |
| High-income Asia Pacific | 13.91 (12.82, 14.67) | 34.91 (26.76, 40.10) | 1.51 (1.09, 1.78) |
| Australasia | 2.22 (2.08, 2.34) | 5.14 (4.47, 5.64) | 1.31 (1.08, 1.52) |
| Western Europe | 43.71 (41.06, 45.20) | 87.04 (76.42, 93.63) | 0.99 (0.85, 1.09) |
| Southern Latin America | 2.49 (2.31, 2.67) | 6.09 (5.40, 6.75) | 1.44 (1.19, 1.70) |
| High-income North America | 61.87 (57.64, 64.19) | 91.12 (80.47, 96.98) | 0.47 (0.39, 0.53) |
| **Latin America and Caribbean** |  |  |  |
| Caribbean | 1.61 (1.52, 1.71) | 3.87 (3.34, 4.42) | 1.40 (1.10, 1.77) |
| Andean Latin America | 0.95 (0.84, 1.08) | 2.88 (2.16, 3.59) | 2.04 (1.28, 2.85) |
| Central Latin America | 4.03 (3.87, 4.16) | 10.04 (8.63, 11.56) | 1.49 (1.13, 1.89) |
| Tropical Latin America | 4.02 (3.79, 4.21) | 16.48 (15.00, 17.57) | 3.10 (2.83, 3.35) |
| **North Africa and Middle East** |  |  |  |
| North Africa and Middle East | 4.18 (3.38, 5.21) | 15.08 (12.70, 17.48) | 2.61 (1.60, 3.88) |
| **South Asia** |  |  |  |
| South Asia | 6.12 (5.00, 7.05) | 28.4 (24.15, 33.19) | 3.64 (2.68, 5.06) |
| **Sub-Saharan Africa** |  |  |  |
| Central Sub-Saharan Africa | 0.49 (0.36, 0.66) | 1.36 (0.99, 1.83) | 1.80 (0.69, 3.30) |
| Southern Sub-Saharan Africa | 1.27 (1.00, 1.63) | 3.69 (3.24, 4.17) | 1.90 (1.18, 2.99) |
| Eastern Sub-Saharan Africa | 1.11 (0.91, 1.36) | 3.09 (2.41, 3.78) | 1.80 (0.98, 2.79) |
| Western Sub-Saharan Africa | 0.65 (0.52, 0.80) | 2.07 (1.59, 2.50) | 2.18 (1.36, 3.14) |

SDI: Socio-demographic index; UIs: uncertainty intervals

**Supplementary Table 5** The DALYs number and percentage change for tracheal, bronchus, and lung cancer in 5 SDI and 21 GBD regions between 1990 and 2021 in females.

| **Location** | **DALYs number (in 1000), 95%UI** | | |
| --- | --- | --- | --- |
|  | **1990** | **2021** | **Total percentage change** |
| **Global** | 7175.10 (6643.72, 7743.27) | 15096.64 (13599.78, 16694.42) | 1.10 (0.85, 1.38) |
| **Socio-demographic index** |  |  |  |
| High SDI | 2926.07 (2782.67, 3004.50) | 4616.98 (4181.53, 4866.12) | 0.58 (0.49, 0.64) |
| High-middle SDI | 2003.36 (1789.17, 2250.41) | 4512.14 (3826.31, 5305.99) | 1.25 (0.82, 1.77) |
| Middle SDI | 1800.55 (1567.18, 2080.32) | 4587.19 (3896.96, 5367.38) | 1.55 (1.05, 2.15) |
| Low-middle SDI | 355.19 (310.83, 408.38) | 1129.16 (981.98, 1284.91) | 2.18 (1.66, 2.83) |
| Low SDI | 82.59 (67.22, 102.46) | 235.62 (192.07, 276.00) | 1.85 (1.09, 2.64) |
| **GBD Region** |  |  |  |
| **Southeast Asia, East Asia, and Oceania** | |  |  |
| East Asia | 2432.02 (1985.15, 2926.32) | 6253.93 (4905.05, 7766.65) | 1.57 (0.89, 2.52) |
| Southeast Asia | 396.61 (334.80, 473.96) | 1125.88 (865.00, 1373.02) | 1.84 (1.32, 2.57) |
| Oceania | 3.36 (2.18, 5.13) | 10.43 (7.39, 15.12) | 2.10 (1.29, 3.24) |
| **Central Europe, Eastern Europe, and Central Asia** | |  |  |
| Central Asia | 72.73 (68.90, 76.44) | 65.62 (57.54, 75.23) | -0.10 (-0.20, 0.03) |
| Central Europe | 267.40 (255.23, 280.12) | 578.42 (530.23, 626.79) | 1.16 (0.98, 1.35) |
| Eastern Europe | 425.84 (412.07, 436.44) | 359.93 (323.41, 398.96) | -0.15 (-0.24, -0.06) |
| **High-income** |  |  |  |
| High-income Asia Pacific | 312.82 (294.45, 328.04) | 540.68 (438.17, 604.17) | 0.73 (0.47, 0.90) |
| Australasia | 52.89 (50.05, 55.45) | 106.27 (95.20, 114.90) | 1.01 (0.84, 1.17) |
| Western Europe | 983.93 (940.55, 1010.86) | 1792.17 (1631.75, 1898.53) | 0.82 (0.72, 0.90) |
| Southern Latin America | 62.41 (58.25, 66.79) | 138.09 (124.36, 152.09) | 1.21 (0.98, 1.46) |
| High-income North America | 1475.07 (1399.51, 1518.62) | 1845.75 (1683.61, 1940.73) | 0.25 (0.19, 0.30) |
| **Latin America and Caribbean** |  |  |  |
| Caribbean | 39.66 (37.52, 42.06) | 89.68 (77.55, 102.60) | 1.26 (0.97, 1.61) |
| Andean Latin America | 25.52 (22.22, 29.33) | 68.91 (51.48, 86.72) | 1.70 (1.02, 2.42) |
| Central Latin America | 105.97 (102.22, 109.04) | 240.52 (205.89, 278.19) | 1.27 (0.92, 1.65) |
| Tropical Latin America | 109.83 (104.63, 114.65) | 404.59 (376.24, 429.25) | 2.68 (2.45, 2.91) |
| **North Africa and Middle East** |  |  |  |
| North Africa and Middle East | 122.66 (100.27, 155.43) | 400.16 (335.38, 465.84) | 2.26 (1.30, 3.39) |
| **South Asia** |  |  |  |
| South Asia | 187.07 (155.40, 215.38) | 796.50 (677.48, 931.23) | 3.26 (2.39, 4.51) |
| **Sub-Saharan Africa** |  |  |  |
| Central Sub-Saharan Africa | 14.46 (10.78, 19.85) | 39.51 (28.59, 53.19) | 1.73 (0.64, 3.21) |
| Southern Sub-Saharan Africa | 35.09 (27.83, 44.10) | 94.75 (83.04, 107.51) | 1.70 (1.08, 2.70) |
| Eastern Sub-Saharan Africa | 31.82 (26.02, 39.60) | 87.22 (67.33, 107.82) | 1.74 (0.91, 2.72) |
| Western Sub-Saharan Africa | 17.94 (14.21, 22.25) | 57.63 (43.25, 71.36) | 2.21 (1.35, 3.18) |

DALYs: disability-adjusted life-years SDI: Socio-demographic index; UIs: uncertainty intervals

**Supplementary Table 6** Decomposition of change in age‐related deaths for TBL cancer in 5 SDI and 21 GBD regions between 1990 and 2021.

| Location | Change in Death (in 1000) number (%)^a^ | | | |  |
| --- | --- | --- | --- | --- | --- |
|  | Population size | Population aging | Incidence | Case fatality | Total |
| **Global** | 882.47 (94.18%) | 337.95 (36.07%) | -118.58 (-12.65%) | -164.80 (-17.59%) | 937.04 (100.00%) |
| **SDI Region** |  |  |  |  |  |
| High SDI | 180.3 (109.26%) | 145.21 (87.99%) | -84.21 (-51.03%) | -76.27 (-46.22%) | 165.02 (100.00%) |
| High-middle SDI | 214.83 (74.20%) | 130.67 (45.13%) | 16.54 (5.71%) | -72.51 (-25.04%) | 289.54 (100.00%) |
| Middle SDI | 252.16 (65.88%) | 131.28 (34.30%) | 51.39 (13.43%) | -52.06 (-13.60%) | 382.77 (100.00%) |
| Low-middle SDI | 63.51 (75.94%) | 11.78 (14.09%) | 9.77 (11.69%) | -1.44 (-1.72%) | 83.63 (100.00%) |
| Low SDI | 17.67 (114.47%) | -1.15 (-7.44%) | -0.93 (-6.02%) | -0.16 (-1.02%) | 15.44 (100.00%) |
| **GBD regions** |  |  |  |  |  |
| **Southeast Asia, East Asia, and Oceania** |  |  |  |  |  |
| East Asia | 267.87 (48.84%) | 243.71 (44.44%) | 141.55 (25.81%) | -104.69 (-19.09%) | 548.44 (100.00%) |
| Southeast Asia | 60.39 (70.82%) | 22.64 (26.55%) | 5.10 (5.99%) | -2.86 (-3.35%) | 85.28 (100.00%) |
| Oceania | 0.72 (90.71%) | 0.04 (5.35%) | 0.04 (4.61%) | -0.01 (-0.66%) | 0.79 (100.00%) |
| **Central Europe, Eastern Europe, and Central Asia** | | | | | |
| Central Asia | 7.05 (301.46%) | 0.73 (31.36%) | -9.89 (-422.53%) | -0.24 (-10.28%) | -2.34 (100.00%) |
| Central Europe | 7.46 (34.74%) | 16.64 (77.46%) | 1.68 (7.83%) | -4.31 (-20.04%) | 21.49 (100.00%) |
| Eastern Europe | 3.90 (14.58%) | 14.12 (52.77%) | -37.05 (-138.47%) | -7.73 (-28.87%) | -26.75 (100.00%) |
| **High-income** |  |  |  |  |  |
| High-income Asia Pacific | 22.68 (34.90%) | 52.01 (80.05%) | 6.04 (9.29%) | -15.75 (-24.24%) | 64.98 (100.00%) |
| Australasia | 5.5 (126.07%) | 2.8 (64.15%) | -1.84 (-42.21%) | -2.09 (-48.00%) | 4.36 (100.00%) |
| Western Europe | 51.63 (125.78%) | 49.53 (120.66%) | -23.59 (-57.47%) | -36.52 (-88.97%) | 41.05 (100.00%) |
| Southern Latin America | 7.66 (192.73%) | 2.13 (53.65%) | -4.96 (-124.77%) | -0.86 (-21.60%) | 3.97 (100.00%) |
| High-income North America | 69.32 (290.54%) | 50.06 (209.84%) | -74.02 (-310.25%) | -21.50 (-90.12%) | 23.86 (100.00%) |
| **Latin America and Caribbean** |  |  |  |  |  |
| Caribbean | 4.19 (84.51%) | 1.82 (36.78%) | -0.37 (-7.54%) | -0.68 (-13.75%) | 4.96 (100.00%) |
| Andean Latin America | 3.71 (104.20%) | 1.04 (29.10%) | -0.97 (-27.41%) | -0.21 (-5.89%) | 3.56 (100.00%) |
| Central Latin America | 14.17 (113.75%) | 6.31 (50.69%) | -7.31 (-58.71%) | -0.71 (-5.73%) | 12.46 (100.00%) |
| Tropical Latin America | 17.97 (75.09%) | 8.63 (36.07%) | -1.77 (-7.42%) | -0.90 (-3.75%) | 23.93 (100.00%) |
| **North Africa and Middle East** |  |  |  |  |  |
| North Africa and Middle East | 46.81 (108.10%) | 3.97 (9.17%) | -6.60 (-15.24%) | -0.88 (-2.03%) | 43.30 (100.00%) |
| **South Asia** |  |  |  |  |  |
| South Asia | 47.70 (73.29%) | 11.20 (17.21%) | 7.50 (11.53%) | -1.32 (-2.03%) | 65.08 (100.00%) |
| **Sub-Saharan Africa** |  |  |  |  |  |
| Central Sub-Saharan Africa | 3.45 (122.21%) | -0.31 (-10.89%) | -0.30 (-10.62%) | -0.02 (-0.70%) | 2.82 (100.00%) |
| Eastern Sub-Saharan Africa | 5.08 (81.38%) | 0.53 (8.57%) | 0.73 (11.74%) | -0.11 (-1.69%) | 6.24 (100.00%) |
| Southern Sub-Saharan Africa | 7.01 (142.11%) | -0.69 (-14.06%) | -1.32 (-26.71%) | -0.07 (-1.35%) | 4.94 (100.00%) |
| Western Sub-Saharan Africa | 4.71 (101.18%) | -0.79 (-16.89%) | 0.77 (16.54%) | -0.04 (-0.83%) | 4.65 (100.00%) |

^a^ Percent in parentheses indicates the relative change of tracheal, bronchus, and lung cancer deaths associated with each decomposed factor compared with the total level. TBL cancer: tracheal, bronchus, and lung cancer; SDI: Socio-demographic index.

**Supplementary Table 7** Decomposition of change in age‐related DALYs for TBL cancer in 5 SDI and 21 GBD regions between 1990 and 2021.

| Location | Change in DALY (in 1000) number (%)^a^ | | | | | |
| --- | --- | --- | --- | --- | --- | --- |
|  | Population size | Population aging | Prevalence | Case fatality disease severity | | Total |
| **Global** | 21876.65 (120.73%) | 6790.52 (37.47%) | 1701.35 (9.39%) | -12247.85 (-67.59%) | 18120.67 (100.00%) | |
| **SDI Region** |  |  |  |  |  | |
| High SDI | 4047.16 (243.91%) | 2642.02 (159.22%) | 664.15 (40.03%) | -5694.01 (-343.15%) | 1659.32 (100.00%) | |
| High-middle SDI | 5520.33 (103.52%) | 2776.54 (52.07%) | 2404.73 (45.10%) | -5369.08 (-100.69%) | 5332.52 (100.00%) | |
| Middle SDI | 6612.71 (77.88%) | 2818.86 (33.20%) | 2518.56 (29.66%) | -3459.64 (-40.75%) | 8490.49 (100.00%) | |
| Low-middle SDI | 1760.19 (80.09%) | 245.81 (11.18%) | 319.91 (14.56%) | -128.04 (-5.83%) | 2197.87 (100.00%) | |
| Low SDI | 499.68 (116.21%) | -32.53 (-7.57%) | -16.39 (-3.81%) | -20.77 (-4.83%) | 429.99 (100.00%) | |
| **GBD regions** |  |  |  |  |  | |
| **Southeast Asia, East Asia, and Oceania** |  |  |  |  |  | |
| East Asia | 6955.98 (60.71%) | 5113.89 (44.63%) | 6768.1 (59.07%) | -7380.82 (-64.42%) | 11457.14 (100.00%) | |
| Southeast Asia | 1628.98 (75.36%) | 549.61 (25.43%) | 205.7 (9.52%) | -222.59 (-10.30%) | 2161.7 (100.00%) | |
| Oceania | 20.16 (91.77%) | 1.09 (4.94%) | 1.35 (6.15%) | -0.63 (-2.87%) | 21.96 (100.00%) | |
| **Central Europe, Eastern Europe, and Central Asia** | | | | | | |
| Central Asia | 205.95 (221.65%) | 29.29 (31.52%) | -307.41 (-330.85%) | -20.74 (-22.33%) | -92.92 (100.00%) | |
| Central Europe | 190.57 (78.57%) | 302.38 (124.67%) | 109.07 (44.97%) | -359.47 (-148.20%) | 242.55 (100.00%) | |
| Eastern Europe | 105.38 (10.47%) | 293.71 (29.20%) | -877.11 (-87.19%) | -527.98 (-52.48%) | -1006.00 (100.00%) | |
| **High-income** |  |  |  |  |  | |
| High-income Asia Pacific | 453.78 (58.44%) | 756.87 (97.48%) | 584.01 (75.22%) | -1018.2 (-131.14%) | 776.45 (100.00%) | |
| Australasia | 125.37 (194.62%) | 52.02 (80.75%) | 38.23 (59.34%) | -151.2 (-234.72%) | 64.42 (100.00%) | |
| Western Europe | 1180.57 (551.93%) | 851.79 (398.23%) | 1121.49 (524.31%) | -2939.95 (-1374.47%) | 213.9 (100.00%) | |
| Southern Latin America | 190.35 (426.50%) | 36.49 (81.75%) | -122.81 (-275.17%) | -59.4 (-133.08%) | 44.63 (100.00%) | |
| High-income North America | 1554.94 (1825.67%) | 1092.72 (1282.98%) | -1224.11 (-1437.25%) | -1508.72 (-1771.41%) | -85.17 (100.00%) | |
| **Latin America and Caribbean** |  |  |  |  |  | |
| Caribbean | 100.12 (91.26%) | 37.28 (33.98%) | 14.52 (13.23%) | -42.21 (-38.47%) | 109.71 (100.00%) | |
| Andean Latin America | 91.25 (118.84%) | 20.13 (26.21%) | -20.15 (-26.25%) | -14.44 (-18.81%) | 76.78 (100.00%) | |
| Central Latin America | 347.9 (128.33%) | 129.54 (47.78%) | -136.29 (-50.27%) | -70.05 (-25.84%) | 271.1 (100.00%) | |
| Tropical Latin America | 459.24 (86.41%) | 180.11 (33.89%) | -38.91 (-7.32%) | -68.96 (-12.97%) | 531.48 (100.00%) | |
| **North Africa and Middle East** |  |  |  |  |  | |
| North Africa and Middle East | 1274.46 (118.97%) | 98.8 (9.22%) | -203.63 (-19.01%) | -98.41 (-9.19%) | 1071.22 (100.00%) | |
| **South Asia** |  |  |  |  |  | |
| South Asia | 1357.46 (77.48%) | 230.41 (13.15%) | 270.39 (15.43%) | -106.28 (-6.07%) | 1751.99 (100.00%) | |
| **Sub-Saharan Africa** |  |  |  |  |  | |
| Central Sub-Saharan Africa | 100.38 (119.59%) | -7.02 (-8.36%) | -5.98 (-7.12%) | -3.44 (-4.10%) | 83.94 (100.00%) | |
| Eastern Sub-Saharan Africa | 143.68 (86.68%) | 17.02 (10.27%) | 9.11 (5.49%) | -4.05 (-2.45%) | 165.75 (100.00%) | |
| Southern Sub-Saharan Africa | 199.17 (146.54%) | -16.59 (-12.21%) | -36.28 (-26.69%) | -10.39 (-7.64%) | 135.92 (100.00%) | |
| Western Sub-Saharan Africa | 125.22 (100.89%) | -16.04 (-12.92%) | 18.38 (14.81%) | -3.44 (-2.78%) | 124.11 (100.00%) | |

^a^ Percent in parentheses indicates the relative change of tracheal, bronchus, and lung cancer deaths associated with each decomposed factor compared with the total level. TBL cancer: tracheal, bronchus, and lung cancer; SDI: Socio-demographic index; DALYs: disability-adjusted life-years.

**Supplementary Table 8** Decomposition of change in age‐related deaths for TBL cancer in 5 SDI and 21 GBD regions between 1990 and 2021 in female.

| Location | Change in death, no, in thousands (%)^a^ | | | | |
| --- | --- | --- | --- | --- | --- |
|  | Population size | Population aging | Incidence | Case fatality | Total |
| **Global** | 264.02 (67.21%) | 92.4 (23.52%) | 90.15 (22.95%) | -53.73 (-13.68%) | 392.83 (100.00%) |
| **SDI region** |  |  |  |  |  |
| High SDI | 56.95 (52.45%) | 42.61 (39.25%) | 34.08 (31.39%) | -25.07 (-23.09%) | 108.56 (100.00%) |
| High-middle SDI | 55.37 (45.25%) | 31.42 (25.68%) | 58.03 (47.43%) | -22.45 (-18.35%) | 122.36 (100.00%) |
| Middle SDI | 79.24 (62.40%) | 39.41 (31.04%) | 26.13 (20.58%) | -17.80 (-14.01%) | 126.99 (100.00%) |
| Low-middle SDI | 17.93 (61.44%) | 3.96 (13.56%) | 7.77 (26.64%) | -0.48 (-1.65%) | 29.18 (100.00%) |
| Low SDI | 4.39 (81.95%) | -0.14 (-2.63%) | 1.16 (21.57%) | -0.05 (-0.90%) | 5.35 (100.00%) |
| **GBD regions** |  |  |  |  |  |
| **Southeast Asia, East Asia, and Oceania** |  |  |  |  |  |
| East Asia | 89.67 (47.85%) | 76.31 (40.72%) | 58.61 (31.27%) | -37.18 (-19.84%) | 187.41 (100.00%) |
| Southeast Asia | 17.83 (61.18%) | 7.19 (24.68%) | 5.11 (17.53%) | -0.99 (-3.38%) | 29.15 (100.00%) |
| Oceania | 0.20 (79.39%) | 0.01 (2.66%) | 0.05 (18.85%) | 0.00 (-0.91%) | 0.25 (100.00%) |
| **Central Europe, Eastern Europe, and Central Asia** |  |  |  |  |  |
| Central Asia | 1.36 (626.45%) | 0.01 (6.81%) | -1.50 (-693.89%) | -0.06 (-25.75%) | -0.22 (100.00%) |
| Central Europe | 1.85 (12.06%) | 4.5 (29.42%) | 10.18 (66.54%) | -1.23 (-8.02%) | 15.30 (100.00%) |
| Eastern Europe | 0.61 (40.18%) | 2.47 (161.75%) | -2.97 (-194.60%) | -1.64 (-107.34%) | -1.53 (100.00%) |
| **High-income** |  |  |  |  |  |
| High-income Asia Pacific | 6.39 (30.39%) | 15.57 (74.11%) | 3.87 (18.40%) | -4.81 (-22.90%) | 21.01 (100.00%) |
| Australasia | 1.91 (65.58%) | 0.78 (26.71%) | 0.99 (33.83%) | -0.76 (-26.13%) | 2.91 (100.00%) |
| Western Europe | 13.46 (31.05%) | 11.26 (25.98%) | 29.75 (68.64%) | -11.12 (-25.67%) | 43.34 (100.00%) |
| Southern Latin America | 1.98 (55.12%) | 0.63 (17.52%) | 1.25 (34.66%) | -0.26 (-7.30%) | 3.59 (100.00%) |
| High-income North America | 26.65 (91.10%) | 16.13 (55.15%) | -4.25 (-14.54%) | -9.27 (-31.70%) | 29.25 (100.00%) |
| **Latin America and Caribbean** |  |  |  |  |  |
| Caribbean | 1.34 (59.53%) | 0.68 (29.97%) | 0.35 (15.70%) | -0.12 (-5.19%) | 2.26 (100.00%) |
| Andean Latin America | 1.47 (76.09%) | 0.44 (22.70%) | 0.11 (5.74%) | -0.09 (-4.53%) | 1.93 (100.00%) |
| Central Latin America | 5.40 (89.87%) | 2.52 (41.90%) | -1.64 (-27.26%) | -0.27 (-4.52%) | 6.01 (100.00%) |
| Tropical Latin America | 6.43 (51.64%) | 3.22 (25.84%) | 3.13 (25.15%) | -0.33 (-2.63%) | 12.45 (100.00%) |
| **North Africa and Middle East** |  |  |  |  |  |
| North Africa and Middle East | 7.58 (69.84%) | 0.89 (8.16%) | 2.61 (24.05%) | -0.22 (-2.05%) | 10.86 (100.00%) |
| **South Asia** |  |  |  |  |  |
| South Asia | 11.64 (52.44%) | 3.02 (13.60%) | 7.91 (35.64%) | -0.37 (-1.68%) | 22.19 (100.00%) |
| **Sub-Saharan Africa** |  |  |  |  |  |
| Central Sub-Saharan Africa | 0.81 (93.29%) | -0.04 (-4.31%) | 0.1 (11.72%) | -0.01 (-0.70%) | 0.87 (100.00%) |
| Eastern Sub-Saharan Africa | 1.55 (64.44%) | 0.22 (9.12%) | 0.68 (28.00%) | -0.04 (-1.55%) | 2.41 (100.00%) |
| Southern Sub-Saharan Africa | 1.74 (88.50%) | -0.09 (-4.61%) | 0.34 (17.09%) | -0.02 (-0.97%) | 1.97 (100.00%) |
| Western Sub-Saharan Africa | 1.23 (87.67%) | -0.19 (-13.53%) | 0.38 (26.74%) | -0.01 (-0.88%) | 1.41 (100.00%) |

^a^ Percent in parentheses indicates the relative change of tracheal, bronchus, and lung cancer deaths associated with each decomposed factor compared with the total level. TBL cancer: tracheal, bronchus, and lung cancer; SDI: Socio-demographic index.

**Supplementary Table 9** Decomposition of change in age‐related deaths for TBL cancer in 5 SDI and 21 GBD regions between 1990 and 2021 in male.

| Location | Change in death, no, in thousands (%)^a^ | | | | |
| --- | --- | --- | --- | --- | --- |
|  | Population size | Population aging | Incidence | Case fatality | Total |
| **Global** | 616.67 (113.31%) | 274.55 (50.45%) | -240.41 (-44.18%) | -106.59 (-19.59%) | 544.22 (100.00%) |
| **SDI region** |  |  |  |  |  |
| High SDI | 128.99 (228.46%) | 123.5 (218.75%) | -146.99 (-260.34%) | -49.04 (-86.87%) | 56.46 (100.00%) |
| High-middle SDI | 162.59 (97.25%) | 116 (69.39%) | -62.45 (-37.35%) | -48.96 (-29.29%) | 167.18 (100.00%) |
| Middle SDI | 169.54 (66.28%) | 94.18 (36.82%) | 26.12 (10.21%) | -34.05 (-13.31%) | 255.79 (100.00%) |
| Low-middle SDI | 44.48 (81.68%) | 6.63 (12.18%) | 4.27 (7.84%) | -0.92 (-1.70%) | 54.45 (100.00%) |
| Low SDI | 13.12 (130.11%) | -1.27 (-12.60%) | -1.66 (-16.47%) | -0.11 (-1.04%) | 10.09 (100.00%) |
| **GBD regions** |  |  |  |  |  |
| **Southeast Asia, East Asia, and Oceania** |  |  |  |  |  |
| East Asia | 174.8 (48.42%) | 174.51 (48.34%) | 78.89 (21.85%) | -67.17 (-18.61%) | 361.02 (100.00%) |
| Southeast Asia | 43.01 (76.63%) | 14.69 (26.17%) | 0.29 (0.51%) | -1.86 (-3.31%) | 56.13 (100.00%) |
| Oceania | 0.52 (94.88%) | 0.04 (7.89%) | -0.01 (-2.22%) | 0.00 (-0.55%) | 0.54 (100.00%) |
| **Central Europe, Eastern Europe, and Central Asia** |  |  |  |  |  |
| Central Asia | 5.93 (279.12%) | 1.18 (55.49%) | -9.04 (-425.92%) | -0.18 (-8.69%) | -2.12 (100.00%) |
| Central Europe | 5.7 (92.20%) | 13.57 (219.55%) | -10.11 (-163.58%) | -2.98 (-48.17%) | 6.18 (100.00%) |
| Eastern Europe | 3.82 (15.14%) | 18.00 (71.36%) | -41.04 (-162.69%) | -6.01 (-23.81%) | -25.23 (100.00%) |
| **High-income** |  |  |  |  |  |
| High-income Asia Pacific | 16.65 (37.86%) | 40.65 (92.47%) | -2.30 (-5.22%) | -11.04 (-25.11%) | 43.96 (100.00%) |
| Australasia | 3.62 (250.11%) | 2.42 (166.99%) | -3.33 (-230.00%) | -1.26 (-87.11%) | 1.45 (100.00%) |
| Western Europe | 40.64 (1773.74%) | 51.39 (2242.98%) | -70.10 (-3059.80%) | -24.21 (-1056.92%) | -2.29 (100.00%) |
| Southern Latin America | 5.68 (1493.08%) | 1.60 (420.93%) | -6.35 (-1668.62%) | -0.55 (-145.39%) | 0.38 (100.00%) |
| High-income North America | 43.86 (812.83%) | 38.92 (721.30%) | -77.11 (-1428.91%) | -11.07 (-205.21%) | -5.40 (100.00%) |
| **Latin America and Caribbean** |  |  |  |  |  |
| Caribbean | 2.84 (105.14%) | 1.05 (38.91%) | -0.58 (-21.58%) | -0.61 (-22.47%) | 2.70 (100.00%) |
| Andean Latin America | 2.23 (137.40%) | 0.58 (35.77%) | -1.07 (-65.72%) | -0.12 (-7.44%) | 1.63 (100.00%) |
| Central Latin America | 8.62 (133.66%) | 3.63 (56.31%) | -5.36 (-83.16%) | -0.44 (-6.81%) | 6.45 (100.00%) |
| Tropical Latin America | 11.36 (98.98%) | 5.25 (45.72%) | -4.57 (-39.79%) | -0.56 (-4.92%) | 11.48 (100.00%) |
| **North Africa and Middle East** |  |  |  |  |  |
| North Africa and Middle East | 39.9 (122.96%) | 2.42 (7.47%) | -9.25 (-28.51%) | -0.62 (-1.92%) | 32.45 (100.00%) |
| **South Asia** |  |  |  |  |  |
| South Asia | 34.7 (80.89%) | 7.28 (16.97%) | 1.83 (4.26%) | -0.91 (-2.12%) | 42.89 (100.00%) |
| **Sub-Saharan Africa** |  |  |  |  |  |
| Central Sub-Saharan Africa | 2.68 (137.16%) | -0.37 (-18.71%) | -0.35 (-17.77%) | -0.01 (-0.68%) | 1.95 (100.00%) |
| Eastern Sub-Saharan Africa | 3.54 (92.57%) | 0.18 (4.79%) | 0.17 (4.36%) | -0.07 (-1.71%) | 3.83 (100.00%) |
| Southern Sub-Saharan Africa | 5.25 (176.72%) | -0.77 (-26.07%) | -1.46 (-49.12%) | -0.05 (-1.53%) | 2.97 (100.00%) |
| Western Sub-Saharan Africa | 3.27 (100.65%) | -0.56 (-17.11%) | 0.56 (17.25%) | -0.03 (-0.78%) | 3.25 (100.00%) |

^a^ Percent in parentheses indicates the relative change of tracheal, bronchus, and lung cancer deaths associated with each decomposed factor compared with the total level. TBL cancer: tracheal, bronchus, and lung cancer; SDI: Socio-demographic index.

**Supplementary Table 10** Decomposition of change in age‐related DALYs for TBL cancer in 5 SDI and 21 GBD regions between 1990 and 2021 in female.

| Location | Change in DALYs, in thousands (%)^a^ | | | | |
| --- | --- | --- | --- | --- | --- |
|  | Population size | Population aging | Prevalence | Case fatality disease severity | Total |
| **Global** | 6360.51 (80.20%) | 1786.60 (22.53%) | 3592.82 (45.30%) | -3809.21 (-48.03%) | 7930.72 (100.00%) |
| **SDI region** |  |  |  |  |  |
| High SDI | 1253.30 (74.05%) | 742.43 (43.86%) | 1487.68 (87.89%) | -1790.79 (-105.80%) | 1692.61 (100.00%) |
| High-middle SDI | 1383.95 (54.94%) | 630.59 (25.03%) | 2155.08 (85.55%) | -1650.51 (-65.52%) | 2519.11 (100.00%) |
| Middle SDI | 2057.12 (73.59%) | 823.21 (29.45%) | 1089.77 (38.98%) | -1174.55 (-42.01%) | 2795.55 (100.00%) |
| Low-middle SDI | 500.24 (65.30%) | 79.98 (10.44%) | 229.94 (30.01%) | -44.05 (-5.75%) | 766.11 (100.00%) |
| Low SDI | 125.61 (84.23%) | -4.44 (-2.98%) | 33.52 (22.48%) | -5.57 (-3.73%) | 149.12 (100.00%) |
| **GBD regions** |  |  |  |  |  |
| **Southeast Asia, East Asia, and Oceania** |  |  |  |  |  |
| East Asia | 2298.23 (59.81%) | 1556.95 (40.52%) | 2587.23 (67.33%) | -2599.75 (-67.66%) | 3842.65 (100.00%) |
| Southeast Asia | 482.69 (66.39%) | 166.38 (22.88%) | 154.66 (21.27%) | -76.68 (-10.55%) | 727.05 (100.00%) |
| Oceania | 5.57 (79.46%) | 0.19 (2.78%) | 1.46 (20.88%) | -0.22 (-3.11%) | 7.01 (100.00%) |
| **Central Europe, Eastern Europe, and Central Asia** | | | | | |
| Central Asia | 37.08 (586.98%) | 2.66 (42.12%) | -41.87 (-662.84%) | -4.19 (-66.26%) | -6.32 (100.00%) |
| Central Europe | 44.63 (14.34%) | 73.10 (23.49%) | 290.02 (93.18%) | -96.49 (-31.00%) | 311.26 (100.00%) |
| Eastern Europe | 14.63 (22.69%) | 43.61 (67.63%) | -27.95 (-43.34%) | -94.79 (-146.98%) | -64.49 (100.00%) |
| **High-income** |  |  |  |  |  |
| High-income Asia Pacific | 120.11 (52.51%) | 196.96 (86.12%) | 186.94 (81.74%) | -275.29 (-120.37%) | 228.72 (100.00%) |
| Australasia | 44.78 (83.86%) | 15.38 (28.80%) | 49.78 (93.23%) | -56.54 (-105.88%) | 53.40 (100.00%) |
| Western Europe | 309.64 (38.29%) | 183.2 (22.65%) | 1231.07 (152.23%) | -915.21 (-113.17%) | 808.70 (100.00%) |
| Southern Latin America | 47.12 (62.41%) | 9.48 (12.55%) | 36.14 (47.86%) | -17.23 (-22.82%) | 75.50 (100.00%) |
| High-income North America | 591.44 (159.44%) | 367.18 (98.98%) | 54.96 (14.82%) | -642.63 (-173.24%) | 370.95 (100.00%) |
| **Latin America and Caribbean** |  |  |  |  |  |
| Caribbean | 32.00 (63.87%) | 13.16 (26.27%) | 14.44 (28.83%) | -9.50 (-18.97%) | 50.10 (100.00%) |
| Andean Latin America | 36.68 (84.81%) | 8.49 (19.63%) | 4.46 (10.31%) | -6.38 (-14.74%) | 43.25 (100.00%) |
| Central Latin America | 133.43 (99.03%) | 50.40 (37.40%) | -12.95 (-9.61%) | -36.14 (-26.82%) | 134.75 (100.00%) |
| Tropical Latin America | 164.35 (55.92%) | 64.73 (22.02%) | 90.89 (30.92%) | -26.05 (-8.86%) | 293.92 (100.00%) |
| **North Africa and Middle East** |  |  |  |  |  |
| North Africa and Middle East | 207.92 (75.73%) | 20.59 (7.50%) | 62.96 (22.93%) | -16.91 (-6.16%) | 274.57 (100.00%) |
| **South Asia** |  |  |  |  |  |
| South Asia | 336.05 (55.74%) | 60.97 (10.11%) | 236.62 (39.25%) | -30.77 (-5.10%) | 602.87 (100.00%) |
| **Sub-Saharan Africa** |  |  |  |  |  |
| Central Sub-Saharan Africa | 23.68 (95.99%) | -1.29 (-5.25%) | 3.09 (12.53%) | -0.81 (-3.27%) | 24.67 (100.00%) |
| Eastern Sub-Saharan Africa | 40.99 (68.70%) | 6.05 (10.14%) | 14.18 (23.76%) | -1.55 (-2.60%) | 59.66 (100.00%) |
| Southern Sub-Saharan Africa | 48.93 (90.79%) | -2.39 (-4.44%) | 9.79 (18.16%) | -2.43 (-4.52%) | 53.89 (100.00%) |
| Western Sub-Saharan Africa | 33.59 (86.98%) | -2.99 (-7.75%) | 9.10 (23.57%) | -1.08 (-2.80%) | 38.61 (100.00%) |

^a^ Percent in parentheses indicates the relative change of tracheal, bronchus, and lung cancer deaths associated with each decomposed factor compared with the total level. TBL cancer: tracheal, bronchus, and lung cancer; SDI: Socio-demographic index; DALYs: disability-adjusted life-years.

**Supplementary Table 11** Decomposition of change in age‐related DALYs for TBL cancer in 5 SDI and 21 GBD regions between 1990 and 2021 in male.

| Location | Change in DALYs, in thousands (%)^a^ | | | | |
| --- | --- | --- | --- | --- | --- |
|  | Population size | Population aging | Prevalence | Case fatality disease severity | Total |
| **Global** | 15445.44 (151.58%) | 5444.63 (53.43%) | -2652.3 (-26.03%) | -8047.82 (-78.98%) | 10189.95 (100.00%) |
| **SDI region** |  |  |  |  |  |
| High SDI | 2902.62 (8717.88%) | 2213.53 (6648.22%) | -1405.05 (-4220.01%) | -3744.39 (-11246.08%) | -33.30 (100.00%) |
| High-middle SDI | 4216.88 (149.88%) | 2449.04 (87.05%) | -189.46 (-6.73%) | -3663.05 (-130.20%) | 2813.41 (100.00%) |
| Middle SDI | 4465.83 (78.42%) | 2023.91 (35.54%) | 1475.03 (25.90%) | -2269.84 (-39.86%) | 5694.94 (100.00%) |
| Low-middle SDI | 1230.18 (85.92%) | 139.07 (9.71%) | 144.82 (10.12%) | -82.32 (-5.75%) | 1431.75 (100.00%) |
| Low SDI | 369.54 (131.57%) | -34.64 (-12.33%) | -38.78 (-13.81%) | -15.26 (-5.43%) | 280.87 (100.00%) |
| **GBD regions** |  |  |  |  |  |
| **Southeast Asia, East Asia, and Oceania** |  |  |  |  |  |
| East Asia | 4564.64 (59.95%) | 3659.42 (48.06%) | 4147.25 (54.47%) | -4756.82 (-62.47%) | 7614.49 (100.00%) |
| Southeast Asia | 1158.79 (80.77%) | 369.30 (25.74%) | 52.20 (3.64%) | -145.63 (-10.15%) | 1434.66 (100.00%) |
| Oceania | 14.44 (96.59%) | 1.05 (7.02%) | -0.12 (-0.81%) | -0.42 (-2.80%) | 14.95 (100.00%) |
| **Central Europe, Eastern Europe, and Central Asia** | | | | | |
| Central Asia | 175.58 (202.75%) | 34.18 (39.47%) | -279.77 (-323.07%) | -16.58 (-19.15%) | -86.60 (100.00%) |
| Central Europe | 148.13 (215.60%) | 265.84 (386.91%) | -228.97 (-333.25%) | -253.71 (-369.26%) | -68.71 (100.00%) |
| Eastern Europe | 105.61 (11.22%) | 384.90 (40.88%) | -1002.61 (-106.49%) | -429.40 (-45.61%) | -941.50 (100.00%) |
| **High-income** |  |  |  |  |  |
| High-income Asia Pacific | 339.32 (61.95%) | 635.76 (116.07%) | 319.66 (58.36%) | -747.01 (-136.38%) | 547.73 (100.00%) |
| Australasia | 80.61 (731.57%) | 41.63 (377.85%) | -21.16 (-192.02%) | -90.06 (-817.39%) | 11.02 (100.00%) |
| Western Europe | 920.28 (154.72%) | 866.73 (145.72%) | -416.05 (69.95%) | -1965.76 (330.49%) | -594.80 (100.00%) |
| Southern Latin America | 143.36 (464.37%) | 29.34 (95.05%) | -163.95 (-531.07%) | -39.62 (-128.35%) | -30.87 (100.00%) |
| High-income North America | 988.95 (216.82%) | 799.69 (175.32%) | -1469.67 (-322.21%) | -775.09 (-169.93%) | -456.12 (100.00%) |
| **Latin America and Caribbean** |  |  |  |  |  |
| Caribbean | 68.01 (114.09%) | 22.39 (37.56%) | 3.3 (5.53%) | -34.09 (-57.19%) | 59.61 (100.00%) |
| Andean Latin America | 54.53 (162.60%) | 11.31 (33.73%) | -24.25 (-72.30%) | -8.06 (-24.04%) | 33.53 (100.00%) |
| Central Latin America | 211.56 (155.16%) | 76.29 (55.95%) | -121.66 (-89.23%) | -29.83 (-21.88%) | 136.35 (100.00%) |
| Tropical Latin America | 290.54 (122.30%) | 112.4 (47.31%) | -123.04 (-51.79%) | -42.34 (-17.82%) | 237.56 (100.00%) |
| **North Africa and Middle East** |  |  |  |  |  |
| North Africa and Middle East | 1084.88 (136.18%) | 63.43 (7.96%) | -269.72 (-33.86%) | -81.94 (-10.29%) | 796.65 (100.00%) |
| **South Asia** |  |  |  |  |  |
| South Asia | 983.77 (85.61%) | 148.80 (12.95%) | 92.30 (8.03%) | -75.76 (-6.59%) | 1149.11 (100.00%) |
| **Sub-Saharan Africa** |  |  |  |  |  |
| Central Sub-Saharan Africa | 77.88 (131.39%) | -6.79 (-11.45%) | -9.21 (-15.54%) | -2.61 (-4.40%) | 59.27 (100.00%) |
| Eastern Sub-Saharan Africa | 103.30 (97.37%) | 8.17 (7.70%) | -3.00 (-2.83%) | -2.37 (-2.24%) | 106.09 (100.00%) |
| Southern Sub-Saharan Africa | 149.51 (182.28%) | -17.75 (-21.65%) | -41.89 (-51.08%) | -7.84 (-9.56%) | 82.02 (100.00%) |
| Western Sub-Saharan Africa | 86.27 (100.91%) | -13.84 (-16.19%) | 15.45 (18.07%) | -2.39 (-2.79%) | 85.49 (100.00%) |

^a^ Percent in parentheses indicates the relative change of tracheal, bronchus, and lung cancer deaths associated with each decomposed factor compared with the total level. TBL cancer: tracheal, bronchus, and lung cancer; SDI: Socio-demographic index; DALYs: disability-adjusted life-years

**Supplementary Table 12** AAPC of concentration indices of tracheal, bronchus, and lung cancer across 21 GBD region between 1990 and 2021 by gender.

| Gender | CI of ASDR | | |  | CI of ASDAR | | |
| --- | --- | --- | --- | --- | --- | --- | --- |
|  | 1990 | 2021 | AAPC, (95%CI) |  | 1990 | 2021 | AAPC, (95%CI) |
| Female | 0.24 | 0.21 | -0.30 (-0.58, -0.03) * |  | 0.24 | 0.19 | -0.22 (-0.56, 0.12) |
| Male | 0.27 | 0.20 | -0.96 (-1.13, -0.78) * |  | 0.27 | 0.18 | -1.23 (-1.40, -1.06) * |

CI: concentration indices; ASDR: Age- standardized rate of Deaths; ASDAR: Age-standardized rate of DALYs; DALYs: disability-adjusted life-years; AAPC: average annual percentage change

**Supplementary Table 13**  Estimated economic burden of tracheal, bronchus, and lung cancer by SDI regions through 2050, billions of 2023 US$. (E=1.5)

| **Region** | **2021 economic burden 95%UI** | **2030 economic burden 95%UI** | **2040 economic burden 95%UI** | **2050 economic burden 95%UI** |
| --- | --- | --- | --- | --- |
| **World** |  |  |  |  |
| Both | 3585.39  (3128.23, 4020.32) | 4564.09  (3798.88, 5333.96) | 5601.69  (4460.56, 6778.44) | 6760.92  (5046.48, 8686.15) |
| Male | 2300.12  (1973.14, 2639.8) | 2838.65  (2312.77, 3384.04) | 3368.14  (2633.73, 4156.47) | 3915.55  (2882.13, 5077.54) |
| Female | 1285.26  (1093.32, 1459.22) | 1725.44  (1403.15, 2057.59) | 2233.55  (1730.93, 2787.27) | 2845.37  (2061.19, 3768.86) |
| **Socio-demographic index region** | |  |  |  |
| High SDI |  |  |  |  |
| Both | 2089.49  (1917.74, 2228.77) | 2445.6  (2123.24, 2748.91) | 2647.65  (2232, 3072.89) | 2798.62  (2272.68, 3374.1) |
| Male | 1257.25  (1162.56, 1344.76) | 1413.81  (1230.6, 1606) | 1463.39  (1238.9, 1718.95) | 1476.01  (1196.02, 1799.05) |
| Female | 832.24  (744.56, 895.03) | 1031.78  (880.33, 1169.67) | 1184.27  (974.84, 1394.72) | 1322.61  (1040.96, 1626.37) |
| High-middle SDI | |  |  |  |
| Both | 1136.29  (949.79, 1334.41) | 1614.74  (1316.37, 1937.44) | 2286.66  (1776, 2823.71) | 3118.99  (2230.11, 4140.03) |
| Male | 800.42  (636.49, 978.71) | 1094.88  (843.68, 1348.76) | 1481.71  (1102.01, 1871.48) | 1923.18  (1343.94, 2561.34) |
| Female | 335.87  (272.25, 405.06) | 519.86  (411.05, 645.83) | 804.94  (606.88, 1045.9) | 1195.81  (833.08, 1661.33) |
| Middle SDI | |  |  |  |
| Both | 331.34  (238.41, 422.47) | 461.33  (326.91, 593.31) | 604.69  (408.03, 797.65) | 753.3  (485.36, 1040.54) |
| Male | 222.21  (158.59, 290.73) | 300.89  (216.93, 391.59) | 382.15  (264.45, 509.99) | 460.56  (306.81, 634.48) |
| Female | 109.12  (70.23, 148.91) | 160.44  (101.87, 224.47) | 222.54  (134.15, 316.38) | 292.75  (165.42, 430.18) |
| Low-middle SDI | |  |  |  |
| Both | 27.34 (21.62, 33.38) | 41.02 (31.37, 52.32) | 60.31 (42.91, 80.75) | 85.57 (55.42, 124.9) |
| Male | 19.58 (15.06, 24.65) | 28.1 (20.91, 36.28) | 39.29 (27.31, 53.65) | 52.84 (33.49, 78.15) |
| Female | 7.76 (6.1, 9.82) | 12.92 (9.62, 16.98) | 21.02 (14.56, 29.09) | 32.73 (20.81, 48.71) |
| Low SDI | |  |  |  |
| Both | 0.93 (0.66, 1.3) | 1.4 (0.97, 1.99) | 2.38 (1.62, 3.43) | 4.43 (2.9, 6.58) |
| Male | 0.66 (0.45, 0.95) | 0.97 (0.65, 1.42) | 1.6 (1.06, 2.39) | 2.96 (1.88, 4.53) |
| Female | 0.27 (0.18, 0.39) | 0.43 (0.28, 0.64) | 0.77 (0.49, 1.17) | 1.48 (0.91, 2.28) |

SDI: Socio-demographic index. Note: Data were available for 169 countries covering more than 99% of the world population. Estimates were adjusted to 2023 constant US$ using consumer price index data from the U.S. Bureau of Labor Statistics. All future year estimates were discounted at a 3% annual discount rate.

**Supplementary Table 14** Estimated economic burden of tracheal, bronchus, and lung cancer by SDI regions through 2050, billions of 2023 US$. (E=1.0)

| **Region** | **2021 economic burden 95%UI** | **2030 economic**  **burden 95%UI** | | **2040 economic**  **burden 95%UI** | | **2050 economic burden 95%UI** |
| --- | --- | --- | --- | --- | --- | --- |
| **World** |  | |  | |  |  |
| Both | 5091.93  (4327.48, 5842.32) | | 6708.08  (5474.42, 7966.46) | | 8598.89  (6697.45, 10557.2) | 10793.23  (7848.34, 14111.58) |
| Male | 3334.17  (2764.38, 3938.21) | | 4262  (3385.55, 5172.07) | | 5281.25  (4025.22, 6619.12) | 6380.64  (4577.7, 8404.03) |
| Female | 1757.76  (1450.45, 2053.66) | | 2446.08  (1937.08, 2988.06) | | 3317.64  (2499.79, 4235.21) | 4412.59  (3097.73, 5981.97) |
| **Socio-demographic index region** | | |  | |  |  |
| High SDI |  | |  | |  |  |
| Both | 2279.05  (2088.4, 2433.94) | | 2663.37  (2308.84, 2998.76) | | 2888.49  (2431.36, 3357.39) | 3060.53  (2479.74, 3697.26) |
| Male | 1383.37  (1276.86, 1481.49) | | 1554.9  (1350.92, 1769.43) | | 1614.68  (1363.75, 1900.36) | 1635.5  (1321.24, 1999.01) |
| Female | 895.69  (799.69, 964.96) | | 1108.47  (943.36, 1258.96) | | 1273.82  (1046.12, 1503.17) | 1425.03  (1119.4, 1756.7) |
| High-middle SDI | | |  | |  |  |
| Both | 1958.53  (1620.83, 2320.7) | | 2840.55  (2307.2, 3418.25) | | 4103.1  (3176.12, 5073.66) | 5683.86  (4047.8, 7561.35) |
| Male | 1372.9  (1073.34, 1701.43) | | 1916.12  (1463.54, 2372.56) | | 2645.2  (1955.17, 3350.18) | 3487.84  (2424.48, 4654.37) |
| Female | 585.63  (470.83, 712.22) | | 924.43  (728.26, 1152.68) | | 1457.91  (1095.82, 1899.65) | 2196.03  (1524.7, 3058.52) |
| Middle SDI | | |  | |  |  |
| Both | 765.53  (548.43, 978.2) | | 1070.61  (756.93, 1377.59) | | 1408.64  (949.22, 1858.42) | 1760.07  (1133.76, 2430.34) |
| Male | 514.47  (365.79, 674.57) | | 699.66  (503.71, 910.9) | | 891.99  (616.88, 1190.31) | 1078.25  (718.65, 1484.26) |
| Female | 251.06  (160.21, 343.96) | | 370.95  (234.34, 520.33) | | 516.65  (310.22, 735.72) | 681.82  (384.15, 1002.71) |
| Low-middle SDI | | |  | |  |  |
| Both | 84.37 (66.66, 103.21) | | 126.64 (96.7, 161.9) | | 186.56 (132.57, 250.04) | 265.7 (172.04, 387.98) |
| Male | 60.27 (46.23, 76.11) | | 86.54 (64.2, 112.03) | | 121.2 (84.08, 165.79) | 163.6 (103.63, 242.27) |
| Female | 24.1 (18.87, 30.63) | | 40.09 (29.76, 52.91) | | 65.36 (45.17, 90.75) | 102.1 (64.81, 152.29) |
| Low SDI | | |  | |  |  |
| Both | 4.44 (3.16, 6.28) | | 6.91 (4.76, 9.96) | | 12.1 (8.17, 17.68) | 23.08 (14.99, 34.65) |
| Male | 3.15 (2.15, 4.62) | | 4.77 (3.18, 7.15) | | 8.19 (5.33, 12.49) | 15.47 (9.71, 24.12) |
| Female | 1.29 (0.85, 1.89) | | 2.14 (1.36, 3.17) | | 3.91 (2.46, 5.92) | 7.61 (4.67, 11.75) |

SDI: Socio-demographic index. Note: Data were available for 169 countries covering more than 99% of the world population. Estimates were adjusted to 2023 constant US$ using consumer price index data from the U.S. Bureau of Labor Statistics. All future year estimates were discounted at a 3% annual discount rate.

**Supplementary Table 15** Estimated economic burden of tracheal, bronchus, and lung cancer by country through 2050, millions of 2023 US$.

| Location | **2021**  **economic burden 95%UI** | | **2030**  **economic burden 95%UI** | **2040**  **economic burden 95%UI** | **2050**  **economic burden 95%UI** |
| --- | --- | --- | --- | --- | --- |
| Afghanistan | | 24.3 (15.71, 35.15) | 24.47 (13.44, 39.12) | 25.06 (12.89, 40.54) | 25.02 (11.62, 44.27) |
| Albania | | 622.82 (439.11, 840.67) | 831.14 (533.66, 1148.94) | 1064.33 (651.35, 1526.4) | 1306.65 (770.94, 1962.38) |
| Algeria | | 842.53 (653.15, 1089.95) | 839.44 (590.82, 1125.96) | 784.8 (530.35, 1094.59) | 681.27 (414.77, 1001.42) |
| Angola | | 208.42 (150.82, 278.34) | 208.42 (153.75, 276.81) | 206.46 (150.02, 286.04) | 204.02 (141.84, 293.3) |
| Argentina | | 12058.65 (10934.1, 13306.81) | 14370.72 (11961.1, 17187.95) | 16716.02 (13481.04, 20451.56) | 18817.49 (14730.47, 23730.89) |
| Armenia | | 688.76 (610.27, 779.92) | 935.48 (747.84, 1143.32) | 1221.85 (930.66, 1637.84) | 1545.95 (1038.21, 2275.56) |
| Australia | | 37275.83 (33839.9, 40511.04) | 44574 (38317.23, 50178.92) | 51246.24 (42783.18, 59326.52) | 58313.06 (46401.17, 70261.8) |
| Austria | | 20249.38 (18607.85, 21899.03) | 22119.31 (19203.73, 24904.35) | 22603.13 (19023.89, 26233.26) | 22473.21 (18294.83, 27249.13) |
| Azerbaijan | | 1221.7 (823.93, 1660.48) | 1273.94 (861.74, 1821.78) | 1210.05 (775.59, 1846.22) | 1082.67 (608.93, 1739.3) |
| Bahamas | | 168.21 (134.48, 214.1) | 190.67 (144.76, 244.02) | 196.43 (143.5, 262.23) | 188.96 (122.09, 267.16) |
| Bahrain | | 732.51 (587.2, 935.03) | 1042.42 (811.65, 1381.97) | 1380.57 (983.69, 1878.92) | 1661.63 (1075.45, 2497.83) |
| Bangladesh | | 1322.57 (967.81, 1833.54) | 2444.44 (1702.94, 3561.17) | 4517.84 (2966.12, 6717.81) | 8116.23 (5002.27, 12936.17) |
| Barbados | | 57.44 (43.75, 72.79) | 49.1 (36.64, 64.46) | 37.06 (26.2, 50.02) | 26.77 (16.67, 38.6) |
| Belarus | | 4606.65 (3559.11, 5666.86) | 4578.58 (3482.03, 5788.67) | 4256.88 (3012.35, 5768.8) | 3810.29 (2436.08, 5592.39) |
| Belgium | | 33698.04 (30584.84, 36600.03) | 38614.97 (33277.42, 44498.46) | 41557.47 (34821.08, 49348.86) | 43575.52 (35286.15, 54266.03) |
| Belize | | 9.62 (8.25, 11.14) | 12.42 (10, 15.12) | 14.95 (10.96, 19.73) | 17.25 (11.07, 24.99) |
| Benin | | 19.24 (14.1, 25.91) | 32.25 (23.25, 42.92) | 55.2 (39.34, 75.25) | 95.89 (65.58, 135.77) |
| Bhutan | | 13.05 (8.71, 18.84) | 23.2 (15.6, 33.19) | 42.15 (26.45, 63.94) | 72.67 (41.5, 118.1) |
| Bolivia (Plurinational State of) | | 262.13 (180.49, 364.68) | 422.09 (273.07, 590.53) | 736.84 (472.21, 1089.44) | 1247.14 (751.84, 1938.98) |
| Bosnia and Herzegovina | | 1933.37 (1511.27, 2420.1) | 2624.64 (1987.79, 3401.02) | 3482.37 (2522.73, 4669.45) | 4367.15 (2843.13, 6307.45) |
| Botswana | | 166.72 (120.62, 217.5) | 240.38 (167.21, 317.35) | 355.77 (249.89, 477.46) | 495.73 (332.36, 669.72) |
| Brazil | | 22068.9 (20854.47, 23222.3) | 22645.88 (18552.05, 27011.54) | 21647.34 (16330.03, 27976.07) | 19894.32 (13294.5, 28710.79) |
| Brunei Darussalam | | 476.24 (382.05, 574.14) | 471.39 (366.16, 602.45) | 403.73 (302.92, 537.36) | 312.55 (217.77, 433.98) |
| Bulgaria | | 8255.28 (6887.54, 9680.81) | 10164.15 (8126.62, 12646.07) | 12369.3 (9658.44, 15981.8) | 13906.3 (10092.64, 18998.23) |
| Burkina Faso | | 16.54 (11.9, 24.68) | 26.09 (18.59, 37.32) | 44.42 (31.24, 65.4) | 77.77 (53.25, 117.42) |
| Burundi | | 1.93 (1.35, 2.88) | 2.53 (1.68, 3.95) | 3.54 (2.31, 5.53) | 4.84 (3.04, 7.75) |
| Cabo Verde | | 12.07 (8.96, 15.05) | 16.04 (11.64, 20.89) | 21.1 (15.18, 27.79) | 26.66 (18.54, 36.44) |
| Cambodia | | 305.62 (217.63, 399.82) | 513.65 (353.13, 691.9) | 866.24 (589.72, 1195.78) | 1456.67 (936.71, 2112.21) |
| Cameroon | | 77.27 (52.33, 107.13) | 111.67 (74.81, 156.38) | 168.21 (117.26, 235.76) | 253.02 (171, 364.09) |
| Canada | | 82186.39 (75776.63, 88478.91) | 96003.34 (83909.49, 108371.73) | 101911.58 (86713.13, 119874.94) | 103882.5 (84019.02, 126234.14) |
| Central African Republic | | 2.67 (1.6, 5.02) | 2.62 (1.47, 5.07) | 2.57 (1.44, 4.92) | 2.46 (1.3, 4.8) |
| Chad | | 7.16 (5.07, 10.34) | 6.87 (4.71, 9.56) | 6.83 (4.71, 9.75) | 7.27 (4.73, 10.57) |
| Chile | | 6728.26 (6119.66, 7296.25) | 8587.97 (7341.07, 9807.7) | 10456.59 (8692.22, 12237.35) | 12195.52 (9765.93, 14640.62) |
| China | | 688025.62 (549130.25, 840441.18) | 1089356.66 (877828.18, 1317379.91) | 1699703.99 (1306521.75, 2099423.31) | 2489079.07 (1756365.75, 3322691.21) |
| Colombia | | 3407.42 (2835.41, 4140.57) | 4971.06 (3964.11, 6132.82) | 7073.47 (5265.38, 9149.35) | 9673.99 (6424.98, 13279.47) |
| Comoros | | 2.28 (1.5, 3.19) | 3.23 (2.07, 4.63) | 4.38 (2.69, 6.43) | 5.62 (3.31, 8.42) |
| Congo | | 28.41 (20.65, 38.43) | 22.5 (15.71, 31.09) | 16.48 (11.31, 23.34) | 11.28 (7.47, 16.61) |
| Costa Rica | | 348.46 (303.25, 394.9) | 554.32 (451.44, 669.88) | 824.24 (624.19, 1075.71) | 1172.94 (814.49, 1638.59) |
| Croatia | | 9671.48 (8407.16, 10925.21) | 13607.61 (11093.21, 16192.75) | 18222 (14510.58, 22211.24) | 23299.5 (17352.61, 29991.74) |
| Cyprus | | 1239.24 (1007.73, 1505.11) | 1486.73 (1172.65, 1897.28) | 1726.15 (1299.28, 2283.65) | 1975.63 (1421.93, 2713.44) |
| Czechia | | 22944.87 (20005.5, 25769.56) | 29411.54 (24522.43, 34980.93) | 38292.29 (30705.94, 46995.44) | 47232.52 (36339.73, 60458.3) |
| Democratic Republic of the Congo | | 35.25 (21.48, 61.84) | 72.98 (37.7, 141.29) | 152.13 (78.56, 289.96) | 310.63 (158.47, 612.01) |
| Denmark | | 21604.22 (19680.98, 23535.3) | 27102.85 (23158.85, 31183.69) | 31660.1 (26433.33, 36816.08) | 36928.93 (29761.2, 43859.38) |
| Djibouti | | 9.01 (5.83, 13.25) | 16.93 (10.62, 25.8) | 31 (19.32, 46.53) | 52.78 (32.23, 79.01) |
| Dominican Republic | | 1132.17 (765.77, 1539.5) | 2079.74 (1323.9, 2879.89) | 3837.97 (2319.44, 5479.36) | 6842.85 (3812.63, 10378.34) |
| Ecuador | | 409.36 (318.89, 520.49) | 501.48 (368.93, 656.29) | 591.7 (419.53, 807.44) | 675.08 (450.19, 971.45) |
| Egypt | | 4143.55 (3219.66, 5186.69) | 5866.4 (4517.67, 7385.71) | 8029.29 (5815.98, 10784.22) | 10257.7 (6749.71, 15039.17) |
| El Salvador | | 147.22 (117.23, 185.51) | 221.92 (162.12, 303.62) | 327.66 (229.71, 466.63) | 465.27 (299.53, 700.03) |
| Equatorial Guinea | | 33.23 (21.17, 48.37) | 23.97 (15.74, 35.05) | 17.7 (11.38, 25.8) | 12.64 (7.74, 18.89) |
| Estonia | | 2341.67 (1978.41, 2655.36) | 3076.32 (2498.63, 3719.02) | 4040.83 (3099.29, 5148.65) | 5302.66 (3833.62, 7198.85) |
| Ethiopia | | 114.64 (91.29, 143.13) | 282.15 (212.06, 369.61) | 749.81 (546.75, 991.96) | 1969.74 (1365.69, 2665.9) |
| Fiji | | 27.93 (21.1, 36.42) | 38.35 (28.07, 49.4) | 49.56 (34.79, 64.71) | 61.41 (40.43, 86.52) |
| Finland | | 10661.51 (9630.09, 11653.59) | 11719.75 (10018.9, 13423.33) | 12080.98 (10115.8, 14356.06) | 12429.38 (9923.64, 15141.97) |
| France | | 163446.59 (149071.53, 177668.29) | 189857.78 (163086.16, 219572.91) | 205934.2 (171643.97, 240717.3) | 215658.28 (176105.46, 258028.88) |
| Gabon | | 78.13 (54.83, 104.31) | 81.71 (55.31, 111.01) | 81.92 (55.1, 112.04) | 82.21 (54.03, 116.49) |
| Gambia | | 0.94 (0.66, 1.28) | 1.09 (0.74, 1.5) | 1.29 (0.86, 1.8) | 1.56 (0.99, 2.24) |
| Georgia | | 1122.74 (963.64, 1295.71) | 1929.92 (1557.25, 2377.45) | 2874.89 (2208.23, 3744.69) | 3903.8 (2788.4, 5455.04) |
| Germany | | 247803.69 (227658.05, 267698.94) | 280437.81 (241945.33, 317866.78) | 287019.28 (241227.58, 330111.71) | 288502.76 (238902.27, 338135.12) |
| Ghana | | 125.31 (91.41, 159.03) | 171.62 (127.76, 226.15) | 239.75 (174.43, 319.32) | 332.04 (231.08, 454.28) |
| Greece | | 20584.65 (19120.16, 22003) | 20963.86 (17584.23, 24394.33) | 20000.75 (16506.9, 24050.89) | 17566.41 (13774.89, 21663.01) |
| Grenada | | 7.21 (6.07, 8.37) | 9.84 (7.74, 12.2) | 12.39 (9.12, 16.51) | 15.02 (9.8, 21.97) |
| Guatemala | | 166.13 (139.83, 194) | 224.98 (182.57, 274.08) | 308.53 (232.69, 394.33) | 418.04 (284.79, 575.3) |
| Guinea | | 16.82 (11.77, 23.82) | 25.06 (18.01, 34.92) | 40.12 (28.24, 56.66) | 65.16 (43.18, 94.23) |
| Guinea-Bissau | | 1.86 (1.36, 2.48) | 3.12 (1.8, 4.31) | 5.84 (3.86, 8.36) | 10.79 (7.11, 15.98) |
| Guyana | | 111.98 (82.87, 145.31) | 253.82 (186.77, 337.79) | 526.68 (365.91, 726.19) | 1051.49 (665.26, 1569.7) |
| Haiti | | 47.9 (29.16, 75.61) | 54.94 (35.04, 84.86) | 62.67 (37.71, 101.36) | 69.15 (37.06, 119.34) |
| Honduras | | 149.34 (103.52, 213.02) | 240.22 (155.14, 367.82) | 401.48 (243.17, 639.41) | 667.82 (378.67, 1141.25) |
| Hungary | | 30240.96 (25864.95, 34709.1) | 38427.31 (30440.91, 46500.17) | 47588.96 (36694.45, 61216.04) | 55151.31 (40090, 72609.52) |
| Iceland | | 588.45 (524.93, 652.68) | 797.94 (676.03, 925.43) | 1062.13 (868.43, 1256.15) | 1390.96 (1066.17, 1700.65) |
| India | | 15352.24 (12630.44, 17777.42) | 23604.12 (18587, 29084.03) | 34959.23 (25321.01, 45282.51) | 49164.59 (32081.59, 69734.98) |
| Indonesia | | 243155.61 (164994.52, 318581) | 352118.47 (242067.41, 456381.29) | 472545.4 (310414.63, 625787.41) | 594930.25 (376232.5, 821989.82) |
| Iran (Islamic Republic of) | | 5107.41 (4644.57, 5614.32) | 5808.47 (4816.5, 6906.35) | 6191.55 (4630.86, 7863.72) | 6061.85 (4039.67, 8393.79) |
| Iraq | | 1011.93 (735.48, 1300.47) | 830.71 (496.74, 1182.74) | 641.6 (367.25, 975.62) | 457.81 (247.38, 753.91) |
| Ireland | | 11119.79 (9963.05, 12289.78) | 21251.11 (17798.76, 24388.25) | 41192.49 (34309.96, 48123.9) | 77228.4 (63024.55, 93114.91) |
| Israel | | 5805.62 (5248.37, 6341.19) | 7182.7 (5782.99, 8533.62) | 8659.46 (6886.45, 10680.27) | 10102.66 (7709.54, 12956.19) |
| Italy | | 137977.91 (127318.02, 145710.92) | 152860.08 (132848.24, 172151.75) | 159023.33 (133687.54, 184529.05) | 154037.21 (125039.68, 185728.04) |
| Jamaica | | 144.83 (110.18, 189.72) | 175.29 (127.81, 233.17) | 199.05 (136.55, 276.17) | 217.4 (136.15, 322.02) |
| Japan | | 261982.55 (233713.61, 277261.84) | 249150.57 (208763.25, 280904.58) | 216365.2 (176445.92, 250577.18) | 183002.62 (144351.78, 222423.93) |
| Jordan | | 222.96 (169.18, 296.84) | 272.45 (201.52, 373.19) | 312.29 (217.82, 447.59) | 334.55 (215.71, 498.35) |
| Kazakhstan | | 3795.27 (3260.14, 4406.35) | 4630.48 (3713.32, 5661.18) | 5349.65 (3947.14, 7039.08) | 5851.62 (3870.58, 8420.36) |
| Kenya | | 85.68 (66.91, 109.28) | 187.38 (147.09, 246.63) | 435.98 (325.99, 585.94) | 992.02 (696.41, 1395.09) |
| Kiribati | | 1.09 (0.79, 1.48) | 1.61 (1.19, 2.18) | 2.29 (1.65, 3.09) | 3.23 (2.17, 4.63) |
| Kuwait | | 1123.9 (913.87, 1381.38) | 1174.97 (890.72, 1508.11) | 1119.99 (800.74, 1499.54) | 918.62 (589.05, 1314.06) |
| Lao People's Democratic Republic | | 220.49 (159.6, 307.2) | 435.17 (316.03, 604.92) | 866.26 (620.3, 1224.55) | 1687.32 (1147.02, 2516.69) |
| Latvia | | 3011.2 (2545.49, 3440.34) | 3778.98 (3117.27, 4518.21) | 4581.57 (3590.73, 5728.59) | 5367.54 (3913.83, 7036.98) |
| Lebanon | | 548.03 (431.1, 694.7) | 460.21 (345.62, 614.98) | 409.23 (296.8, 576.49) | 346.83 (230.96, 510.23) |
| Lesotho | | 18.33 (12.8, 25.86) | 17.94 (12.26, 24.84) | 19.91 (13.32, 28.09) | 22.87 (14.91, 33.45) |
| Liberia | | 1.83 (1.15, 2.9) | 3.11 (1.9, 4.86) | 5.3 (3.22, 8.38) | 8.35 (4.92, 13.52) |
| Libya | | 1266.9 (900.13, 1758.35) | 1355.97 (930.27, 2025.48) | 1303.24 (862.92, 1971.58) | 1041.97 (630.44, 1610.4) |
| Lithuania | | 5419.03 (4592.38, 6244.25) | 8167.55 (6819.71, 9599.02) | 10971.08 (8683.92, 13664.51) | 14069.4 (10094.38, 18748.43) |
| Luxembourg | | 1830.87 (1645.14, 2027.73) | 2231.12 (1912.47, 2563.34) | 2481.2 (2071.08, 2906.69) | 2615.98 (2113.68, 3112.85) |
| Madagascar | | 12.5 (9.04, 16.73) | 15.36 (11.03, 20.48) | 18.68 (13.13, 25.26) | 21.94 (15.11, 31.1) |
| Malawi | | 3.85 (2.86, 5.16) | 4.5 (3.27, 6.13) | 5.57 (3.87, 7.82) | 6.83 (4.46, 9.75) |
| Malaysia | | 7180.19 (6046.95, 8330.64) | 9941.08 (8038.53, 12334.33) | 13230.34 (10262.56, 17037.63) | 16966.28 (12204.66, 23195.92) |
| Maldives | | 16.79 (12.97, 20.8) | 36.11 (26.52, 46.16) | 84.51 (62.11, 110.31) | 185.52 (131.79, 252.21) |
| Mali | | 11.69 (8.68, 15.63) | 17.86 (12.9, 24.36) | 28.36 (19.51, 39.5) | 46.61 (31.26, 66.96) |
| Malta | | 591.92 (525.15, 666.36) | 823.19 (670.13, 987.43) | 1113.29 (880.18, 1366.83) | 1470.99 (1104.86, 1857.47) |
| Mauritania | | 16.26 (11.73, 22.97) | 22.72 (16.28, 31.18) | 33.06 (22.96, 45.36) | 48.81 (32.49, 68.09) |
| Mauritius | | 255.21 (233.59, 275.59) | 337.03 (285.17, 395.23) | 398.19 (322.64, 484.44) | 447.15 (343.03, 566.89) |
| Mexico | | 6939.6 (6128.43, 7778.02) | 8088.58 (6892.64, 9441.72) | 8728.87 (6886.46, 10765.49) | 8837.44 (6365.9, 11829.66) |
| Micronesia (Federated States of) | | 2.11 (1.45, 2.9) | 2.51 (1.74, 3.52) | 2.68 (1.82, 3.79) | 2.79 (1.73, 4.13) |
| Mongolia | | 222.78 (173.18, 285.1) | 288.94 (217.4, 368.93) | 343.54 (239.87, 458.02) | 376.32 (229.63, 559.32) |
| Montenegro | | 611.43 (481.85, 756.1) | 871.86 (645.65, 1120.57) | 1186.56 (870, 1576.04) | 1550.63 (1053.76, 2150.99) |
| Morocco | | 1192.11 (867.33, 1515.16) | 1494.55 (1086.42, 1974.44) | 1750.36 (1183.21, 2411.05) | 1913.22 (1162.89, 2944.66) |
| Mozambique | | 10.01 (7.31, 12.92) | 12.89 (9.61, 17.04) | 17.55 (12.42, 23.8) | 23.79 (16.1, 33.41) |
| Myanmar | | 858.38 (628.32, 1131.85) | 1011.02 (737.77, 1332) | 1112.09 (799.95, 1495.23) | 1186.95 (814.84, 1695.05) |
| Namibia | | 28.12 (20.28, 37.55) | 30.01 (21.88, 40.18) | 32.83 (23.94, 44.89) | 35.12 (25.07, 49.38) |
| Nepal | | 109.44 (80.77, 152.52) | 182.54 (127.6, 257.45) | 304.52 (208.51, 451) | 500.88 (320.88, 800.86) |
| Netherlands | | 58496.5 (53390.69, 62682.23) | 69426.81 (59544.99, 79569.51) | 74060.12 (62030.75, 86536.86) | 76077.06 (62067.96, 91085.63) |
| New Zealand | | 6645.79 (6100.56, 7234.61) | 9169.82 (7873.31, 10606.65) | 12039.97 (10052.22, 14255.34) | 15545.42 (12411.46, 19187.66) |
| Nicaragua | | 29.26 (23.4, 36.37) | 47.84 (35.71, 63.66) | 74 (52.73, 102.23) | 107.26 (71.85, 156.2) |
| Niger | | 4.29 (2.9, 7.34) | 5.36 (3.6, 9.05) | 6.7 (4.32, 11.13) | 9.21 (5.65, 15.46) |
| Nigeria | | 138.16 (106.74, 178.33) | 180.1 (133.9, 234.97) | 233.67 (172.56, 309.09) | 299.49 (210.36, 412.1) |
| North Macedonia | | 971.49 (736.01, 1219.42) | 1315.9 (908.45, 1794.64) | 1776.08 (1177.55, 2482.7) | 2222.66 (1353.61, 3295.44) |
| Norway | | 13611.58 (12486.82, 14367.04) | 15209.17 (13397.31, 17043.02) | 15799.71 (13580.23, 17858.5) | 16046.12 (13554.89, 18334.06) |
| Oman | | 257.07 (189.35, 354.14) | 293.98 (210.34, 408.89) | 307.58 (206.97, 441.06) | 280.02 (166.7, 426.3) |
| Pakistan | | 2123.32 (1590.72, 2790.2) | 3032.41 (2212.07, 4089.24) | 4272.77 (2913.08, 6029.18) | 5761.61 (3666, 8853.69) |
| Panama | | 732.36 (563.2, 891.46) | 1359.6 (1027.04, 1729.65) | 2498.07 (1789.94, 3297.39) | 4388.73 (2953.63, 6322.22) |
| Papua New Guinea | | 69.54 (45.29, 109.25) | 107.57 (67.76, 164.27) | 162.01 (99.05, 254.55) | 233.75 (133.09, 392.39) |
| Paraguay | | 443.49 (334.68, 587.14) | 657.85 (464.49, 921.72) | 922.01 (593.93, 1298.33) | 1225.54 (706.55, 1849.92) |
| Peru | | 1332.11 (962.3, 1704.01) | 1708.53 (1239.15, 2278.36) | 2205.26 (1544.19, 2988.41) | 2736.97 (1838.28, 3942.66) |
| Philippines | | 3700.12 (3029.69, 4431.68) | 4997.93 (3967.32, 6078.61) | 6495.69 (5117.98, 8041.15) | 8120.76 (6105.17, 10360.93) |
| Poland | | 100487.35 (91362.22, 109247.48) | 138363.87 (117594.97, 160118.77) | 191250.19 (158112.09, 227544.2) | 254813.9 (198021.63, 318495.53) |
| Portugal | | 13916.63 (12724.93, 15094.83) | 16428.23 (13683.63, 19231.07) | 18419.57 (14956.69, 22169.33) | 20122.01 (15899.71, 25006.57) |
| Qatar | | 1112.45 (811.92, 1583.37) | 1533.3 (1087.52, 2137.29) | 1662.09 (1138.82, 2380.82) | 1528.2 (987.04, 2288.29) |
| Republic of Korea | | 74589.16 (62812.1, 87369.32) | 99433.62 (83219.51, 121241.39) | 128711.09 (103632.58, 160689.91) | 149255.63 (112739.98, 193089.9) |
| Republic of Moldova | | 814.84 (714.92, 926.47) | 1341.91 (1022.9, 1680.36) | 2174.51 (1515.46, 2921.76) | 3206.7 (1984.17, 4628.93) |
| Romania | | 41938.05 (36737.25, 47532.85) | 60901.54 (48967.55, 75044.94) | 84625.88 (63769.31, 110002.89) | 107927.34 (76298.7, 148374.55) |
| Russian Federation | | 121018.18 (109130.19, 132014.7) | 139290.48 (117211.48, 164580.09) | 149812.38 (120175.22, 185959.69) | 158627.53 (116455.15, 208084.19) |
| Rwanda | | 16.04 (10.7, 22.5) | 31.33 (20.56, 45.95) | 62.51 (40.33, 90.25) | 124.3 (77.32, 183.37) |
| Saint Lucia | | 13.32 (10.71, 16.28) | 13.99 (10.46, 18.32) | 13.61 (9.39, 18.46) | 12.54 (7.65, 18.76) |
| Saint Vincent and the Grenadines | | 7.89 (6.91, 9.05) | 10.05 (8.12, 12.51) | 11.76 (8.55, 15.55) | 13.08 (8.32, 19.04) |
| Samoa | | 1.75 (1.27, 2.3) | 1.95 (1.41, 2.66) | 2.01 (1.43, 2.82) | 2.04 (1.38, 2.99) |
| Sao Tome and Principe | | 1.17 (0.87, 1.6) | 1.91 (1.37, 2.62) | 3.1 (2.23, 4.34) | 4.75 (3.24, 6.88) |
| Saudi Arabia | | 6107.11 (4675.53, 7718.79) | 8908.27 (6453.24, 12173.97) | 10611.38 (7354.62, 14808.97) | 10602.24 (6771.39, 16097.32) |
| Senegal | | 37.9 (28.08, 51.1) | 55.72 (41.5, 74.25) | 84.6 (62.5, 112.7) | 129.76 (92.56, 175.3) |
| Serbia | | 7867.18 (6195.41, 9723.18) | 9692.24 (7225.67, 12285.11) | 11913.21 (8608.86, 16080.19) | 14270.56 (9796.71, 19767.87) |
| Seychelles | | 41.7 (34.89, 49.34) | 59.38 (47.98, 73.22) | 77.54 (61.08, 97.57) | 92.44 (68.67, 120.49) |
| Sierra Leone | | 4.96 (3.44, 6.94) | 7.86 (5.35, 10.99) | 12.96 (8.81, 18.53) | 21.16 (13.71, 32.15) |
| Singapore | | 9969.95 (8956.39, 11174.48) | 13670.41 (11686.93, 15936.15) | 17559.92 (14261.63, 20970.15) | 20735.37 (15971.26, 25749.11) |
| Slovakia | | 7209.93 (5618.11, 8788.6) | 7730.13 (5934.24, 9819.92) | 7844.69 (5795.69, 10251.96) | 7448.76 (5281.1, 9898.74) |
| Slovenia | | 3423.91 (2897.38, 3941.8) | 4378.48 (3572.05, 5212.77) | 5345.22 (4203.06, 6728.81) | 6220.34 (4680.76, 8150.76) |
| Solomon Islands | | 3.59 (2.64, 4.93) | 4.63 (3.39, 6.16) | 5.72 (4.08, 7.81) | 6.8 (4.56, 9.85) |
| Somalia | | 7.55 (4.57, 14.51) | 14.14 (7.56, 29.32) | 28.34 (14.48, 59.07) | 53.92 (24.24, 119.83) |
| South Africa | | 5905.45 (5305.14, 6597.48) | 6264.33 (5310.87, 7340.22) | 6469.05 (5441.96, 7735.64) | 6267.33 (5006.37, 7688.53) |
| Spain | | 77003.46 (70789.39, 82890.14) | 84872.55 (73498.24, 96674.45) | 86186.54 (72335.57, 101338.1) | 82430.52 (67599.65, 100977.32) |
| Sri Lanka | | 1165.73 (745.96, 1618.42) | 1530.05 (1019.02, 2108.58) | 1850 (1229.9, 2553.46) | 2123.27 (1342.12, 3012.54) |
| Sudan | | 118.35 (78.22, 175.81) | 136.31 (89.21, 198.74) | 158.58 (103.11, 238.98) | 178.75 (103.71, 287.12) |
| Suriname | | 47.11 (35.42, 61.66) | 45.92 (33.18, 61.24) | 40.68 (27.32, 56.76) | 34.39 (20.8, 51.74) |
| Sweden | | 15277.57 (13256.92, 17270.72) | 17843.22 (14777.17, 20925.58) | 19736.28 (16226.93, 23608.98) | 21966.54 (17391.65, 26994.33) |
| Switzerland | | 20302.96 (18250.25, 21779.86) | 22289.03 (19012.25, 25548.4) | 22698.7 (18965.74, 26212.62) | 22124 (18121.4, 25822.28) |
| Tajikistan | | 62.14 (41.9, 90.19) | 100.16 (64.43, 148.3) | 152.61 (92.47, 243.66) | 225.25 (128.3, 388.3) |
| Thailand | | 20205.43 (15365.62, 25320.5) | 24240.53 (18126.92, 32019.34) | 26355.2 (19181.45, 34641.92) | 26426.77 (18546.03, 35925.85) |
| Timor-Leste | | 15.33 (11.32, 20.12) | 12.44 (9.26, 16.51) | 9.33 (6.75, 12.63) | 7.36 (4.9, 10.27) |
| Togo | | 10.06 (6.34, 14) | 15.31 (9.86, 21.53) | 22.79 (14.63, 33.05) | 32.68 (20.33, 48.96) |
| Tonga | | 4.05 (3.09, 5.18) | 5.23 (3.94, 6.74) | 6.63 (4.84, 8.79) | 8.18 (5.5, 11.35) |
| Tunisia | | 882.89 (587.27, 1251.24) | 965.58 (657.96, 1376.27) | 978.44 (654.62, 1414.29) | 904.78 (557.45, 1377.46) |
| Uganda | | 37.7 (27.18, 50.64) | 52.74 (37.11, 71) | 75.54 (51.48, 103.21) | 108.33 (71.2, 152.6) |
| Ukraine | | 9618.44 (6682.56, 13106.09) | 10988.12 (7598.39, 15085.92) | 11957.98 (7758.47, 17413.09) | 12107.89 (7292.36, 18800.95) |
| United Arab Emirates | | 3326.68 (2459.06, 5220.4) | 7222.99 (5123.59, 10943.75) | 11461.62 (7774.39, 17109.86) | 14621.54 (9368.82, 22577.98) |
| United Kingdom | | 125727.62 (117916.76, 130360.42) | 147829.11 (131819.4, 161807.12) | 164459.63 (142970.61, 186815.45) | 178303.66 (147993.88, 213580.76) |
| United Republic of Tanzania | | 75.14 (55.37, 102.31) | 114.45 (80.2, 155.78) | 170.59 (118.13, 236.61) | 251.92 (169.74, 359.93) |
| United States of America | | 903283.29 (844856.87, 942827.12) | 1068186.66 (945656.13, 1169220.02) | 1127918.43 (969453.17, 1284772.82) | 1142634.98 (947631.96, 1352634.92) |
| Uruguay | | 3095.17 (2857.62, 3319.29) | 3339.84 (2760.33, 3893.1) | 3512.96 (2856.6, 4181.53) | 3674.01 (2921.67, 4513.11) |
| Uzbekistan | | 542.63 (441.74, 654.22) | 796.33 (547.44, 1078.73) | 1089.22 (692.03, 1597.16) | 1426.3 (810.88, 2274.31) |
| Vanuatu | | 2.03 (1.33, 3.2) | 2.8 (1.89, 4.44) | 3.85 (2.56, 6.16) | 5.23 (3.19, 8.86) |
| Viet Nam | | 9109.93 (6712.53, 11449.32) | 16969.26 (12640.82, 21367.2) | 29145.37 (21425.89, 37627.93) | 46278.11 (32849.28, 62532.47) |
| Zambia | | 54.07 (36.76, 96.04) | 65.68 (45.53, 119.84) | 82.23 (57.57, 139.41) | 100.89 (68.92, 163.9) |
| Zimbabwe | | 35.57 (26.98, 47.28) | 39.13 (28.73, 51.84) | 45.33 (33.66, 61.58) | 51.79 (36.75, 72.92) |

Note: Data were available for 169 countries covering more than 99% of the world population. All future year estimates were discounted at a 3% annual discount rate.

**Supplementary Table 16** Estimated economic burden of tracheal, bronchus, and lung cancer by country through 2050 by sex, millions of 2023 US$.

| Location | economic burden 2021 95%UI | |  | economic burden 2050 95%UI | |
| --- | --- | --- | --- | --- | --- |
|  | Female | Male |  | Female | Male |
| Afghanistan | 10.48 (5.82, 15.67) | 13.81 (7.94, 26.03) |  | 11.31 (5.07, 20.49) | 13.71 (5.56, 28.86) |
| Albania | 148.86 (104.39, 203.41) | 473.96 (329.86, 641.3) |  | 395.87 (231.52, 604.86) | 910.78 (539.38, 1373.2) |
| Algeria | 158.9 (109.29, 215.32) | 683.63 (514.56, 902.97) |  | 163.06 (98.24, 249.73) | 518.21 (306.14, 770.43) |
| Angola | 51.79 (34.04, 75.95) | 156.63 (110.32, 209.53) |  | 62.54 (39.05, 97.49) | 141.48 (96.32, 204.87) |
| Argentina | 4102.44 (3640.88, 4570.64) | 7956.21 (7180.18, 8753.53) |  | 7428.38 (5679.14, 9661.44) | 11389.1 (8841.94, 14598.61) |
| Armenia | 115.04 (99.78, 131.83) | 573.73 (505.16, 652.44) |  | 340.72 (232.96, 490.53) | 1205.23 (798.02, 1764.35) |
| Australia | 15102.34 (13457.89, 16426.86) | 22173.5 (20208.78, 24337.92) |  | 29172.75 (22768.53, 36190.23) | 29140.31 (22877.98, 35408.55) |
| Austria | 8220.86 (7388.39, 8971.02) | 12028.52 (11064.06, 13214.58) |  | 10184.3 (8226.23, 12646.01) | 12288.91 (9755.11, 15091.47) |
| Azerbaijan | 243.38 (177.34, 328.16) | 978.32 (608.55, 1342.74) |  | 287.69 (181.76, 437.98) | 794.98 (412.87, 1360.81) |
| Bahamas | 52.54 (42.18, 65.74) | 115.67 (91.84, 148.07) |  | 75.05 (50.19, 104.41) | 113.91 (70.65, 165.22) |
| Bahrain | 189.93 (146.08, 240.71) | 542.58 (408.12, 725.52) |  | 360.49 (231.78, 560.42) | 1301.14 (793.51, 1964.76) |
| Bangladesh | 375.59 (241.16, 569.29) | 946.98 (677.24, 1401.86) |  | 3167.33 (1837.41, 5306.05) | 4948.89 (2958.47, 7978.01) |
| Barbados | 20.85 (16.13, 26.43) | 36.59 (27.49, 46.61) |  | 13.01 (8.32, 18.59) | 13.76 (8.15, 20.63) |
| Belarus | 579.77 (452.8, 710.89) | 4026.87 (3103.45, 4966.24) |  | 628.88 (406.54, 895.06) | 3181.41 (2036.57, 4721.13) |
| Belgium | 10865.08 (9757.52, 11793.88) | 22832.97 (20647.5, 25092.5) |  | 16751.53 (13348.5, 20777.94) | 26823.99 (21192.44, 34003.4) |
| Belize | 3.04 (2.62, 3.5) | 6.58 (5.54, 7.68) |  | 7.14 (4.89, 10.06) | 10.11 (6.14, 15.21) |
| Benin | 5.3 (3.56, 7.66) | 13.94 (10.39, 18.67) |  | 37.13 (24.33, 56.09) | 58.76 (39.76, 80.74) |
| Bhutan | 4.52 (2.63, 7.01) | 8.53 (5.28, 12.6) |  | 28.24 (14.72, 48.37) | 44.43 (24.42, 76.33) |
| Bolivia (Plurinational State of) | 114.98 (66.4, 170.98) | 147.15 (103.31, 205.33) |  | 606.86 (312.17, 990.63) | 640.28 (390.19, 1033.24) |
| Bosnia and Herzegovina | 458.65 (358.18, 582.43) | 1474.72 (1129.26, 1879.65) |  | 1347.23 (865.37, 1959.54) | 3019.93 (1956.98, 4432.76) |
| Botswana | 49.01 (34.27, 67.24) | 117.71 (77.27, 158.14) |  | 181.34 (119.22, 264.06) | 314.39 (198.32, 437.16) |
| Brazil | 9621.13 (8943.92, 10204.27) | 12447.78 (11702.56, 13111.31) |  | 9695.59 (6653.86, 13440.36) | 10198.73 (6566.3, 15257.44) |
| Brunei Darussalam | 213.02 (169.06, 261.83) | 263.22 (206.6, 328.87) |  | 158.49 (111.22, 220.3) | 154.07 (108.62, 215.37) |
| Bulgaria | 2013.27 (1657.24, 2393.6) | 6242.02 (5213.14, 7299.12) |  | 4357.26 (3154.88, 5826.61) | 9549.04 (6730.38, 13247.82) |
| Burkina Faso | 4.11 (2.6, 6.15) | 12.43 (9.05, 18.8) |  | 26.97 (16.16, 43.17) | 50.8 (34.27, 74.35) |
| Burundi | 0.45 (0.28, 0.67) | 1.49 (1.03, 2.26) |  | 1.59 (0.95, 2.59) | 3.26 (2.05, 5.2) |
| Cabo Verde | 3.7 (2.76, 4.99) | 8.37 (6.19, 10.5) |  | 9.66 (6.55, 13.72) | 17.01 (11.45, 23.07) |
| Cambodia | 85.76 (59.16, 117.52) | 219.86 (154.35, 298.16) |  | 475.52 (302.45, 715.78) | 981.15 (619.99, 1415.51) |
| Cameroon | 20.89 (13.53, 30.15) | 56.38 (37.24, 80.02) |  | 92.02 (57.36, 140.49) | 161 (106.65, 230.38) |
| Canada | 38866.51 (35180.04, 42267.52) | 43319.88 (39830.01, 47054.1) |  | 58680.44 (47576.29, 72941.7) | 45202.06 (36608.5, 54681.04) |
| Central African Republic | 0.51 (0.36, 0.71) | 2.17 (1.22, 4.47) |  | 0.73 (0.44, 1.1) | 1.73 (0.81, 3.98) |
| Chad | 1.5 (0.94, 2.13) | 5.66 (3.92, 8.48) |  | 2.61 (1.55, 4.05) | 4.67 (2.92, 7.05) |
| Chile | 2741.87 (2461.9, 3030.04) | 3986.4 (3606.11, 4345.86) |  | 5920.89 (4675.7, 7315.73) | 6274.63 (5017.34, 7481.39) |
| China | 221399.68 (172917.1, 276617) | 466625.94 (340087.81, 608407.28) |  | 994494.64 (684555.14, 1394041.7) | 1494584.43 (1025371.69, 1997374.34) |
| Colombia | 1467.44 (1232.87, 1728.41) | 1939.97 (1563.46, 2354.79) |  | 4810.22 (3306.41, 6561.58) | 4863.76 (3084.95, 6905.62) |
| Comoros | 0.79 (0.47, 1.17) | 1.48 (0.84, 2.21) |  | 2.26 (1.25, 3.49) | 3.36 (1.72, 5.2) |
| Congo | 8.83 (5.18, 13.73) | 19.58 (13.96, 26.96) |  | 4.01 (2.12, 6.48) | 7.28 (4.85, 10.74) |
| Costa Rica | 130.97 (114.12, 148.74) | 217.49 (188.2, 251.19) |  | 542.08 (379.39, 757.71) | 630.87 (422.76, 894.5) |
| Croatia | 2697.75 (2243.62, 3228.27) | 6973.73 (5968.25, 8034.36) |  | 7301.69 (5249.84, 9953.99) | 15997.81 (11662.98, 20827.84) |
| Cyprus | 279.48 (211.4, 342.59) | 959.76 (767.24, 1161.73) |  | 604.05 (410.5, 838.33) | 1371.57 (979.16, 1889.98) |
| Czechia | 7810.12 (6745.67, 8902.01) | 15134.74 (13181.22, 17255.16) |  | 17964.22 (13158.29, 23714.43) | 29268.3 (21953.57, 38010.39) |
| Democratic Republic of the Congo | 9.51 (5.71, 14.48) | 25.74 (13.83, 50.69) |  | 103.04 (53.67, 169.37) | 207.59 (92.56, 463.72) |
| Denmark | 10551.45 (9396.72, 11507.47) | 11052.77 (9989.18, 12237.48) |  | 21092.83 (16409.43, 25387.75) | 15836.1 (12614.77, 19309) |
| Djibouti | 2.02 (1.32, 2.9) | 6.99 (4.11, 10.66) |  | 17.25 (11.05, 26.44) | 35.53 (19.61, 55.69) |
| Dominican Republic | 403.16 (279.59, 557.24) | 729.01 (479.57, 1011.1) |  | 2813.99 (1639.58, 4170.32) | 4028.87 (2194.35, 6456.31) |
| Ecuador | 182.69 (144.02, 229.33) | 226.67 (170.98, 295.2) |  | 353.19 (241.44, 501.22) | 321.89 (202.13, 480.84) |
| Egypt | 1051.13 (830.62, 1301.67) | 3092.42 (2406.38, 3936.4) |  | 3233.1 (2136.05, 4624.58) | 7024.6 (4516.25, 10174.77) |
| El Salvador | 72.21 (56.26, 93.02) | 75.01 (58.67, 96.44) |  | 271.15 (176.63, 407.43) | 194.12 (117.85, 297.25) |
| Equatorial Guinea | 11.29 (6.23, 18.69) | 21.94 (14.26, 31.82) |  | 4.47 (2.5, 7.41) | 8.17 (5.08, 12.07) |
| Estonia | 543.03 (457.38, 627.29) | 1798.64 (1522.96, 2055.39) |  | 1484.73 (1034.57, 2074.33) | 3817.93 (2717.61, 5282.06) |
| Ethiopia | 23 (18.02, 29.2) | 91.64 (69.14, 117.63) |  | 509.35 (343.16, 713.51) | 1460.39 (994.91, 2048.09) |
| Fiji | 10.11 (7.44, 13.29) | 17.82 (13.15, 23.61) |  | 24.74 (15.63, 35.84) | 36.67 (24.52, 51.91) |
| Finland | 3742.07 (3306.41, 4131.18) | 6919.44 (6159.53, 7688.41) |  | 5257.88 (4043.22, 6452.24) | 7171.5 (5680.55, 9005.91) |
| France | 48262.66 (42431.72, 53622.94) | 115183.93 (104244.14, 125906.55) |  | 75107.08 (58970.48, 92772.27) | 140551.2 (114244.19, 172425.18) |
| Gabon | 19.9 (13.02, 29.39) | 58.22 (41.01, 80.76) |  | 29.33 (17.42, 44.41) | 52.88 (34.88, 74.8) |
| Gambia | 0.21 (0.12, 0.33) | 0.73 (0.52, 1.01) |  | 0.51 (0.29, 0.82) | 1.04 (0.66, 1.52) |
| Georgia | 153.9 (132.26, 179.19) | 968.85 (834.13, 1115.66) |  | 464.55 (332.58, 649.57) | 3439.25 (2421.62, 4825.68) |
| Germany | 91943.61 (83646.4, 98960.99) | 155860.09 (143207.55, 169215.44) |  | 124420.56 (99171.09, 149848.81) | 164082.2 (135897.4, 195107.16) |
| Ghana | 28.43 (21.16, 37.79) | 96.88 (69.27, 124.95) |  | 90.07 (59.8, 131.88) | 241.97 (163.67, 337.83) |
| Greece | 4657.6 (4206.92, 5049.46) | 15927.06 (14805.52, 17008.33) |  | 5027.32 (3868.27, 6389.7) | 12539.09 (9709.66, 15739.37) |
| Grenada | 2.3 (1.96, 2.65) | 4.91 (4.11, 5.76) |  | 6.23 (4.28, 8.77) | 8.79 (5.45, 13.55) |
| Guatemala | 82.23 (70.01, 96.1) | 83.9 (70.22, 99.97) |  | 242.24 (172.37, 332.02) | 175.8 (110.93, 250.68) |
| Guinea | 3.23 (1.85, 4.68) | 13.6 (9.43, 19.32) |  | 20.24 (11.95, 30.91) | 44.92 (29.42, 67.02) |
| Guinea-Bissau | 0.51 (0.36, 0.71) | 1.35 (0.94, 1.94) |  | 3.8 (2.34, 5.66) | 6.99 (4.43, 11.05) |
| Guyana | 44.39 (32.04, 59.79) | 67.6 (49.1, 88.92) |  | 518.86 (327.71, 756.73) | 532.63 (324.5, 815.35) |
| Haiti | 15.91 (10.34, 23.55) | 31.98 (17.82, 57.28) |  | 27.52 (14.79, 48.32) | 41.63 (19.45, 84.42) |
| Honduras | 78.55 (47.07, 129.65) | 70.79 (53.19, 96.52) |  | 408.61 (210.46, 735.9) | 259.2 (151.21, 424.17) |
| Hungary | 12023.23 (10153.92, 13836.29) | 18217.73 (15694.91, 20898.66) |  | 24913.82 (17665.63, 33311.02) | 30237.49 (21134.27, 40449.91) |
| Iceland | 347.24 (305.95, 387.76) | 241.21 (213.45, 270.12) |  | 832.08 (648.73, 1027.04) | 558.89 (440.33, 685.74) |
| India | 4725.64 (3925.66, 5655.81) | 10626.6 (8458.33, 12718.81) |  | 20162.97 (13137.34, 28962.05) | 29001.62 (18617.75, 41907.62) |
| Indonesia | 77876.19 (44378.69, 111777.52) | 165279.42 (112468.07, 222023.37) |  | 227329.43 (120686.14, 339289.69) | 367600.82 (244095.43, 503872.01) |
| Iran (Islamic Republic of) | 1605.52 (1349.42, 1836.3) | 3501.89 (3152.48, 3900.66) |  | 2327.24 (1513.56, 3369.28) | 3734.61 (2441.76, 5199.19) |
| Iraq | 273.89 (193.65, 373.53) | 738.03 (519.22, 944.35) |  | 150.08 (79.54, 238.27) | 307.74 (165.29, 495.12) |
| Ireland | 5068.55 (4339.95, 5817.2) | 6051.24 (5473.66, 6634.16) |  | 41785.75 (32880.31, 52227.15) | 35442.65 (29324.53, 42564.99) |
| Israel | 1876.18 (1655.48, 2065.65) | 3929.44 (3550.42, 4316.88) |  | 3959.07 (2903.06, 5121.77) | 6143.59 (4715.81, 7923.87) |
| Italy | 42480.76 (37206.31, 46052.37) | 95497.16 (88890.79, 100517.2) |  | 54321.67 (42464.37, 69199.21) | 99715.54 (80861.44, 120707.98) |
| Jamaica | 37.82 (28.6, 48.36) | 107.01 (79.57, 143.17) |  | 73.09 (45.99, 106.27) | 144.31 (88.75, 219.41) |
| Japan | 72688.92 (58614.47, 80601.5) | 189293.64 (174955.99, 197562.09) |  | 66116.08 (47968.33, 85256.79) | 116886.54 (94163.08, 141787.27) |
| Jordan | 46.74 (31.33, 65.01) | 176.22 (132.42, 235.95) |  | 83.6 (49.08, 131.86) | 250.95 (157.91, 371.17) |
| Kazakhstan | 719.99 (593.83, 852.93) | 3075.28 (2608.24, 3592.19) |  | 1512.8 (968.39, 2170.68) | 4338.82 (2770.06, 6327.25) |
| Kenya | 34.17 (22.8, 53.7) | 51.5 (39.4, 65.01) |  | 498.31 (318.33, 780.99) | 493.71 (357.11, 658.32) |
| Kiribati | 0.23 (0.15, 0.33) | 0.86 (0.63, 1.17) |  | 0.86 (0.5, 1.34) | 2.37 (1.55, 3.37) |
| Kuwait | 235.81 (200.11, 280.2) | 888.09 (703.23, 1114.43) |  | 215.77 (145.02, 297.54) | 702.85 (442.99, 1022.22) |
| Lao People's Democratic Republic | 63.76 (41.41, 104.67) | 156.73 (108.69, 217.73) |  | 587.05 (341.28, 1020.95) | 1100.27 (746.44, 1596.4) |
| Latvia | 661.09 (551.63, 768.38) | 2350.11 (1962.49, 2678.52) |  | 1444.33 (1039.53, 1965.61) | 3923.21 (2787.53, 5268.08) |
| Lebanon | 164.58 (111.12, 222.65) | 383.45 (287.13, 493.13) |  | 119.69 (72.06, 186.81) | 227.13 (144.02, 351.76) |
| Lesotho | 3.95 (2.21, 6.21) | 14.37 (9.58, 21.37) |  | 5.11 (2.68, 8.79) | 17.76 (11.64, 25.94) |
| Liberia | 0.53 (0.31, 0.76) | 1.3 (0.77, 2.24) |  | 3.08 (1.75, 4.81) | 5.27 (3.07, 8.54) |
| Libya | 156.98 (99.38, 230.48) | 1109.92 (771.32, 1552.83) |  | 157.1 (91.03, 256.82) | 884.87 (521.09, 1379.97) |
| Lithuania | 1062.45 (894.53, 1252.71) | 4356.58 (3696.71, 5007.21) |  | 3734.66 (2626.08, 5051.24) | 10334.74 (7380.04, 13970.26) |
| Luxembourg | 595.56 (530.03, 665.72) | 1235.31 (1093.36, 1378.89) |  | 985.27 (770.88, 1190.42) | 1630.71 (1298.47, 1970.94) |
| Madagascar | 4.34 (2.91, 6.09) | 8.16 (5.74, 11.08) |  | 9.65 (6.35, 14.15) | 12.3 (8.3, 17.64) |
| Malawi | 0.69 (0.39, 1.04) | 3.15 (2.2, 4.44) |  | 1.59 (0.89, 2.53) | 5.25 (3.4, 7.79) |
| Malaysia | 2065.47 (1721.43, 2475.92) | 5114.72 (4226.14, 5929.58) |  | 6313.82 (4491.26, 8950.2) | 10652.46 (7659.35, 14509.69) |
| Maldives | 4.4 (3.38, 5.51) | 12.39 (9.11, 15.63) |  | 46.17 (31.94, 65.22) | 139.36 (95.48, 190.28) |
| Mali | 4.19 (2.89, 5.86) | 7.5 (5.06, 10.32) |  | 21.76 (14.28, 33.01) | 24.85 (15.27, 37.15) |
| Malta | 156.01 (133.53, 181.59) | 435.91 (389.81, 483.53) |  | 455.57 (336.5, 583.22) | 1015.42 (759.15, 1297.98) |
| Mauritania | 5.64 (3.94, 7.82) | 10.62 (7.1, 15.99) |  | 21.93 (14.99, 31.99) | 26.89 (16.85, 39.63) |
| Mauritius | 74.02 (67.35, 80.75) | 181.19 (164.71, 196.5) |  | 163.35 (122.67, 211.59) | 283.79 (215.49, 362.4) |
| Mexico | 2753.77 (2332.66, 3183.59) | 4185.82 (3511.34, 4951.41) |  | 4335.56 (3151.1, 5731.62) | 4501.88 (3067.92, 6334.55) |
| Micronesia (Federated States of) | 0.59 (0.41, 0.81) | 1.52 (0.99, 2.28) |  | 0.97 (0.59, 1.47) | 1.82 (1.1, 2.86) |
| Mongolia | 46.89 (35.07, 59.9) | 175.89 (135.76, 227.97) |  | 98.8 (62.52, 145.99) | 277.52 (164.38, 416.63) |
| Montenegro | 156.61 (121.67, 194.31) | 454.82 (343.79, 574.59) |  | 504.83 (343.6, 733.69) | 1045.8 (695.03, 1485.21) |
| Morocco | 97.83 (66.84, 136.41) | 1094.27 (792.22, 1389.42) |  | 229.99 (136.23, 366.79) | 1683.24 (1013.5, 2577.4) |
| Mozambique | 3 (1.86, 4.23) | 7.01 (5.29, 9.11) |  | 9.58 (5.87, 14.62) | 14.21 (9.96, 19.61) |
| Myanmar | 309.11 (227.44, 431.15) | 549.27 (384.83, 746.14) |  | 494.25 (314.7, 716.22) | 692.69 (466.36, 1016.32) |
| Namibia | 10 (6.48, 14.32) | 18.12 (13.39, 23.75) |  | 14.78 (9.7, 22.54) | 20.35 (14.6, 27.86) |
| Nepal | 38.42 (25.62, 60.08) | 71.02 (52.9, 96.5) |  | 219.48 (130.46, 389.35) | 281.41 (177.05, 443.3) |
| Netherlands | 25342.49 (22729.07, 27505.33) | 33154.01 (30465.04, 35522.21) |  | 36657.19 (29427.99, 44479.72) | 39419.87 (31489.55, 48378.64) |
| New Zealand | 3381.42 (3051.25, 3708.02) | 3264.37 (2942.17, 3590.33) |  | 8823.31 (6830.55, 10923.05) | 6722.11 (5319.09, 8409.27) |
| Nicaragua | 12.96 (10.25, 16.37) | 16.3 (12.26, 20.99) |  | 53.47 (35.5, 76.61) | 53.79 (33.75, 82.44) |
| Niger | 1.13 (0.67, 2.02) | 3.17 (2.1, 5.25) |  | 3.29 (1.76, 5.71) | 5.93 (3.61, 10.05) |
| Nigeria | 51.55 (33.9, 74.48) | 86.61 (63.33, 118.25) |  | 142.94 (92.39, 209.63) | 156.54 (107.04, 224.19) |
| North Macedonia | 196.81 (150.75, 249.34) | 774.68 (575.72, 973.14) |  | 563.93 (348.49, 847.27) | 1658.73 (1018.21, 2500.3) |
| Norway | 6486.57 (5843.99, 6927.62) | 7125.01 (6616.48, 7547.9) |  | 8357.77 (6942.73, 9826.82) | 7688.35 (6400.1, 8916.02) |
| Oman | 52.92 (34.72, 73.72) | 204.16 (148.49, 294.26) |  | 57.46 (34.38, 87.27) | 222.57 (131.62, 346.75) |
| Pakistan | 379.11 (249.64, 539.72) | 1744.21 (1242, 2368.19) |  | 1525.43 (907.58, 2466) | 4236.17 (2548.93, 6659.7) |
| Panama | 274.45 (211.4, 334.29) | 457.91 (347.67, 564.55) |  | 2023.82 (1355.65, 2808.12) | 2364.91 (1514.83, 3489.27) |
| Papua New Guinea | 19.93 (11.41, 33.09) | 49.61 (31.1, 78.45) |  | 89.16 (43.32, 154.29) | 144.59 (82.86, 239.14) |
| Paraguay | 109.02 (80.36, 149.22) | 334.47 (248.03, 443.95) |  | 373.07 (225.61, 565.01) | 852.48 (477.63, 1291.89) |
| Peru | 632.24 (450.27, 817.54) | 699.87 (496.97, 899.69) |  | 1479.02 (999.09, 2086.45) | 1257.94 (786.59, 1866.34) |
| Philippines | 1161.78 (907.26, 1455.92) | 2538.34 (1944.64, 3205.86) |  | 3248.11 (2363.53, 4392.02) | 4872.65 (3666.82, 6269.76) |
| Poland | 33817.16 (30195.67, 37329.73) | 66670.19 (59777.73, 73526.81) |  | 87445.66 (65877.74, 113574.02) | 167368.24 (128510.92, 213867.24) |
| Portugal | 3300.03 (2887.84, 3660.35) | 10616.6 (9706.15, 11535.38) |  | 6115.29 (4656.54, 7812.44) | 14006.72 (10959.37, 17446.71) |
| Qatar | 174.91 (121.02, 241.55) | 937.54 (653.73, 1359.3) |  | 291.99 (186.94, 452.43) | 1236.21 (765.59, 1904.09) |
| Republic of Korea | 19775.32 (15669.87, 23873.16) | 54813.84 (46502.17, 63847.22) |  | 53479.6 (37609.08, 72722.5) | 95776.04 (73085.86, 124137.11) |
| Republic of Moldova | 148.96 (130.6, 170.89) | 665.87 (577.46, 768.35) |  | 730.57 (469.96, 1055.56) | 2476.13 (1496.19, 3642.29) |
| Romania | 10113.44 (8713.7, 11655.46) | 31824.61 (27730.15, 36297.32) |  | 34365.2 (23752.77, 46476.45) | 73562.13 (51400.14, 101732.01) |
| Russian Federation | 23558.66 (20957.78, 26012.89) | 97459.52 (86156.46, 107739.85) |  | 42880.31 (31603.42, 58360.72) | 115747.22 (84520.98, 155037.82) |
| Rwanda | 5.31 (2.7, 8.1) | 10.73 (5.55, 15.9) |  | 45.88 (22.34, 74.53) | 78.42 (37.62, 124.12) |
| Saint Lucia | 4.47 (3.64, 5.42) | 8.85 (7, 10.85) |  | 5.32 (3.38, 7.71) | 7.22 (4.29, 11.1) |
| Saint Vincent and the Grenadines | 2.85 (2.46, 3.32) | 5.04 (4.4, 5.78) |  | 6.15 (4, 8.66) | 6.93 (4.15, 10.61) |
| Samoa | 0.36 (0.22, 0.52) | 1.39 (1.03, 1.81) |  | 0.54 (0.3, 0.85) | 1.51 (1.02, 2.21) |
| Sao Tome and Principe | 0.3 (0.18, 0.48) | 0.87 (0.65, 1.16) |  | 1.32 (0.72, 2.19) | 3.42 (2.37, 4.85) |
| Saudi Arabia | 1559.9 (1093.33, 2131.59) | 4547.21 (3467.51, 5954.2) |  | 2999.33 (1858.98, 4862.55) | 7602.91 (4660.8, 11433.21) |
| Senegal | 10.39 (7.77, 13.93) | 27.51 (19.76, 37.41) |  | 47.92 (33.75, 67.18) | 81.84 (56.46, 112.83) |
| Serbia | 2213.72 (1729.19, 2780.71) | 5653.46 (4371.67, 7067.04) |  | 5136.03 (3524.04, 7128.98) | 9134.53 (5952.5, 12868.22) |
| Seychelles | 9.8 (8.12, 11.89) | 31.9 (26.14, 38.83) |  | 28.93 (20.76, 39.47) | 63.51 (46.49, 81.33) |
| Sierra Leone | 1.32 (0.85, 1.81) | 3.64 (2.45, 5.16) |  | 7.93 (4.92, 12.16) | 13.24 (8.57, 20.13) |
| Singapore | 3517.33 (3154.1, 3902.99) | 6452.62 (5730.74, 7286.48) |  | 9027.4 (6725.94, 11416.07) | 11707.97 (9106.11, 14511.33) |
| Slovakia | 1654.27 (1272.47, 2138.28) | 5555.66 (4248.32, 6927.52) |  | 2103.77 (1482.18, 2891.02) | 5344.99 (3568.33, 7305.74) |
| Slovenia | 1122.54 (915.71, 1325.12) | 2301.37 (1975.68, 2637.23) |  | 2365.97 (1754.21, 3169.52) | 3854.36 (2852.47, 5170.46) |
| Solomon Islands | 1.04 (0.73, 1.44) | 2.55 (1.86, 3.57) |  | 2.62 (1.68, 3.89) | 4.18 (2.79, 6) |
| Somalia | 1.69 (1.07, 2.8) | 5.86 (3.45, 12.06) |  | 13.99 (6.6, 25.86) | 39.93 (17.31, 90.64) |
| South Africa | 1811.94 (1577.39, 2068.75) | 4093.51 (3584.74, 4666.08) |  | 2372.67 (1848.29, 2972.26) | 3894.66 (3095.5, 4908.35) |
| Spain | 18047.42 (15916.39, 20068.55) | 58956.04 (54418.64, 63304.66) |  | 23810.09 (18628.11, 30008.74) | 58620.44 (47527.06, 72960.09) |
| Sri Lanka | 338.16 (224.45, 464.68) | 827.57 (512.07, 1160.73) |  | 777.08 (493.13, 1127.94) | 1346.19 (839.6, 1941.69) |
| Sudan | 33.3 (20.99, 52.36) | 85.05 (54.11, 131.84) |  | 72.29 (40.02, 130.15) | 106.46 (57.34, 177.5) |
| Suriname | 15.78 (11.9, 20.44) | 31.33 (23.13, 41.66) |  | 14.71 (9.33, 21.71) | 19.68 (11.36, 29.61) |
| Sweden | 8209.57 (6876.53, 9443.9) | 7068 (5971.32, 8243.35) |  | 13017.33 (10040.73, 16355.87) | 8949.21 (7081.28, 11098.95) |
| Switzerland | 8052.62 (6928.52, 8991.73) | 12250.34 (11013.69, 13472.01) |  | 10282.15 (8072.22, 12303.92) | 11841.85 (9662.85, 14194.34) |
| Tajikistan | 19.52 (11.12, 29.56) | 42.63 (27.76, 63.45) |  | 94.43 (53.08, 164.15) | 130.83 (71.09, 228.87) |
| Thailand | 6914.2 (5190.83, 8890.18) | 13291.23 (9978.33, 16928.72) |  | 11649.28 (7984.34, 16280.92) | 14777.5 (10414.01, 20276.89) |
| Timor-Leste | 4.63 (3.44, 6.16) | 10.7 (7.59, 14.74) |  | 2.62 (1.69, 3.85) | 4.74 (3.1, 6.89) |
| Togo | 2.64 (1.51, 3.87) | 7.42 (4.73, 10.63) |  | 11.08 (5.93, 17.2) | 21.6 (13.12, 33.01) |
| Tonga | 0.94 (0.68, 1.29) | 3.1 (2.38, 3.92) |  | 2.51 (1.63, 3.72) | 5.67 (3.92, 7.75) |
| Tunisia | 89.49 (61.7, 128.7) | 793.4 (520.58, 1127.81) |  | 144.06 (85.36, 234.44) | 760.72 (465.39, 1153.71) |
| Uganda | 17.96 (11.41, 25.95) | 19.75 (14.32, 27.2) |  | 62.27 (38.76, 92.76) | 46.06 (30.56, 65.82) |
| Ukraine | 1736.9 (1153.7, 2501.96) | 7881.54 (5116.94, 11355.3) |  | 3020.06 (1717.33, 4595.33) | 9087.82 (5364.25, 14268.43) |
| United Arab Emirates | 728.14 (480.59, 1070.7) | 2598.54 (1864.37, 4321.69) |  | 5074.4 (2899.69, 8196.88) | 9547.15 (6008.53, 15444.02) |
| United Kingdom | 57932.9 (53391.09, 60502.12) | 67794.72 (64346.58, 70092.1) |  | 93831.47 (76964.34, 114269.51) | 84472.19 (69426.97, 102611.99) |
| United Republic of Tanzania | 24.96 (17.19, 34.55) | 50.18 (33.67, 71.54) |  | 116.58 (75.27, 174.03) | 135.34 (81.71, 204.49) |
| United States of America | 406770.98 (370296.07, 428174.48) | 496512.32 (471816.05, 516558.78) |  | 614779.11 (491037.49, 739713.28) | 527855.88 (442149.11, 622705.1) |
| Uruguay | 791.15 (718.89, 867.21) | 2304.03 (2105.66, 2490.52) |  | 1084.35 (831.12, 1365.1) | 2589.67 (2042.9, 3234.88) |
| Uzbekistan | 160.29 (129.5, 194.82) | 382.34 (310.45, 461.04) |  | 509.23 (291.11, 794.32) | 917.07 (511.45, 1484.57) |
| Vanuatu | 0.44 (0.32, 0.63) | 1.59 (0.98, 2.7) |  | 1.67 (1.06, 2.52) | 3.56 (2.02, 6.63) |
| Viet Nam | 2560.92 (1881.23, 3353.96) | 6549.01 (4743.18, 8509.27) |  | 16623.84 (11401.12, 23023.89) | 29654.28 (20593.78, 40296.33) |
| Zambia | 17.5 (9.66, 30.2) | 36.57 (23.11, 75.81) |  | 41.13 (23.2, 67.67) | 59.75 (35.88, 118.48) |
| Zimbabwe | 15.04 (10.72, 21.26) | 20.53 (14.98, 27.68) |  | 23.36 (14.94, 34.76) | 28.43 (19.8, 41.76) |

Note: Data were available for 169 countries covering more than 99% of the world population. All future year estimates were discounted at a 3% annual discount rate.

**Supplementary Table 17** Estimated economic burden of tracheal, bronchus, and lung cancer by country through 2050, millions of 2023 US$. (E=1.5)

| Location | **2021**  **economic burden 95%UI** | **2030**  **economic burden 95%UI** | **2040**  **economic burden 95%UI** | **2050**  **economic burden 95%UI** |
| --- | --- | --- | --- | --- |
| Afghanistan | 24.3 (15.71, 35.15) | 24.47 (13.44, 39.12) | 25.06 (12.89, 40.54) | 25.02 (11.62, 44.27) |
| Albania | 622.82 (439.11, 840.67) | 831.14 (533.66, 1148.94) | 1064.33 (651.35, 1526.4) | 1306.65 (770.94, 1962.38) |
| Algeria | 842.53 (653.15, 1089.95) | 839.44 (590.82, 1125.96) | 784.8 (530.35, 1094.59) | 681.27 (414.77, 1001.42) |
| Angola | 208.42 (150.82, 278.34) | 208.42 (153.75, 276.81) | 206.46 (150.02, 286.04) | 204.02 (141.84, 293.3) |
| Argentina | 12058.65 (10934.1, 13306.81) | 14370.72 (11961.1, 17187.95) | 16716.02 (13481.04, 20451.56) | 18817.49 (14730.47, 23730.89) |
| Armenia | 688.76 (610.27, 779.92) | 935.48 (747.84, 1143.32) | 1221.85 (930.66, 1637.84) | 1545.95 (1038.21, 2275.56) |
| Australia | 32947.4 (29910.45, 35806.94) | 39398.11 (33867.87, 44352.19) | 45295.57 (37815.24, 52437.58) | 51541.81 (41013.11, 62103.07) |
| Austria | 18715.19 (17198.03, 20239.85) | 20443.44 (17748.76, 23017.48) | 20890.6 (17582.55, 24245.7) | 20770.53 (16908.73, 25184.61) |
| Azerbaijan | 1221.7 (823.93, 1660.48) | 1273.94 (861.74, 1821.78) | 1210.05 (775.59, 1846.22) | 1082.67 (608.93, 1739.3) |
| Bahamas | 113.07 (90.4, 143.92) | 128.17 (97.3, 164.03) | 132.04 (96.46, 176.27) | 127.02 (82.07, 179.58) |
| Bahrain | 622.51 (499.02, 794.61) | 885.87 (689.76, 1174.43) | 1173.24 (835.96, 1596.75) | 1412.09 (913.94, 2122.71) |
| Bangladesh | 1322.57 (967.81, 1833.54) | 2444.44 (1702.94, 3561.17) | 4517.84 (2966.12, 6717.81) | 8116.23 (5002.27, 12936.17) |
| Barbados | 26.1 (19.88, 33.08) | 22.31 (16.65, 29.29) | 16.84 (11.91, 22.73) | 12.16 (7.57, 17.54) |
| Belarus | 4606.65 (3559.11, 5666.86) | 4578.58 (3482.03, 5788.67) | 4256.88 (3012.35, 5768.8) | 3810.29 (2436.08, 5592.39) |
| Belgium | 30985.61 (28122.99, 33654) | 35506.75 (30598.84, 40916.67) | 38212.4 (32018.25, 45376.65) | 40068.02 (32445.88, 49898.02) |
| Belize | 9.62 (8.25, 11.14) | 12.42 (10, 15.12) | 14.95 (10.96, 19.73) | 17.25 (11.07, 24.99) |
| Benin | 19.24 (14.1, 25.91) | 32.25 (23.25, 42.92) | 55.2 (39.34, 75.25) | 95.89 (65.58, 135.77) |
| Bhutan | 13.05 (8.71, 18.84) | 23.2 (15.6, 33.19) | 42.15 (26.45, 63.94) | 72.67 (41.5, 118.1) |
| Bolivia (Plurinational State of) | 262.13 (180.49, 364.68) | 422.09 (273.07, 590.53) | 736.84 (472.21, 1089.44) | 1247.14 (751.84, 1938.98) |
| Bosnia and Herzegovina | 1933.37 (1511.27, 2420.1) | 2624.64 (1987.79, 3401.02) | 3482.37 (2522.73, 4669.45) | 4367.15 (2843.13, 6307.45) |
| Botswana | 166.72 (120.62, 217.5) | 240.38 (167.21, 317.35) | 355.77 (249.89, 477.46) | 495.73 (332.36, 669.72) |
| Brazil | 22068.9 (20854.47, 23222.3) | 22645.88 (18552.05, 27011.54) | 21647.34 (16330.03, 27976.07) | 19894.32 (13294.5, 28710.79) |
| Brunei Darussalam | 461.42 (370.16, 556.27) | 456.72 (354.76, 583.7) | 391.16 (293.49, 520.64) | 302.82 (210.99, 420.47) |
| Bulgaria | 8255.28 (6887.54, 9680.81) | 10164.15 (8126.62, 12646.07) | 12369.3 (9658.44, 15981.8) | 13906.3 (10092.64, 18998.23) |
| Burkina Faso | 16.54 (11.9, 24.68) | 26.09 (18.59, 37.32) | 44.42 (31.24, 65.4) | 77.77 (53.25, 117.42) |
| Burundi | 1.93 (1.35, 2.88) | 2.53 (1.68, 3.95) | 3.54 (2.31, 5.53) | 4.84 (3.04, 7.75) |
| Cabo Verde | 12.07 (8.96, 15.05) | 16.04 (11.64, 20.89) | 21.1 (15.18, 27.79) | 26.66 (18.54, 36.44) |
| Cambodia | 305.62 (217.63, 399.82) | 513.65 (353.13, 691.9) | 866.24 (589.72, 1195.78) | 1456.67 (936.71, 2112.21) |
| Cameroon | 77.27 (52.33, 107.13) | 111.67 (74.81, 156.38) | 168.21 (117.26, 235.76) | 253.02 (171, 364.09) |
| Canada | 70755.67 (65237.39, 76173) | 82650.91 (72239.11, 93299.06) | 87737.41 (74652.81, 103202.38) | 89434.21 (72333.4, 108677.12) |
| Central African Republic | 2.67 (1.6, 5.02) | 2.62 (1.47, 5.07) | 2.57 (1.44, 4.92) | 2.46 (1.3, 4.8) |
| Chad | 7.16 (5.07, 10.34) | 6.87 (4.71, 9.56) | 6.83 (4.71, 9.75) | 7.27 (4.73, 10.57) |
| Chile | 4120.69 (3747.95, 4468.54) | 5259.65 (4496, 6006.67) | 6404.08 (5323.5, 7494.69) | 7469.08 (5981.09, 8966.56) |
| China | 688025.62 (549130.25, 840441.18) | 1089356.66 (877828.18, 1317379.91) | 1699703.99 (1306521.75, 2099423.31) | 2489079.07 (1756365.75, 3322691.21) |
| Colombia | 3407.42 (2835.41, 4140.57) | 4971.06 (3964.11, 6132.82) | 7073.47 (5265.38, 9149.35) | 9673.99 (6424.98, 13279.47) |
| Comoros | 2.28 (1.5, 3.19) | 3.23 (2.07, 4.63) | 4.38 (2.69, 6.43) | 5.62 (3.31, 8.42) |
| Congo | 28.41 (20.65, 38.43) | 22.5 (15.71, 31.09) | 16.48 (11.31, 23.34) | 11.28 (7.47, 16.61) |
| Costa Rica | 348.46 (303.25, 394.9) | 554.32 (451.44, 669.88) | 824.24 (624.19, 1075.71) | 1172.94 (814.49, 1638.59) |
| Croatia | 6828.67 (5935.98, 7713.88) | 9607.82 (7832.5, 11433.09) | 12865.87 (10245.37, 15682.51) | 16450.9 (12252.02, 21176.04) |
| Cyprus | 952.94 (774.91, 1157.38) | 1143.25 (901.73, 1458.95) | 1327.36 (999.11, 1756.06) | 1519.19 (1093.42, 2086.55) |
| Czechia | 17998.96 (15693.19, 20214.77) | 23071.7 (19236.47, 27440.58) | 30038.15 (24087.09, 36865.28) | 37051.26 (28506.48, 47426.14) |
| Democratic Republic of the Congo | 35.25 (21.48, 61.84) | 72.98 (37.7, 141.29) | 152.13 (78.56, 289.96) | 310.63 (158.47, 612.01) |
| Denmark | 21231.98 (19341.88, 23129.79) | 26635.87 (22759.83, 30646.4) | 31114.6 (25977.89, 36181.75) | 36292.65 (29248.42, 43103.69) |
| Djibouti | 9.01 (5.83, 13.25) | 16.93 (10.62, 25.8) | 31 (19.32, 46.53) | 52.78 (32.23, 79.01) |
| Dominican Republic | 1132.17 (765.77, 1539.5) | 2079.74 (1323.9, 2879.89) | 3837.97 (2319.44, 5479.36) | 6842.85 (3812.63, 10378.34) |
| Ecuador | 409.36 (318.89, 520.49) | 501.48 (368.93, 656.29) | 591.7 (419.53, 807.44) | 675.08 (450.19, 971.45) |
| Egypt | 4143.55 (3219.66, 5186.69) | 5866.4 (4517.67, 7385.71) | 8029.29 (5815.98, 10784.22) | 10257.7 (6749.71, 15039.17) |
| El Salvador | 147.22 (117.23, 185.51) | 221.92 (162.12, 303.62) | 327.66 (229.71, 466.63) | 465.27 (299.53, 700.03) |
| Equatorial Guinea | 33.23 (21.17, 48.37) | 23.97 (15.74, 35.05) | 17.7 (11.38, 25.8) | 12.64 (7.74, 18.89) |
| Estonia | 1808.15 (1527.65, 2050.37) | 2375.41 (1929.34, 2871.68) | 3120.17 (2393.15, 3975.59) | 4094.5 (2960.17, 5558.67) |
| Ethiopia | 114.64 (91.29, 143.13) | 282.15 (212.06, 369.61) | 749.81 (546.75, 991.96) | 1969.74 (1365.69, 2665.9) |
| Fiji | 27.93 (21.1, 36.42) | 38.35 (28.07, 49.4) | 49.56 (34.79, 64.71) | 61.41 (40.43, 86.52) |
| Finland | 9428.59 (8516.45, 10305.95) | 10364.45 (8860.3, 11871.03) | 10683.91 (8945.98, 12695.9) | 10992.02 (8776.05, 13390.93) |
| France | 140433.42 (128082.36, 152652.72) | 163125.93 (140123.74, 188657.19) | 176938.8 (147476.61, 206824.46) | 185293.74 (151309.93, 221698.58) |
| Gabon | 78.13 (54.83, 104.31) | 81.71 (55.31, 111.01) | 81.92 (55.1, 112.04) | 82.21 (54.03, 116.49) |
| Gambia | 0.94 (0.66, 1.28) | 1.09 (0.74, 1.5) | 1.29 (0.86, 1.8) | 1.56 (0.99, 2.24) |
| Georgia | 1122.74 (963.64, 1295.71) | 1929.92 (1557.25, 2377.45) | 2874.89 (2208.23, 3744.69) | 3903.8 (2788.4, 5455.04) |
| Germany | 230100.66 (211394.21, 248574.59) | 260403.39 (224660.81, 295158.45) | 266514.69 (223994.34, 306528.6) | 267892.19 (221835.15, 313978.82) |
| Ghana | 125.31 (91.41, 159.03) | 171.62 (127.76, 226.15) | 239.75 (174.43, 319.32) | 332.04 (231.08, 454.28) |
| Greece | 13641.83 (12671.29, 14581.79) | 13893.14 (11653.4, 16166.58) | 13254.87 (10939.43, 15938.97) | 11641.59 (9128.87, 14356.48) |
| Grenada | 7.21 (6.07, 8.37) | 9.84 (7.74, 12.2) | 12.39 (9.12, 16.51) | 15.02 (9.8, 21.97) |
| Guatemala | 166.13 (139.83, 194) | 224.98 (182.57, 274.08) | 308.53 (232.69, 394.33) | 418.04 (284.79, 575.3) |
| Guinea | 16.82 (11.77, 23.82) | 25.06 (18.01, 34.92) | 40.12 (28.24, 56.66) | 65.16 (43.18, 94.23) |
| Guinea-Bissau | 1.86 (1.36, 2.48) | 3.12 (1.8, 4.31) | 5.84 (3.86, 8.36) | 10.79 (7.11, 15.98) |
| Guyana | 63.48 (46.98, 82.38) | 143.89 (105.88, 191.5) | 298.58 (207.44, 411.68) | 596.1 (377.14, 889.88) |
| Haiti | 47.9 (29.16, 75.61) | 54.94 (35.04, 84.86) | 62.67 (37.71, 101.36) | 69.15 (37.06, 119.34) |
| Honduras | 149.34 (103.52, 213.02) | 240.22 (155.14, 367.82) | 401.48 (243.17, 639.41) | 667.82 (378.67, 1141.25) |
| Hungary | 21385.09 (18290.57, 24544.76) | 27174.12 (21526.49, 32882.9) | 33652.84 (25948.72, 43289.32) | 39000.61 (28349.9, 51346.29) |
| Iceland | 519.88 (463.77, 576.63) | 704.97 (597.26, 817.6) | 938.37 (767.24, 1109.79) | 1228.89 (941.94, 1502.5) |
| India | 15352.24 (12630.44, 17777.42) | 23604.12 (18587, 29084.03) | 34959.23 (25321.01, 45282.51) | 49164.59 (32081.59, 69734.98) |
| Indonesia | 243155.61 (164994.52, 318581) | 352118.47 (242067.41, 456381.29) | 472545.4 (310414.63, 625787.41) | 594930.25 (376232.5, 821989.82) |
| Iran (Islamic Republic of) | 5107.41 (4644.57, 5614.32) | 5808.47 (4816.5, 6906.35) | 6191.55 (4630.86, 7863.72) | 6061.85 (4039.67, 8393.79) |
| Iraq | 1011.93 (735.48, 1300.47) | 830.71 (496.74, 1182.74) | 641.6 (367.25, 975.62) | 457.81 (247.38, 753.91) |
| Ireland | 11856.7 (10623.31, 13104.23) | 22659.43 (18978.29, 26004.47) | 43922.33 (36583.69, 51313.09) | 82346.35 (67201.2, 99285.66) |
| Israel | 4538.03 (4102.45, 4956.66) | 5614.43 (4520.34, 6670.39) | 6768.77 (5382.87, 8348.35) | 7896.85 (6026.25, 10127.35) |
| Italy | 113581.26 (104806.21, 119946.95) | 125832.02 (109358.59, 141712.63) | 130905.52 (110049.5, 151901.43) | 126801.02 (102930.71, 152888.42) |
| Jamaica | 144.83 (110.18, 189.72) | 175.29 (127.81, 233.17) | 199.05 (136.55, 276.17) | 217.4 (136.15, 322.02) |
| Japan | 208169.69 (185707.37, 220310.51) | 197973.47 (165881.96, 223205.01) | 171922.43 (140202.82, 199107.05) | 145412.73 (114701.02, 176736.66) |
| Jordan | 222.96 (169.18, 296.84) | 272.45 (201.52, 373.19) | 312.29 (217.82, 447.59) | 334.55 (215.71, 498.35) |
| Kazakhstan | 3795.27 (3260.14, 4406.35) | 4630.48 (3713.32, 5661.18) | 5349.65 (3947.14, 7039.08) | 5851.62 (3870.58, 8420.36) |
| Kenya | 85.68 (66.91, 109.28) | 187.38 (147.09, 246.63) | 435.98 (325.99, 585.94) | 992.02 (696.41, 1395.09) |
| Kiribati | 1.09 (0.79, 1.48) | 1.61 (1.19, 2.18) | 2.29 (1.65, 3.09) | 3.23 (2.17, 4.63) |
| Kuwait | 1024.72 (833.23, 1259.48) | 1071.28 (812.12, 1375.02) | 1021.15 (730.08, 1367.21) | 837.56 (537.07, 1198.1) |
| Lao People's Democratic Republic | 220.49 (159.6, 307.2) | 435.17 (316.03, 604.92) | 866.26 (620.3, 1224.55) | 1687.32 (1147.02, 2516.69) |
| Latvia | 2085.53 (1762.99, 2382.75) | 2617.29 (2159, 3129.28) | 3173.16 (2486.91, 3967.58) | 3717.52 (2710.69, 4873.76) |
| Lebanon | 548.03 (431.1, 694.7) | 460.21 (345.62, 614.98) | 409.23 (296.8, 576.49) | 346.83 (230.96, 510.23) |
| Lesotho | 18.33 (12.8, 25.86) | 17.94 (12.26, 24.84) | 19.91 (13.32, 28.09) | 22.87 (14.91, 33.45) |
| Liberia | 1.83 (1.15, 2.9) | 3.11 (1.9, 4.86) | 5.3 (3.22, 8.38) | 8.35 (4.92, 13.52) |
| Libya | 1266.9 (900.13, 1758.35) | 1355.97 (930.27, 2025.48) | 1303.24 (862.92, 1971.58) | 1041.97 (630.44, 1610.4) |
| Lithuania | 4182.39 (3544.38, 4819.29) | 6303.69 (5263.43, 7408.49) | 8467.44 (6702.22, 10546.22) | 10858.71 (7790.81, 14469.98) |
| Luxembourg | 2061.52 (1852.39, 2283.17) | 2512.18 (2153.39, 2886.26) | 2793.77 (2331.99, 3272.86) | 2945.53 (2379.95, 3504.99) |
| Madagascar | 12.5 (9.04, 16.73) | 15.36 (11.03, 20.48) | 18.68 (13.13, 25.26) | 21.94 (15.11, 31.1) |
| Malawi | 3.85 (2.86, 5.16) | 4.5 (3.27, 6.13) | 5.57 (3.87, 7.82) | 6.83 (4.46, 9.75) |
| Malaysia | 7180.19 (6046.95, 8330.64) | 9941.08 (8038.53, 12334.33) | 13230.34 (10262.56, 17037.63) | 16966.28 (12204.66, 23195.92) |
| Maldives | 16.79 (12.97, 20.8) | 36.11 (26.52, 46.16) | 84.51 (62.11, 110.31) | 185.52 (131.79, 252.21) |
| Mali | 11.69 (8.68, 15.63) | 17.86 (12.9, 24.36) | 28.36 (19.51, 39.5) | 46.61 (31.26, 66.96) |
| Malta | 474.09 (420.62, 533.71) | 659.33 (536.73, 790.87) | 891.67 (704.97, 1094.75) | 1178.17 (884.92, 1487.72) |
| Mauritania | 16.26 (11.73, 22.97) | 22.72 (16.28, 31.18) | 33.06 (22.96, 45.36) | 48.81 (32.49, 68.09) |
| Mauritius | 255.21 (233.59, 275.59) | 337.03 (285.17, 395.23) | 398.19 (322.64, 484.44) | 447.15 (343.03, 566.89) |
| Mexico | 6939.6 (6128.43, 7778.02) | 8088.58 (6892.64, 9441.72) | 8728.87 (6886.46, 10765.49) | 8837.44 (6365.9, 11829.66) |
| Micronesia (Federated States of) | 2.11 (1.45, 2.9) | 2.51 (1.74, 3.52) | 2.68 (1.82, 3.79) | 2.79 (1.73, 4.13) |
| Mongolia | 222.78 (173.18, 285.1) | 288.94 (217.4, 368.93) | 343.54 (239.87, 458.02) | 376.32 (229.63, 559.32) |
| Montenegro | 611.43 (481.85, 756.1) | 871.86 (645.65, 1120.57) | 1186.56 (870, 1576.04) | 1550.63 (1053.76, 2150.99) |
| Morocco | 1192.11 (867.33, 1515.16) | 1494.55 (1086.42, 1974.44) | 1750.36 (1183.21, 2411.05) | 1913.22 (1162.89, 2944.66) |
| Mozambique | 10.01 (7.31, 12.92) | 12.89 (9.61, 17.04) | 17.55 (12.42, 23.8) | 23.79 (16.1, 33.41) |
| Myanmar | 858.38 (628.32, 1131.85) | 1011.02 (737.77, 1332) | 1112.09 (799.95, 1495.23) | 1186.95 (814.84, 1695.05) |
| Namibia | 28.12 (20.28, 37.55) | 30.01 (21.88, 40.18) | 32.83 (23.94, 44.89) | 35.12 (25.07, 49.38) |
| Nepal | 109.44 (80.77, 152.52) | 182.54 (127.6, 257.45) | 304.52 (208.51, 451) | 500.88 (320.88, 800.86) |
| Netherlands | 56142.33 (51242, 60159.61) | 66632.76 (57148.62, 76367.26) | 71079.6 (59534.34, 83054.21) | 73015.36 (59570.06, 87419.92) |
| New Zealand | 5410.22 (4966.36, 5889.57) | 7464.99 (6409.52, 8634.69) | 9801.53 (8183.33, 11605.03) | 12655.26 (10103.95, 15620.34) |
| Nicaragua | 29.26 (23.4, 36.37) | 47.84 (35.71, 63.66) | 74 (52.73, 102.23) | 107.26 (71.85, 156.2) |
| Niger | 4.29 (2.9, 7.34) | 5.36 (3.6, 9.05) | 6.7 (4.32, 11.13) | 9.21 (5.65, 15.46) |
| Nigeria | 138.16 (106.74, 178.33) | 180.1 (133.9, 234.97) | 233.67 (172.56, 309.09) | 299.49 (210.36, 412.1) |
| North Macedonia | 971.49 (736.01, 1219.42) | 1315.9 (908.45, 1794.64) | 1776.08 (1177.55, 2482.7) | 2222.66 (1353.61, 3295.44) |
| Norway | 14769.79 (13549.33, 15589.54) | 16503.33 (14537.3, 18493.22) | 17144.12 (14735.78, 19378.09) | 17411.5 (14708.28, 19894.12) |
| Oman | 181.05 (133.35, 249.41) | 207.04 (148.14, 287.96) | 216.62 (145.76, 310.63) | 197.21 (117.4, 300.23) |
| Pakistan | 2123.32 (1590.72, 2790.2) | 3032.41 (2212.07, 4089.24) | 4272.77 (2913.08, 6029.18) | 5761.61 (3666, 8853.69) |
| Panama | 483.48 (371.81, 588.51) | 897.57 (678.02, 1141.86) | 1649.15 (1181.66, 2176.83) | 2897.3 (1949.89, 4173.73) |
| Papua New Guinea | 69.54 (45.29, 109.25) | 107.57 (67.76, 164.27) | 162.01 (99.05, 254.55) | 233.75 (133.09, 392.39) |
| Paraguay | 443.49 (334.68, 587.14) | 657.85 (464.49, 921.72) | 922.01 (593.93, 1298.33) | 1225.54 (706.55, 1849.92) |
| Peru | 1332.11 (962.3, 1704.01) | 1708.53 (1239.15, 2278.36) | 2205.26 (1544.19, 2988.41) | 2736.97 (1838.28, 3942.66) |
| Philippines | 3700.12 (3029.69, 4431.68) | 4997.93 (3967.32, 6078.61) | 6495.69 (5117.98, 8041.15) | 8120.76 (6105.17, 10360.93) |
| Poland | 71993.1 (65455.5, 78269.21) | 99129.34 (84249.68, 114715.41) | 137019.19 (113277.75, 163021.65) | 182558.74 (141870.51, 228182.77) |
| Portugal | 9949.92 (9097.89, 10792.29) | 11745.63 (9783.33, 13749.57) | 13169.37 (10693.53, 15850.32) | 14386.56 (11367.75, 17878.85) |
| Qatar | 1325.15 (967.16, 1886.1) | 1826.46 (1295.45, 2545.94) | 1979.87 (1356.56, 2836.03) | 1820.38 (1175.76, 2725.81) |
| Republic of Korea | 61017.27 (51383.11, 71472.02) | 81341.15 (68077.28, 99180.89) | 105291.43 (84776.09, 131451.54) | 122097.79 (92226.35, 157956.18) |
| Republic of Moldova | 814.84 (714.92, 926.47) | 1341.91 (1022.9, 1680.36) | 2174.51 (1515.46, 2921.76) | 3206.7 (1984.17, 4628.93) |
| Romania | 29585.81 (25916.83, 33532.74) | 42963.88 (34544.88, 52941.55) | 59700.57 (44986.99, 77603.15) | 76138.92 (53826.03, 104672.99) |
| Russian Federation | 121018.18 (109130.19, 132014.7) | 139290.48 (117211.48, 164580.09) | 149812.38 (120175.22, 185959.69) | 158627.53 (116455.15, 208084.19) |
| Rwanda | 16.04 (10.7, 22.5) | 31.33 (20.56, 45.95) | 62.51 (40.33, 90.25) | 124.3 (77.32, 183.37) |
| Saint Lucia | 13.32 (10.71, 16.28) | 13.99 (10.46, 18.32) | 13.61 (9.39, 18.46) | 12.54 (7.65, 18.76) |
| Saint Vincent and the Grenadines | 7.89 (6.91, 9.05) | 10.05 (8.12, 12.51) | 11.76 (8.55, 15.55) | 13.08 (8.32, 19.04) |
| Samoa | 1.75 (1.27, 2.3) | 1.95 (1.41, 2.66) | 2.01 (1.43, 2.82) | 2.04 (1.38, 2.99) |
| Sao Tome and Principe | 1.17 (0.87, 1.6) | 1.91 (1.37, 2.62) | 3.1 (2.23, 4.34) | 4.75 (3.24, 6.88) |
| Saudi Arabia | 5246.74 (4016.84, 6631.36) | 7653.26 (5544.1, 10458.89) | 9116.44 (6318.5, 12722.67) | 9108.58 (5817.43, 13829.51) |
| Senegal | 37.9 (28.08, 51.1) | 55.72 (41.5, 74.25) | 84.6 (62.5, 112.7) | 129.76 (92.56, 175.3) |
| Serbia | 7867.18 (6195.41, 9723.18) | 9692.24 (7225.67, 12285.11) | 11913.21 (8608.86, 16080.19) | 14270.56 (9796.71, 19767.87) |
| Seychelles | 26.55 (22.22, 31.42) | 37.81 (30.55, 46.63) | 49.38 (38.89, 62.13) | 58.86 (43.73, 76.72) |
| Sierra Leone | 4.96 (3.44, 6.94) | 7.86 (5.35, 10.99) | 12.96 (8.81, 18.53) | 21.16 (13.71, 32.15) |
| Singapore | 11787 (10588.72, 13211.07) | 16161.88 (13816.9, 18840.56) | 20760.27 (16860.86, 24792.03) | 24514.46 (18882.07, 30441.96) |
| Slovakia | 4950.19 (3857.28, 6034.07) | 5307.35 (4074.32, 6742.15) | 5386 (3979.2, 7038.78) | 5114.16 (3625.89, 6796.26) |
| Slovenia | 3423.91 (2897.38, 3941.8) | 4378.48 (3572.05, 5212.77) | 5345.22 (4203.06, 6728.81) | 6220.34 (4680.76, 8150.76) |
| Solomon Islands | 3.59 (2.64, 4.93) | 4.63 (3.39, 6.16) | 5.72 (4.08, 7.81) | 6.8 (4.56, 9.85) |
| Somalia | 7.55 (4.57, 14.51) | 14.14 (7.56, 29.32) | 28.34 (14.48, 59.07) | 53.92 (24.24, 119.83) |
| South Africa | 5905.45 (5305.14, 6597.48) | 6264.33 (5310.87, 7340.22) | 6469.05 (5441.96, 7735.64) | 6267.33 (5006.37, 7688.53) |
| Spain | 58817.68 (54071.18, 63314.11) | 64828.34 (56140.28, 73843) | 65832.01 (55252.2, 77405.25) | 62963.04 (51634.75, 77129.67) |
| Sri Lanka | 1165.73 (745.96, 1618.42) | 1530.05 (1019.02, 2108.58) | 1850 (1229.9, 2553.46) | 2123.27 (1342.12, 3012.54) |
| Sudan | 118.35 (78.22, 175.81) | 136.31 (89.21, 198.74) | 158.58 (103.11, 238.98) | 178.75 (103.71, 287.12) |
| Suriname | 47.11 (35.42, 61.66) | 45.92 (33.18, 61.24) | 40.68 (27.32, 56.76) | 34.39 (20.8, 51.74) |
| Sweden | 14352.86 (12454.52, 16225.38) | 16763.22 (13882.75, 19659.02) | 18541.7 (15244.76, 22180) | 20636.97 (16338.98, 25360.44) |
| Switzerland | 20980.97 (18859.7, 22507.19) | 23033.35 (19647.16, 26401.57) | 23456.71 (19599.09, 27087.98) | 22862.81 (18726.56, 26684.6) |
| Tajikistan | 62.14 (41.9, 90.19) | 100.16 (64.43, 148.3) | 152.61 (92.47, 243.66) | 225.25 (128.3, 388.3) |
| Thailand | 20205.43 (15365.62, 25320.5) | 24240.53 (18126.92, 32019.34) | 26355.2 (19181.45, 34641.92) | 26426.77 (18546.03, 35925.85) |
| Timor-Leste | 15.33 (11.32, 20.12) | 12.44 (9.26, 16.51) | 9.33 (6.75, 12.63) | 7.36 (4.9, 10.27) |
| Togo | 10.06 (6.34, 14) | 15.31 (9.86, 21.53) | 22.79 (14.63, 33.05) | 32.68 (20.33, 48.96) |
| Tonga | 4.05 (3.09, 5.18) | 5.23 (3.94, 6.74) | 6.63 (4.84, 8.79) | 8.18 (5.5, 11.35) |
| Tunisia | 882.89 (587.27, 1251.24) | 965.58 (657.96, 1376.27) | 978.44 (654.62, 1414.29) | 904.78 (557.45, 1377.46) |
| Uganda | 37.7 (27.18, 50.64) | 52.74 (37.11, 71) | 75.54 (51.48, 103.21) | 108.33 (71.2, 152.6) |
| Ukraine | 9618.44 (6682.56, 13106.09) | 10988.12 (7598.39, 15085.92) | 11957.98 (7758.47, 17413.09) | 12107.89 (7292.36, 18800.95) |
| United Arab Emirates | 3464.37 (2560.84, 5436.47) | 7521.95 (5335.66, 11396.71) | 11936.02 (8096.17, 17818.03) | 15226.73 (9756.6, 23512.48) |
| United Kingdom | 106084.31 (99493.79, 109993.29) | 124732.72 (111224.33, 136526.85) | 138764.94 (120633.3, 157627.95) | 150446.02 (124871.75, 180211.53) |
| United Republic of Tanzania | 75.14 (55.37, 102.31) | 114.45 (80.2, 155.78) | 170.59 (118.13, 236.61) | 251.92 (169.74, 359.93) |
| United States of America | 903283.29 (844856.87, 942827.12) | 1068186.66 (945656.13, 1169220.02) | 1127918.43 (969453.17, 1284772.82) | 1142634.98 (947631.96, 1352634.92) |
| Uruguay | 1765.39 (1629.9, 1893.22) | 1904.94 (1574.41, 2220.5) | 2003.68 (1629.31, 2385.01) | 2095.54 (1666.43, 2574.13) |
| Uzbekistan | 542.63 (441.74, 654.22) | 796.33 (547.44, 1078.73) | 1089.22 (692.03, 1597.16) | 1426.3 (810.88, 2274.31) |
| Vanuatu | 2.03 (1.33, 3.2) | 2.8 (1.89, 4.44) | 3.85 (2.56, 6.16) | 5.23 (3.19, 8.86) |
| Viet Nam | 9109.93 (6712.53, 11449.32) | 16969.26 (12640.82, 21367.2) | 29145.37 (21425.89, 37627.93) | 46278.11 (32849.28, 62532.47) |
| Zambia | 54.07 (36.76, 96.04) | 65.68 (45.53, 119.84) | 82.23 (57.57, 139.41) | 100.89 (68.92, 163.9) |
| Zimbabwe | 35.57 (26.98, 47.28) | 39.13 (28.73, 51.84) | 45.33 (33.66, 61.58) | 51.79 (36.75, 72.92) |

Note: Data were available for 169 countries covering more than 99% of the world population. All future year estimates were discounted at a 3% annual discount rate.

**Supplementary Table 18** Estimated economic burden of tracheal, bronchus, and lung cancer by country through 2050, millions of 2023 US$. (E=1.0)

| Location | **2021**  **economic burden 95%UI** | **2030**  **economic burden 95%UI** | **2040**  **economic burden 95%UI** | **2050**  **economic burden 95%UI** |
| --- | --- | --- | --- | --- |
| Afghanistan | 158.09 (102.25, 228.71) | 159.22 (87.46, 254.53) | 163.06 (83.86, 263.82) | 162.78 (75.6, 288.06) |
| Albania | 1344.67 (948.03, 1815) | 1794.42 (1152.17, 2480.55) | 2297.87 (1406.25, 3295.47) | 2821.03 (1664.45, 4236.75) |
| Algeria | 2055.56 (1593.5, 2659.18) | 2048 (1441.44, 2747.05) | 1914.7 (1293.91, 2670.5) | 1662.13 (1011.93, 2443.2) |
| Angola | 718.2 (519.73, 959.17) | 718.21 (529.82, 953.87) | 711.47 (516.97, 985.69) | 703.06 (488.78, 1010.72) |
| Argentina | 21046.59 (19083.85, 23225.07) | 25081.96 (20876.32, 29999.02) | 29175.34 (23529.15, 35695.16) | 32843.13 (25709.84, 41418.75) |
| Armenia | 1488.98 (1319.3, 1686.04) | 2022.33 (1616.69, 2471.64) | 2641.41 (2011.92, 3540.72) | 3342.06 (2244.41, 4919.35) |
| Australia | 37275.83 (33839.9, 40511.04) | 44574 (38317.23, 50178.92) | 51246.24 (42783.18, 59326.52) | 58313.06 (46401.17, 70261.8) |
| Austria | 20249.38 (18607.85, 21899.03) | 22119.31 (19203.73, 24904.35) | 22603.13 (19023.89, 26233.26) | 22473.21 (18294.83, 27249.13) |
| Azerbaijan | 2608.72 (1759.35, 3545.67) | 2720.27 (1840.1, 3890.1) | 2583.85 (1656.13, 3942.27) | 2311.86 (1300.26, 3713.97) |
| Bahamas | 168.21 (134.48, 214.1) | 190.67 (144.76, 244.02) | 196.43 (143.5, 262.23) | 188.96 (122.09, 267.16) |
| Bahrain | 732.51 (587.2, 935.03) | 1042.42 (811.65, 1381.97) | 1380.57 (983.69, 1878.92) | 1661.63 (1075.45, 2497.83) |
| Bangladesh | 4255.66 (3114.14, 5899.82) | 7865.52 (5479.59, 11458.86) | 14537.14 (9544.15, 21616.02) | 26115.73 (16095.91, 41624.95) |
| Barbados | 57.44 (43.75, 72.79) | 49.1 (36.64, 64.46) | 37.06 (26.2, 50.02) | 26.77 (16.67, 38.6) |
| Belarus | 8454.04 (6531.62, 10399.73) | 8402.53 (6390.16, 10623.27) | 7812.16 (5528.21, 10586.8) | 6992.58 (4470.66, 10263.07) |
| Belgium | 33698.04 (30584.84, 36600.03) | 38614.97 (33277.42, 44498.46) | 41557.47 (34821.08, 49348.86) | 43575.52 (35286.15, 54266.03) |
| Belize | 26.41 (22.67, 30.59) | 34.1 (27.47, 41.51) | 41.05 (30.11, 54.2) | 47.38 (30.4, 68.63) |
| Benin | 84.95 (62.26, 114.39) | 142.36 (102.63, 189.49) | 243.66 (173.66, 332.19) | 423.29 (289.51, 599.36) |
| Bhutan | 31.67 (21.15, 45.73) | 56.33 (37.87, 80.57) | 102.31 (64.21, 155.22) | 176.41 (100.74, 286.69) |
| Bolivia (Plurinational State of) | 751.25 (517.27, 1045.16) | 1209.68 (782.61, 1692.42) | 2111.75 (1353.32, 3122.27) | 3574.23 (2154.74, 5557.01) |
| Bosnia and Herzegovina | 3928.25 (3070.62, 4917.19) | 5332.78 (4038.83, 6910.24) | 7075.52 (5125.72, 9487.45) | 8873.24 (5776.71, 12815.55) |
| Botswana | 348.38 (252.04, 454.5) | 502.31 (349.39, 663.14) | 743.42 (522.18, 997.7) | 1035.88 (694.5, 1399.47) |
| Brazil | 46899.36 (44318.53, 49350.48) | 48125.5 (39425.57, 57403.12) | 46003.48 (34703.49, 59452.88) | 42278.08 (28252.57, 61014.24) |
| Brunei Darussalam | 476.24 (382.05, 574.14) | 471.39 (366.16, 602.45) | 403.73 (302.92, 537.36) | 312.55 (217.77, 433.98) |
| Bulgaria | 13434 (11208.24, 15753.8) | 16540.34 (13224.63, 20579.22) | 20128.82 (15717.38, 26007.53) | 22630.03 (16423.97, 30916.24) |
| Burkina Faso | 92.19 (66.33, 137.56) | 145.4 (103.62, 208.02) | 247.55 (174.08, 364.51) | 433.44 (296.76, 654.42) |
| Burundi | 18.33 (12.78, 27.31) | 24.03 (15.92, 37.49) | 33.58 (21.89, 52.5) | 45.96 (28.8, 73.52) |
| Cabo Verde | 38.71 (28.75, 48.29) | 51.46 (37.35, 67.01) | 67.7 (48.7, 89.16) | 85.55 (59.49, 116.93) |
| Cambodia | 1207.04 (859.54, 1579.09) | 2028.68 (1394.68, 2732.67) | 3421.23 (2329.1, 4722.74) | 5753.15 (3699.56, 8342.2) |
| Cameroon | 325.82 (220.66, 451.77) | 470.92 (315.46, 659.44) | 709.31 (494.49, 994.18) | 1066.97 (721.1, 1535.36) |
| Canada | 82186.39 (75776.63, 88478.91) | 96003.34 (83909.49, 108371.73) | 101911.58 (86713.13, 119874.94) | 103882.5 (84019.02, 126234.14) |
| Central African Republic | 22.67 (13.54, 42.59) | 22.21 (12.5, 42.98) | 21.82 (12.23, 41.7) | 20.86 (11.02, 40.7) |
| Chad | 48.48 (34.35, 70.06) | 46.52 (31.94, 64.78) | 46.28 (31.89, 66.05) | 49.27 (32.01, 71.62) |
| Chile | 6728.26 (6119.66, 7296.25) | 8587.97 (7341.07, 9807.7) | 10456.59 (8692.22, 12237.35) | 12195.52 (9765.93, 14640.62) |
| China | 1319138.91 (1052837.36, 1611362.46) | 2088603.55 (1683043.87, 2525788.35) | 3258811.29 (2504970.2, 4025185.82) | 4772265.67 (3367447.86, 6370534.94) |
| Colombia | 7009.19 (5832.55, 8517.31) | 10225.67 (8154.33, 12615.45) | 14550.41 (10831.1, 18820.58) | 19899.77 (13216.43, 27316.38) |
| Comoros | 10.16 (6.7, 14.24) | 14.39 (9.22, 20.65) | 19.52 (11.98, 28.65) | 25.07 (14.74, 37.52) |
| Congo | 121.02 (87.95, 163.72) | 95.84 (66.9, 132.44) | 70.21 (48.17, 99.42) | 48.07 (31.81, 70.73) |
| Costa Rica | 637.23 (554.55, 722.16) | 1013.68 (825.54, 1225) | 1507.29 (1141.46, 1967.15) | 2144.96 (1489.46, 2996.49) |
| Croatia | 9671.48 (8407.16, 10925.21) | 13607.61 (11093.21, 16192.75) | 18222 (14510.58, 22211.24) | 23299.5 (17352.61, 29991.74) |
| Cyprus | 1239.24 (1007.73, 1505.11) | 1486.73 (1172.65, 1897.28) | 1726.15 (1299.28, 2283.65) | 1975.63 (1421.93, 2713.44) |
| Czechia | 22944.87 (20005.5, 25769.56) | 29411.54 (24522.43, 34980.93) | 38292.29 (30705.94, 46995.44) | 47232.52 (36339.73, 60458.3) |
| Democratic Republic of the Congo | 278.44 (169.67, 488.49) | 576.47 (297.76, 1116.07) | 1201.7 (620.51, 2290.39) | 2453.66 (1251.78, 4834.32) |
| Denmark | 21604.22 (19680.98, 23535.3) | 27102.85 (23158.85, 31183.69) | 31660.1 (26433.33, 36816.08) | 36928.93 (29761.2, 43859.38) |
| Djibouti | 32.39 (20.96, 47.65) | 60.89 (38.19, 92.78) | 111.47 (69.46, 167.32) | 189.79 (115.9, 284.13) |
| Dominican Republic | 2160.66 (1461.41, 2938.02) | 3969.03 (2526.57, 5496.05) | 7324.49 (4426.48, 10456.96) | 13059.08 (7276.13, 19806.3) |
| Ecuador | 1014.12 (790, 1289.43) | 1242.33 (913.95, 1625.85) | 1465.83 (1039.32, 2000.29) | 1672.4 (1115.26, 2406.61) |
| Egypt | 9673.73 (7516.76, 12109.08) | 13695.98 (10547.17, 17243.03) | 18745.55 (13578.26, 25177.35) | 23948.1 (15758.19, 35111.14) |
| El Salvador | 400.73 (319.11, 504.95) | 604.07 (441.31, 826.46) | 891.9 (625.29, 1270.16) | 1266.47 (815.33, 1905.48) |
| Equatorial Guinea | 79.98 (50.95, 116.41) | 57.69 (37.88, 84.37) | 42.6 (27.39, 62.1) | 30.42 (18.62, 45.46) |
| Estonia | 2341.67 (1978.41, 2655.36) | 3076.32 (2498.63, 3719.02) | 4040.83 (3099.29, 5148.65) | 5302.66 (3833.62, 7198.85) |
| Ethiopia | 605.45 (482.17, 755.92) | 1490.17 (1119.97, 1952.1) | 3960.09 (2887.68, 5239.03) | 10403.14 (7212.9, 14079.92) |
| Fiji | 71.71 (54.18, 93.5) | 98.46 (72.06, 126.85) | 127.24 (89.32, 166.14) | 157.67 (103.81, 222.15) |
| Finland | 10661.51 (9630.09, 11653.59) | 11719.75 (10018.9, 13423.33) | 12080.98 (10115.8, 14356.06) | 12429.38 (9923.64, 15141.97) |
| France | 163446.59 (149071.53, 177668.29) | 189857.78 (163086.16, 219572.91) | 205934.2 (171643.97, 240717.3) | 215658.28 (176105.46, 258028.88) |
| Gabon | 183.74 (128.94, 245.32) | 192.16 (130.09, 261.08) | 192.66 (129.59, 263.5) | 193.34 (127.08, 273.95) |
| Gambia | 5.3 (3.74, 7.24) | 6.18 (4.18, 8.49) | 7.3 (4.85, 10.17) | 8.79 (5.62, 12.65) |
| Georgia | 2365.79 (2030.53, 2730.25) | 4066.63 (3281.36, 5009.64) | 6057.82 (4653.08, 7890.61) | 8225.88 (5875.58, 11494.57) |
| Germany | 247803.69 (227658.05, 267698.94) | 280437.81 (241945.33, 317866.78) | 287019.28 (241227.58, 330111.71) | 288502.76 (238902.27, 338135.12) |
| Ghana | 438.83 (320.11, 556.92) | 601 (447.42, 791.96) | 839.6 (610.85, 1118.24) | 1162.79 (809.22, 1590.88) |
| Greece | 20584.65 (19120.16, 22003) | 20963.86 (17584.23, 24394.33) | 20000.75 (16506.9, 24050.89) | 17566.41 (13774.89, 21663.01) |
| Grenada | 16.29 (13.72, 18.92) | 22.24 (17.49, 27.58) | 28.01 (20.62, 37.32) | 33.95 (22.15, 49.67) |
| Guatemala | 451.98 (380.43, 527.81) | 612.09 (496.7, 745.65) | 839.4 (633.06, 1072.81) | 1137.31 (774.79, 1565.15) |
| Guinea | 89.74 (62.8, 127.06) | 133.67 (96.08, 186.26) | 213.98 (150.65, 302.21) | 347.56 (230.32, 502.61) |
| Guinea-Bissau | 11.04 (8.09, 14.71) | 18.53 (10.68, 25.55) | 34.67 (22.93, 49.62) | 64.01 (42.21, 94.83) |
| Guyana | 111.98 (82.87, 145.31) | 253.82 (186.77, 337.79) | 526.68 (365.91, 726.19) | 1051.49 (665.26, 1569.7) |
| Haiti | 226.52 (137.93, 357.6) | 259.85 (165.72, 401.35) | 296.39 (178.33, 479.37) | 327.05 (175.26, 564.4) |
| Honduras | 530.36 (367.62, 756.49) | 853.09 (550.95, 1306.22) | 1425.77 (863.57, 2270.74) | 2371.61 (1344.78, 4052.93) |
| Hungary | 30240.96 (25864.95, 34709.1) | 38427.31 (30440.91, 46500.17) | 47588.96 (36694.45, 61216.04) | 55151.31 (40090, 72609.52) |
| Iceland | 588.45 (524.93, 652.68) | 797.94 (676.03, 925.43) | 1062.13 (868.43, 1256.15) | 1390.96 (1066.17, 1700.65) |
| India | 48186.97 (39643.9, 55799.01) | 74087.61 (58340.1, 91287.74) | 109728.57 (79476.52, 142130.83) | 154315.74 (100696.35, 218881.2) |
| Indonesia | 574773.18 (390015.36, 753064.32) | 832340.47 (572200.89, 1078797.76) | 1117006.62 (733760.59, 1479241.31) | 1406300.9 (889341.41, 1943026.14) |
| Iran (Islamic Republic of) | 10588.35 (9628.81, 11639.25) | 12041.73 (9985.25, 14317.8) | 12835.92 (9600.39, 16302.55) | 12567.03 (8374.78, 17401.45) |
| Iraq | 2754.48 (2001.99, 3539.9) | 2261.21 (1352.13, 3219.44) | 1746.45 (999.65, 2655.65) | 1246.17 (673.37, 2052.16) |
| Ireland | 11119.79 (9963.05, 12289.78) | 21251.11 (17798.76, 24388.25) | 41192.49 (34309.96, 48123.9) | 77228.4 (63024.55, 93114.91) |
| Israel | 5805.62 (5248.37, 6341.19) | 7182.7 (5782.99, 8533.62) | 8659.46 (6886.45, 10680.27) | 10102.66 (7709.54, 12956.19) |
| Italy | 137977.91 (127318.02, 145710.92) | 152860.08 (132848.24, 172151.75) | 159023.33 (133687.54, 184529.05) | 154037.21 (125039.68, 185728.04) |
| Jamaica | 380.6 (289.53, 498.55) | 460.64 (335.87, 612.75) | 523.07 (358.83, 725.75) | 571.3 (357.8, 846.23) |
| Japan | 261982.55 (233713.61, 277261.84) | 249150.57 (208763.25, 280904.58) | 216365.2 (176445.92, 250577.18) | 183002.62 (144351.78, 222423.93) |
| Jordan | 585.9 (444.59, 780.06) | 715.97 (529.56, 980.7) | 820.68 (572.41, 1176.22) | 879.17 (566.86, 1309.6) |
| Kazakhstan | 6366.2 (5468.58, 7391.24) | 7767.18 (6228.75, 9496.09) | 8973.52 (6620.95, 11807.39) | 9815.54 (6492.53, 14124.35) |
| Kenya | 318.41 (248.68, 406.13) | 696.38 (546.65, 916.58) | 1620.29 (1211.51, 2177.57) | 3686.75 (2588.15, 5184.71) |
| Kiribati | 4.73 (3.42, 6.4) | 6.98 (5.15, 9.46) | 9.92 (7.14, 13.4) | 13.99 (9.38, 20.07) |
| Kuwait | 1123.9 (913.87, 1381.38) | 1174.97 (890.72, 1508.11) | 1119.99 (800.74, 1499.54) | 918.62 (589.05, 1314.06) |
| Lao People's Democratic Republic | 650.58 (470.92, 906.44) | 1284.02 (932.48, 1784.9) | 2556.02 (1830.28, 3613.21) | 4978.65 (3384.43, 7425.83) |
| Latvia | 3011.2 (2545.49, 3440.34) | 3778.98 (3117.27, 4518.21) | 4581.57 (3590.73, 5728.59) | 5367.54 (3913.83, 7036.98) |
| Lebanon | 1262.64 (993.24, 1600.55) | 1060.3 (796.28, 1416.89) | 942.86 (683.81, 1328.2) | 799.07 (532.13, 1175.54) |
| Lesotho | 90.91 (63.52, 128.29) | 89.02 (60.8, 123.23) | 98.8 (66.08, 139.35) | 113.45 (73.98, 165.94) |
| Liberia | 12.72 (7.99, 20.14) | 21.66 (13.24, 33.84) | 36.85 (22.41, 58.3) | 58.07 (34.21, 94.08) |
| Libya | 2277.5 (1618.16, 3160.99) | 2437.63 (1672.35, 3641.21) | 2342.83 (1551.26, 3544.31) | 1873.15 (1133.34, 2895) |
| Lithuania | 5419.03 (4592.38, 6244.25) | 8167.55 (6819.71, 9599.02) | 10971.08 (8683.92, 13664.51) | 14069.4 (10094.38, 18748.43) |
| Luxembourg | 1830.87 (1645.14, 2027.73) | 2231.12 (1912.47, 2563.34) | 2481.2 (2071.08, 2906.69) | 2615.98 (2113.68, 3112.85) |
| Madagascar | 83.34 (60.27, 111.55) | 102.4 (73.56, 136.57) | 124.54 (87.52, 168.45) | 146.31 (100.71, 207.36) |
| Malawi | 25.56 (18.98, 34.33) | 29.92 (21.75, 40.76) | 37.04 (25.69, 51.97) | 45.42 (29.64, 64.81) |
| Malaysia | 11373.29 (9578.24, 13195.58) | 15746.48 (12732.87, 19537.33) | 20956.6 (16255.7, 26987.27) | 26874.25 (19331.94, 36741.89) |
| Maldives | 32.68 (25.25, 40.48) | 70.28 (51.61, 89.83) | 164.47 (120.88, 214.69) | 361.06 (256.48, 490.84) |
| Mali | 66.02 (49.02, 88.3) | 100.9 (72.84, 137.58) | 160.17 (110.17, 223.09) | 263.24 (176.54, 378.19) |
| Malta | 591.92 (525.15, 666.36) | 823.19 (670.13, 987.43) | 1113.29 (880.18, 1366.83) | 1470.99 (1104.86, 1857.47) |
| Mauritania | 58.05 (41.87, 82.01) | 81.12 (58.14, 111.32) | 118.04 (81.98, 161.96) | 174.28 (116.01, 243.1) |
| Mauritius | 436.37 (399.4, 471.21) | 576.27 (487.59, 675.78) | 680.84 (551.67, 828.31) | 764.55 (586.53, 969.29) |
| Mexico | 13196.49 (11653.96, 14790.86) | 15381.43 (13107.2, 17954.59) | 16599.01 (13095.45, 20471.89) | 16805.47 (12105.53, 22495.54) |
| Micronesia (Federated States of) | 8.86 (6.08, 12.16) | 10.54 (7.28, 14.77) | 11.25 (7.64, 15.91) | 11.7 (7.27, 17.34) |
| Mongolia | 566.51 (440.39, 724.97) | 734.74 (552.81, 938.16) | 873.6 (609.97, 1164.71) | 956.94 (583.92, 1422.3) |
| Montenegro | 1057.03 (833, 1307.13) | 1507.25 (1116.19, 1937.21) | 2051.29 (1504.03, 2724.61) | 2680.69 (1821.71, 3718.58) |
| Morocco | 3393.08 (2468.68, 4312.58) | 4253.93 (3092.27, 5619.82) | 4982.04 (3367.76, 6862.54) | 5445.59 (3309.91, 8381.34) |
| Mozambique | 76.09 (55.6, 98.28) | 98.03 (73.07, 129.55) | 133.46 (94.45, 180.97) | 180.89 (122.46, 254.1) |
| Myanmar | 3424.16 (2506.41, 4515.04) | 4033.05 (2943.01, 5313.46) | 4436.21 (3191.05, 5964.61) | 4734.83 (3250.48, 6761.69) |
| Namibia | 75.41 (54.39, 100.7) | 80.48 (58.68, 107.75) | 88.03 (64.21, 120.39) | 94.2 (67.24, 132.44) |
| Nepal | 446.15 (329.29, 621.77) | 744.17 (520.18, 1049.54) | 1241.44 (850.01, 1838.58) | 2041.94 (1308.11, 3264.83) |
| Netherlands | 58496.5 (53390.69, 62682.23) | 69426.81 (59544.99, 79569.51) | 74060.12 (62030.75, 86536.86) | 76077.06 (62067.96, 91085.63) |
| New Zealand | 6645.79 (6100.56, 7234.61) | 9169.82 (7873.31, 10606.65) | 12039.97 (10052.22, 14255.34) | 15545.42 (12411.46, 19187.66) |
| Nicaragua | 101.76 (81.38, 126.51) | 166.38 (124.2, 221.43) | 257.38 (183.38, 355.55) | 373.04 (249.89, 543.28) |
| Niger | 31.38 (21.2, 53.66) | 39.17 (26.31, 66.16) | 49 (31.58, 81.38) | 67.39 (41.31, 113.02) |
| Nigeria | 504.72 (389.92, 651.45) | 657.93 (489.16, 858.37) | 853.61 (630.38, 1129.14) | 1094.06 (768.45, 1505.44) |
| North Macedonia | 1925.87 (1459.06, 2417.35) | 2608.62 (1800.9, 3557.67) | 3520.86 (2334.36, 4921.66) | 4406.15 (2683.38, 6532.81) |
| Norway | 13611.58 (12486.82, 14367.04) | 15209.17 (13397.31, 17043.02) | 15799.71 (13580.23, 17858.5) | 16046.12 (13554.89, 18334.06) |
| Oman | 257.07 (189.35, 354.14) | 293.98 (210.34, 408.89) | 307.58 (206.97, 441.06) | 280.02 (166.7, 426.3) |
| Pakistan | 7500.73 (5619.3, 9856.52) | 10712.15 (7814.25, 14445.45) | 15093.81 (10290.61, 21298.42) | 20353.19 (12950.33, 31276.17) |
| Panama | 732.36 (563.2, 891.46) | 1359.6 (1027.04, 1729.65) | 2498.07 (1789.94, 3297.39) | 4388.73 (2953.63, 6322.22) |
| Papua New Guinea | 299.3 (194.94, 470.21) | 462.96 (291.64, 707) | 697.26 (426.28, 1095.54) | 1006.02 (572.79, 1688.79) |
| Paraguay | 979.56 (739.22, 1296.85) | 1453.03 (1025.95, 2035.85) | 2036.5 (1311.84, 2867.69) | 2706.92 (1560.6, 4086.01) |
| Peru | 3146.37 (2272.91, 4024.8) | 4035.48 (2926.82, 5381.38) | 5208.72 (3647.3, 7058.48) | 6464.59 (4341.92, 9312.38) |
| Philippines | 10260.57 (8401.45, 12289.2) | 13859.45 (11001.52, 16856.2) | 18012.8 (14192.36, 22298.39) | 22519.16 (16929.85, 28731.25) |
| Poland | 100487.35 (91362.22, 109247.48) | 138363.87 (117594.97, 160118.77) | 191250.19 (158112.09, 227544.2) | 254813.9 (198021.63, 318495.53) |
| Portugal | 13916.63 (12724.93, 15094.83) | 16428.23 (13683.63, 19231.07) | 18419.57 (14956.69, 22169.33) | 20122.01 (15899.71, 25006.57) |
| Qatar | 1112.45 (811.92, 1583.37) | 1533.3 (1087.52, 2137.29) | 1662.09 (1138.82, 2380.82) | 1528.2 (987.04, 2288.29) |
| Republic of Korea | 74589.16 (62812.1, 87369.32) | 99433.62 (83219.51, 121241.39) | 128711.09 (103632.58, 160689.91) | 149255.63 (112739.98, 193089.9) |
| Republic of Moldova | 1750.07 (1535.47, 1989.83) | 2882.08 (2196.93, 3609) | 4670.32 (3254.84, 6275.22) | 6887.19 (4261.5, 9941.8) |
| Romania | 41938.05 (36737.25, 47532.85) | 60901.54 (48967.55, 75044.94) | 84625.88 (63769.31, 110002.89) | 107927.34 (76298.7, 148374.55) |
| Russian Federation | 177457.46 (160025.27, 193582.44) | 204251.42 (171875.44, 241335.36) | 219680.43 (176221.38, 272685.76) | 232606.7 (170766.37, 305128.47) |
| Rwanda | 86.95 (58.02, 121.99) | 169.85 (111.47, 249.09) | 338.9 (218.66, 489.3) | 673.91 (419.2, 994.15) |
| Saint Lucia | 30.07 (24.17, 36.74) | 31.58 (23.61, 41.36) | 30.72 (21.21, 41.68) | 28.3 (17.26, 42.34) |
| Saint Vincent and the Grenadines | 17.21 (15.07, 19.72) | 21.92 (17.72, 27.28) | 25.65 (18.64, 33.92) | 28.52 (18.14, 41.52) |
| Samoa | 6.01 (4.38, 7.92) | 6.7 (4.86, 9.15) | 6.92 (4.92, 9.72) | 7.04 (4.74, 10.29) |
| Sao Tome and Principe | 5.03 (3.71, 6.83) | 8.17 (5.85, 11.2) | 13.29 (9.54, 18.6) | 20.32 (13.85, 29.47) |
| Saudi Arabia | 6107.11 (4675.53, 7718.79) | 8908.27 (6453.24, 12173.97) | 10611.38 (7354.62, 14808.97) | 10602.24 (6771.39, 16097.32) |
| Senegal | 164.64 (121.97, 221.96) | 242.04 (180.28, 322.53) | 367.49 (271.48, 489.55) | 563.64 (402.03, 761.45) |
| Serbia | 14601.09 (11498.38, 18045.74) | 17988.32 (13410.48, 22800.55) | 22110.32 (15977.62, 29844.04) | 26485.45 (18182.21, 36688.19) |
| Seychelles | 41.7 (34.89, 49.34) | 59.38 (47.98, 73.22) | 77.54 (61.08, 97.57) | 92.44 (68.67, 120.49) |
| Sierra Leone | 31.54 (21.87, 44.11) | 49.97 (34.03, 69.89) | 82.39 (56.03, 117.82) | 134.55 (87.17, 204.36) |
| Singapore | 9969.95 (8956.39, 11174.48) | 13670.41 (11686.93, 15936.15) | 17559.92 (14261.63, 20970.15) | 20735.37 (15971.26, 25749.11) |
| Slovakia | 7209.93 (5618.11, 8788.6) | 7730.13 (5934.24, 9819.92) | 7844.69 (5795.69, 10251.96) | 7448.76 (5281.1, 9898.74) |
| Slovenia | 4378.29 (3705, 5040.53) | 5598.93 (4567.72, 6665.76) | 6835.14 (5374.61, 8604.39) | 7954.18 (5985.47, 10422.69) |
| Solomon Islands | 18.51 (13.58, 25.41) | 23.86 (17.44, 31.75) | 29.48 (21.04, 40.26) | 35.01 (23.48, 50.77) |
| Somalia | 50.32 (30.46, 96.76) | 94.29 (50.38, 195.49) | 188.98 (96.52, 393.84) | 359.53 (161.61, 798.95) |
| South Africa | 13106.77 (11774.42, 14642.7) | 13903.29 (11787.14, 16291.16) | 14357.64 (12078.08, 17168.76) | 13909.94 (11111.32, 17064.2) |
| Spain | 77003.46 (70789.39, 82890.14) | 84872.55 (73498.24, 96674.45) | 86186.54 (72335.57, 101338.1) | 82430.52 (67599.65, 100977.32) |
| Sri Lanka | 2600.81 (1664.28, 3610.78) | 3413.62 (2273.5, 4704.37) | 4127.45 (2743.97, 5696.9) | 4737.13 (2994.34, 6721.15) |
| Sudan | 504.14 (333.2, 748.89) | 580.66 (380.02, 846.57) | 675.5 (439.24, 1017.99) | 761.41 (441.76, 1223.04) |
| Suriname | 105.21 (79.12, 137.71) | 102.56 (74.09, 136.78) | 90.85 (61.01, 126.76) | 76.81 (46.46, 115.57) |
| Sweden | 15277.57 (13256.92, 17270.72) | 17843.22 (14777.17, 20925.58) | 19736.28 (16226.93, 23608.98) | 21966.54 (17391.65, 26994.33) |
| Switzerland | 20302.96 (18250.25, 21779.86) | 22289.03 (19012.25, 25548.4) | 22698.7 (18965.74, 26212.62) | 22124 (18121.4, 25822.28) |
| Tajikistan | 230.06 (155.12, 333.88) | 370.78 (238.54, 549.02) | 564.96 (342.33, 902.04) | 833.9 (474.98, 1437.51) |
| Thailand | 39966.63 (30393.41, 50084.32) | 47948.11 (35855.3, 63334.72) | 52130.97 (37941.19, 68522.22) | 52272.54 (36684.31, 71061.85) |
| Timor-Leste | 52.52 (38.77, 68.94) | 42.61 (31.73, 56.55) | 31.97 (23.12, 43.27) | 25.22 (16.8, 35.2) |
| Togo | 55.34 (34.87, 77.05) | 84.26 (54.26, 118.45) | 125.38 (80.48, 181.86) | 179.81 (111.83, 269.38) |
| Tonga | 12.76 (9.72, 16.33) | 16.47 (12.41, 21.23) | 20.91 (15.27, 27.71) | 25.78 (17.35, 35.77) |
| Tunisia | 2227.96 (1481.95, 3157.48) | 2436.62 (1660.34, 3472.99) | 2469.07 (1651.91, 3568.93) | 2283.18 (1406.72, 3475.99) |
| Uganda | 204.41 (147.35, 274.53) | 285.92 (201.2, 384.93) | 409.55 (279.12, 559.56) | 587.32 (386.03, 827.32) |
| Ukraine | 21781.75 (15133.2, 29679.81) | 24883.51 (17207.19, 34163.32) | 27079.83 (17569.71, 39433.39) | 27419.31 (16514.16, 42576.31) |
| United Arab Emirates | 3326.68 (2459.06, 5220.4) | 7222.99 (5123.59, 10943.75) | 11461.62 (7774.39, 17109.86) | 14621.54 (9368.82, 22577.98) |
| United Kingdom | 125727.62 (117916.76, 130360.42) | 147829.11 (131819.4, 161807.12) | 164459.63 (142970.61, 186815.45) | 178303.66 (147993.88, 213580.76) |
| United Republic of Tanzania | 378.74 (279.05, 515.65) | 576.85 (404.22, 785.14) | 859.82 (595.39, 1192.58) | 1269.73 (855.51, 1814.14) |
| United States of America | 903283.29 (844856.87, 942827.12) | 1068186.66 (945656.13, 1169220.02) | 1127918.43 (969453.17, 1284772.82) | 1142634.98 (947631.96, 1352634.92) |
| Uruguay | 3095.17 (2857.62, 3319.29) | 3339.84 (2760.33, 3893.1) | 3512.96 (2856.6, 4181.53) | 3674.01 (2921.67, 4513.11) |
| Uzbekistan | 1556.05 (1266.73, 1876.03) | 2283.55 (1569.85, 3093.36) | 3123.43 (1984.47, 4580.02) | 4090.06 (2325.28, 6521.82) |
| Vanuatu | 9.11 (5.98, 14.37) | 12.55 (8.5, 19.93) | 17.26 (11.48, 27.64) | 23.48 (14.32, 39.76) |
| Viet Nam | 23030 (16969.34, 28944) | 42898.47 (31956.12, 54016.49) | 73679.79 (54164.88, 95123.79) | 116991.54 (83043.3, 158082.73) |
| Zambia | 239.33 (162.74, 425.12) | 290.75 (201.54, 530.47) | 363.99 (254.82, 617.12) | 446.58 (305.08, 725.52) |
| Zimbabwe | 198.66 (150.69, 264.05) | 218.53 (160.46, 289.54) | 253.16 (188, 343.95) | 289.3 (205.29, 407.27) |

Note: Data were available for 169 countries covering more than 99% of the world population. All future year estimates were discounted at a 3% annual discount rate.

**Supplementary Table 19** Estimated economic burden of tracheal, bronchus, and lung cancer by country through 2050 by sex, millions of 2023 US$. (E=1.5)

| Location | economic burden 2021 95%UI | |  | economic burden 2050 95%UI | |
| --- | --- | --- | --- | --- | --- |
|  | Female | Male |  | Female | Male |
| Afghanistan | 10.48 (5.82, 15.67) | 13.81 (7.94, 26.03) |  | 11.31 (5.07, 20.49) | 13.71 (5.56, 28.86) |
| Albania | 148.86 (104.39, 203.41) | 473.96 (329.86, 641.3) |  | 395.87 (231.52, 604.86) | 910.78 (539.38, 1373.2) |
| Algeria | 158.9 (109.29, 215.32) | 683.63 (514.56, 902.97) |  | 163.06 (98.24, 249.73) | 518.21 (306.14, 770.43) |
| Angola | 51.79 (34.04, 75.95) | 156.63 (110.32, 209.53) |  | 62.54 (39.05, 97.49) | 141.48 (96.32, 204.87) |
| Argentina | 4102.44 (3640.88, 4570.64) | 7956.21 (7180.18, 8753.53) |  | 7428.38 (5679.14, 9661.44) | 11389.1 (8841.94, 14598.61) |
| Armenia | 115.04 (99.78, 131.83) | 573.73 (505.16, 652.44) |  | 340.72 (232.96, 490.53) | 1205.23 (798.02, 1764.35) |
| Australia | 13348.67 (11895.17, 14519.39) | 19598.73 (17862.16, 21511.83) |  | 25785.24 (20124.67, 31987.86) | 25756.57 (20221.41, 31296.94) |
| Austria | 7598.01 (6828.61, 8291.33) | 11117.18 (10225.79, 12213.38) |  | 9412.69 (7602.97, 11687.89) | 11357.84 (9016.01, 13948.07) |
| Azerbaijan | 243.38 (177.34, 328.16) | 978.32 (608.55, 1342.74) |  | 287.69 (181.76, 437.98) | 794.98 (412.87, 1360.81) |
| Bahamas | 35.32 (28.35, 44.19) | 77.75 (61.73, 99.53) |  | 50.45 (33.74, 70.19) | 76.57 (47.49, 111.06) |
| Bahrain | 161.41 (124.14, 204.56) | 461.1 (346.83, 616.56) |  | 306.35 (196.97, 476.25) | 1105.74 (674.34, 1669.7) |
| Bangladesh | 375.59 (241.16, 569.29) | 946.98 (677.24, 1401.86) |  | 3167.33 (1837.41, 5306.05) | 4948.89 (2958.47, 7978.01) |
| Barbados | 9.48 (7.33, 12.01) | 16.63 (12.49, 21.18) |  | 5.91 (3.78, 8.45) | 6.25 (3.7, 9.38) |
| Belarus | 579.77 (452.8, 710.89) | 4026.87 (3103.45, 4966.24) |  | 628.88 (406.54, 895.06) | 3181.41 (2036.57, 4721.13) |
| Belgium | 9990.52 (8972.11, 10844.56) | 20995.09 (18985.54, 23072.74) |  | 15403.16 (12274.04, 19105.48) | 24664.86 (19486.61, 31266.39) |
| Belize | 3.04 (2.62, 3.5) | 6.58 (5.54, 7.68) |  | 7.14 (4.89, 10.06) | 10.11 (6.14, 15.21) |
| Benin | 5.3 (3.56, 7.66) | 13.94 (10.39, 18.67) |  | 37.13 (24.33, 56.09) | 58.76 (39.76, 80.74) |
| Bhutan | 4.52 (2.63, 7.01) | 8.53 (5.28, 12.6) |  | 28.24 (14.72, 48.37) | 44.43 (24.42, 76.33) |
| Bolivia (Plurinational State of) | 114.98 (66.4, 170.98) | 147.15 (103.31, 205.33) |  | 606.86 (312.17, 990.63) | 640.28 (390.19, 1033.24) |
| Bosnia and Herzegovina | 458.65 (358.18, 582.43) | 1474.72 (1129.26, 1879.65) |  | 1347.23 (865.37, 1959.54) | 3019.93 (1956.98, 4432.76) |
| Botswana | 49.01 (34.27, 67.24) | 117.71 (77.27, 158.14) |  | 181.34 (119.22, 264.06) | 314.39 (198.32, 437.16) |
| Brazil | 9621.13 (8943.92, 10204.27) | 12447.78 (11702.56, 13111.31) |  | 9695.59 (6653.86, 13440.36) | 10198.73 (6566.3, 15257.44) |
| Brunei Darussalam | 206.39 (163.8, 253.68) | 255.03 (200.17, 318.63) |  | 153.55 (107.75, 213.44) | 149.27 (105.24, 208.66) |
| Bulgaria | 2013.27 (1657.24, 2393.6) | 6242.02 (5213.14, 7299.12) |  | 4357.26 (3154.88, 5826.61) | 9549.04 (6730.38, 13247.82) |
| Burkina Faso | 4.11 (2.6, 6.15) | 12.43 (9.05, 18.8) |  | 26.97 (16.16, 43.17) | 50.8 (34.27, 74.35) |
| Burundi | 0.45 (0.28, 0.67) | 1.49 (1.03, 2.26) |  | 1.59 (0.95, 2.59) | 3.26 (2.05, 5.2) |
| Cabo Verde | 3.7 (2.76, 4.99) | 8.37 (6.19, 10.5) |  | 9.66 (6.55, 13.72) | 17.01 (11.45, 23.07) |
| Cambodia | 85.76 (59.16, 117.52) | 219.86 (154.35, 298.16) |  | 475.52 (302.45, 715.78) | 981.15 (619.99, 1415.51) |
| Cameroon | 20.89 (13.53, 30.15) | 56.38 (37.24, 80.02) |  | 92.02 (57.36, 140.49) | 161 (106.65, 230.38) |
| Canada | 33460.84 (30287.09, 36388.82) | 37294.83 (34290.33, 40509.68) |  | 50518.99 (40959.24, 62796.75) | 38915.23 (31516.88, 47075.84) |
| Central African Republic | 0.51 (0.36, 0.71) | 2.17 (1.22, 4.47) |  | 0.73 (0.44, 1.1) | 1.73 (0.81, 3.98) |
| Chad | 1.5 (0.94, 2.13) | 5.66 (3.92, 8.48) |  | 2.61 (1.55, 4.05) | 4.67 (2.92, 7.05) |
| Chile | 1679.24 (1507.77, 1855.73) | 2441.44 (2208.54, 2661.59) |  | 3626.22 (2863.61, 4480.48) | 3842.86 (3072.84, 4581.94) |
| China | 221399.68 (172917.1, 276617) | 466625.94 (340087.81, 608407.28) |  | 994494.64 (684555.14, 1394041.7) | 1494584.43 (1025371.69, 1997374.34) |
| Colombia | 1467.44 (1232.87, 1728.41) | 1939.97 (1563.46, 2354.79) |  | 4810.22 (3306.41, 6561.58) | 4863.76 (3084.95, 6905.62) |
| Comoros | 0.79 (0.47, 1.17) | 1.48 (0.84, 2.21) |  | 2.26 (1.25, 3.49) | 3.36 (1.72, 5.2) |
| Congo | 8.83 (5.18, 13.73) | 19.58 (13.96, 26.96) |  | 4.01 (2.12, 6.48) | 7.28 (4.85, 10.74) |
| Costa Rica | 130.97 (114.12, 148.74) | 217.49 (188.2, 251.19) |  | 542.08 (379.39, 757.71) | 630.87 (422.76, 894.5) |
| Croatia | 1904.78 (1584.14, 2279.36) | 4923.89 (4213.96, 5672.75) |  | 5155.45 (3706.71, 7028.14) | 11295.45 (8234.79, 14705.75) |
| Cyprus | 214.91 (162.56, 263.44) | 738.03 (589.99, 893.33) |  | 464.5 (315.66, 644.65) | 1054.7 (752.95, 1453.34) |
| Czechia | 6126.6 (5291.6, 6983.12) | 11872.36 (10339.92, 13535.71) |  | 14091.92 (10321.94, 18602.64) | 22959.34 (17221.35, 29817.02) |
| Democratic Republic of the Congo | 9.51 (5.71, 14.48) | 25.74 (13.83, 50.69) |  | 103.04 (53.67, 169.37) | 207.59 (92.56, 463.72) |
| Denmark | 10369.65 (9234.82, 11309.2) | 10862.33 (9817.07, 12026.63) |  | 20729.4 (16126.7, 24950.32) | 15563.24 (12397.42, 18976.31) |
| Djibouti | 2.02 (1.32, 2.9) | 6.99 (4.11, 10.66) |  | 17.25 (11.05, 26.44) | 35.53 (19.61, 55.69) |
| Dominican Republic | 403.16 (279.59, 557.24) | 729.01 (479.57, 1011.1) |  | 2813.99 (1639.58, 4170.32) | 4028.87 (2194.35, 6456.31) |
| Ecuador | 182.69 (144.02, 229.33) | 226.67 (170.98, 295.2) |  | 353.19 (241.44, 501.22) | 321.89 (202.13, 480.84) |
| Egypt | 1051.13 (830.62, 1301.67) | 3092.42 (2406.38, 3936.4) |  | 3233.1 (2136.05, 4624.58) | 7024.6 (4516.25, 10174.77) |
| El Salvador | 72.21 (56.26, 93.02) | 75.01 (58.67, 96.44) |  | 271.15 (176.63, 407.43) | 194.12 (117.85, 297.25) |
| Equatorial Guinea | 11.29 (6.23, 18.69) | 21.94 (14.26, 31.82) |  | 4.47 (2.5, 7.41) | 8.17 (5.08, 12.07) |
| Estonia | 419.31 (353.17, 484.37) | 1388.84 (1175.97, 1587.09) |  | 1146.45 (798.85, 1601.72) | 2948.06 (2098.43, 4078.6) |
| Ethiopia | 23 (18.02, 29.2) | 91.64 (69.14, 117.63) |  | 509.35 (343.16, 713.51) | 1460.39 (994.91, 2048.09) |
| Fiji | 10.11 (7.44, 13.29) | 17.82 (13.15, 23.61) |  | 24.74 (15.63, 35.84) | 36.67 (24.52, 51.91) |
| Finland | 3309.33 (2924.05, 3653.44) | 6119.26 (5447.23, 6799.31) |  | 4649.85 (3575.66, 5706.09) | 6342.17 (5023.64, 7964.44) |
| France | 41467.31 (36457.36, 46072.87) | 98966.11 (89566.64, 108178.99) |  | 64532.05 (50667.47, 79709.99) | 120761.69 (98158.68, 148147.83) |
| Gabon | 19.9 (13.02, 29.39) | 58.22 (41.01, 80.76) |  | 29.33 (17.42, 44.41) | 52.88 (34.88, 74.8) |
| Gambia | 0.21 (0.12, 0.33) | 0.73 (0.52, 1.01) |  | 0.51 (0.29, 0.82) | 1.04 (0.66, 1.52) |
| Georgia | 153.9 (132.26, 179.19) | 968.85 (834.13, 1115.66) |  | 464.55 (332.58, 649.57) | 3439.25 (2421.62, 4825.68) |
| Germany | 85375.18 (77670.72, 91891.24) | 144725.48 (132976.84, 157126.73) |  | 115531.98 (92086.33, 139143.64) | 152360.2 (126188.93, 181168.75) |
| Ghana | 28.43 (21.16, 37.79) | 96.88 (69.27, 124.95) |  | 90.07 (59.8, 131.88) | 241.97 (163.67, 337.83) |
| Greece | 3086.67 (2788, 3346.37) | 10555.16 (9811.89, 11271.74) |  | 3331.7 (2563.57, 4234.57) | 8309.89 (6434.77, 10430.77) |
| Grenada | 2.3 (1.96, 2.65) | 4.91 (4.11, 5.76) |  | 6.23 (4.28, 8.77) | 8.79 (5.45, 13.55) |
| Guatemala | 82.23 (70.01, 96.1) | 83.9 (70.22, 99.97) |  | 242.24 (172.37, 332.02) | 175.8 (110.93, 250.68) |
| Guinea | 3.23 (1.85, 4.68) | 13.6 (9.43, 19.32) |  | 20.24 (11.95, 30.91) | 44.92 (29.42, 67.02) |
| Guinea-Bissau | 0.51 (0.36, 0.71) | 1.35 (0.94, 1.94) |  | 3.8 (2.34, 5.66) | 6.99 (4.43, 11.05) |
| Guyana | 25.16 (18.16, 33.89) | 38.32 (27.84, 50.41) |  | 294.15 (185.78, 429) | 301.95 (183.96, 462.23) |
| Haiti | 15.91 (10.34, 23.55) | 31.98 (17.82, 57.28) |  | 27.52 (14.79, 48.32) | 41.63 (19.45, 84.42) |
| Honduras | 78.55 (47.07, 129.65) | 70.79 (53.19, 96.52) |  | 408.61 (210.46, 735.9) | 259.2 (151.21, 424.17) |
| Hungary | 8502.31 (7180.41, 9784.42) | 12882.78 (11098.76, 14778.62) |  | 17617.97 (12492.37, 23556.1) | 21382.64 (14945.24, 28604.41) |
| Iceland | 306.78 (270.3, 342.58) | 213.1 (188.58, 238.65) |  | 735.12 (573.15, 907.37) | 493.77 (389.03, 605.84) |
| India | 4725.64 (3925.66, 5655.81) | 10626.6 (8458.33, 12718.81) |  | 20162.97 (13137.34, 28962.05) | 29001.62 (18617.75, 41907.62) |
| Indonesia | 77876.19 (44378.69, 111777.52) | 165279.42 (112468.07, 222023.37) |  | 227329.43 (120686.14, 339289.69) | 367600.82 (244095.43, 503872.01) |
| Iran (Islamic Republic of) | 1605.52 (1349.42, 1836.3) | 3501.89 (3152.48, 3900.66) |  | 2327.24 (1513.56, 3369.28) | 3734.61 (2441.76, 5199.19) |
| Iraq | 273.89 (193.65, 373.53) | 738.03 (519.22, 944.35) |  | 150.08 (79.54, 238.27) | 307.74 (165.29, 495.12) |
| Ireland | 5404.44 (4627.56, 6202.71) | 6452.26 (5836.4, 7073.81) |  | 44554.9 (35059.3, 55688.26) | 37791.45 (31267.87, 45385.79) |
| Israel | 1466.54 (1294.02, 1614.64) | 3071.49 (2775.22, 3374.34) |  | 3094.65 (2269.21, 4003.49) | 4802.21 (3686.16, 6193.78) |
| Italy | 34969.49 (30627.65, 37909.59) | 78611.77 (73173.51, 82744.19) |  | 44716.75 (34956.01, 56963.71) | 82084.28 (66563.87, 99364.92) |
| Jamaica | 37.82 (28.6, 48.36) | 107.01 (79.57, 143.17) |  | 73.09 (45.99, 106.27) | 144.31 (88.75, 219.41) |
| Japan | 57758.16 (46574.69, 64045.44) | 150411.53 (139018.93, 156981.59) |  | 52535.42 (38115.34, 67744.51) | 92877.31 (74821.39, 112663.27) |
| Jordan | 46.74 (31.33, 65.01) | 176.22 (132.42, 235.95) |  | 83.6 (49.08, 131.86) | 250.95 (157.91, 371.17) |
| Kazakhstan | 719.99 (593.83, 852.93) | 3075.28 (2608.24, 3592.19) |  | 1512.8 (968.39, 2170.68) | 4338.82 (2770.06, 6327.25) |
| Kenya | 34.17 (22.8, 53.7) | 51.5 (39.4, 65.01) |  | 498.31 (318.33, 780.99) | 493.71 (357.11, 658.32) |
| Kiribati | 0.23 (0.15, 0.33) | 0.86 (0.63, 1.17) |  | 0.86 (0.5, 1.34) | 2.37 (1.55, 3.37) |
| Kuwait | 215 (182.45, 255.47) | 809.72 (641.17, 1016.08) |  | 196.73 (132.22, 271.28) | 640.83 (403.9, 932.01) |
| Lao People's Democratic Republic | 63.76 (41.41, 104.67) | 156.73 (108.69, 217.73) |  | 587.05 (341.28, 1020.95) | 1100.27 (746.44, 1596.4) |
| Latvia | 457.87 (382.05, 532.18) | 1627.67 (1359.21, 1855.12) |  | 1000.33 (719.97, 1361.37) | 2717.18 (1930.62, 3648.63) |
| Lebanon | 164.58 (111.12, 222.65) | 383.45 (287.13, 493.13) |  | 119.69 (72.06, 186.81) | 227.13 (144.02, 351.76) |
| Lesotho | 3.95 (2.21, 6.21) | 14.37 (9.58, 21.37) |  | 5.11 (2.68, 8.79) | 17.76 (11.64, 25.94) |
| Liberia | 0.53 (0.31, 0.76) | 1.3 (0.77, 2.24) |  | 3.08 (1.75, 4.81) | 5.27 (3.07, 8.54) |
| Libya | 156.98 (99.38, 230.48) | 1109.92 (771.32, 1552.83) |  | 157.1 (91.03, 256.82) | 884.87 (521.09, 1379.97) |
| Lithuania | 820 (690.4, 966.84) | 3362.39 (2853.11, 3864.54) |  | 2882.4 (2026.8, 3898.53) | 7976.32 (5695.89, 10782.2) |
| Luxembourg | 670.58 (596.8, 749.58) | 1390.93 (1231.1, 1552.59) |  | 1109.39 (867.99, 1340.38) | 1836.14 (1462.04, 2219.23) |
| Madagascar | 4.34 (2.91, 6.09) | 8.16 (5.74, 11.08) |  | 9.65 (6.35, 14.15) | 12.3 (8.3, 17.64) |
| Malawi | 0.69 (0.39, 1.04) | 3.15 (2.2, 4.44) |  | 1.59 (0.89, 2.53) | 5.25 (3.4, 7.79) |
| Malaysia | 2065.47 (1721.43, 2475.92) | 5114.72 (4226.14, 5929.58) |  | 6313.82 (4491.26, 8950.2) | 10652.46 (7659.35, 14509.69) |
| Maldives | 4.4 (3.38, 5.51) | 12.39 (9.11, 15.63) |  | 46.17 (31.94, 65.22) | 139.36 (95.48, 190.28) |
| Mali | 4.19 (2.89, 5.86) | 7.5 (5.06, 10.32) |  | 21.76 (14.28, 33.01) | 24.85 (15.27, 37.15) |
| Malta | 124.95 (106.95, 145.44) | 349.14 (312.21, 387.28) |  | 364.88 (269.51, 467.12) | 813.29 (608.03, 1039.6) |
| Mauritania | 5.64 (3.94, 7.82) | 10.62 (7.1, 15.99) |  | 21.93 (14.99, 31.99) | 26.89 (16.85, 39.63) |
| Mauritius | 74.02 (67.35, 80.75) | 181.19 (164.71, 196.5) |  | 163.35 (122.67, 211.59) | 283.79 (215.49, 362.4) |
| Mexico | 2753.77 (2332.66, 3183.59) | 4185.82 (3511.34, 4951.41) |  | 4335.56 (3151.1, 5731.62) | 4501.88 (3067.92, 6334.55) |
| Micronesia (Federated States of) | 0.59 (0.41, 0.81) | 1.52 (0.99, 2.28) |  | 0.97 (0.59, 1.47) | 1.82 (1.1, 2.86) |
| Mongolia | 46.89 (35.07, 59.9) | 175.89 (135.76, 227.97) |  | 98.8 (62.52, 145.99) | 277.52 (164.38, 416.63) |
| Montenegro | 156.61 (121.67, 194.31) | 454.82 (343.79, 574.59) |  | 504.83 (343.6, 733.69) | 1045.8 (695.03, 1485.21) |
| Morocco | 97.83 (66.84, 136.41) | 1094.27 (792.22, 1389.42) |  | 229.99 (136.23, 366.79) | 1683.24 (1013.5, 2577.4) |
| Mozambique | 3 (1.86, 4.23) | 7.01 (5.29, 9.11) |  | 9.58 (5.87, 14.62) | 14.21 (9.96, 19.61) |
| Myanmar | 309.11 (227.44, 431.15) | 549.27 (384.83, 746.14) |  | 494.25 (314.7, 716.22) | 692.69 (466.36, 1016.32) |
| Namibia | 10 (6.48, 14.32) | 18.12 (13.39, 23.75) |  | 14.78 (9.7, 22.54) | 20.35 (14.6, 27.86) |
| Nepal | 38.42 (25.62, 60.08) | 71.02 (52.9, 96.5) |  | 219.48 (130.46, 389.35) | 281.41 (177.05, 443.3) |
| Netherlands | 24322.59 (21814.34, 26398.39) | 31819.74 (29238.99, 34092.63) |  | 35181.94 (28243.67, 42689.65) | 37833.43 (30222.27, 46431.66) |
| New Zealand | 2752.75 (2483.97, 3018.63) | 2657.47 (2395.17, 2922.82) |  | 7182.91 (5560.63, 8892.26) | 5472.35 (4330.18, 6845.84) |
| Nicaragua | 12.96 (10.25, 16.37) | 16.3 (12.26, 20.99) |  | 53.47 (35.5, 76.61) | 53.79 (33.75, 82.44) |
| Niger | 1.13 (0.67, 2.02) | 3.17 (2.1, 5.25) |  | 3.29 (1.76, 5.71) | 5.93 (3.61, 10.05) |
| Nigeria | 51.55 (33.9, 74.48) | 86.61 (63.33, 118.25) |  | 142.94 (92.39, 209.63) | 156.54 (107.04, 224.19) |
| North Macedonia | 196.81 (150.75, 249.34) | 774.68 (575.72, 973.14) |  | 563.93 (348.49, 847.27) | 1658.73 (1018.21, 2500.3) |
| Norway | 7038.51 (6341.26, 7517.1) | 7731.28 (7179.48, 8190.15) |  | 9068.94 (7533.49, 10662.99) | 8342.56 (6944.68, 9674.69) |
| Oman | 37.27 (24.45, 51.92) | 143.78 (104.58, 207.24) |  | 40.46 (24.21, 61.46) | 156.75 (92.69, 244.2) |
| Pakistan | 379.11 (249.64, 539.72) | 1744.21 (1242, 2368.19) |  | 1525.43 (907.58, 2466) | 4236.17 (2548.93, 6659.7) |
| Panama | 181.19 (139.56, 220.69) | 302.3 (229.52, 372.69) |  | 1336.06 (894.96, 1853.83) | 1561.24 (1000.04, 2303.5) |
| Papua New Guinea | 19.93 (11.41, 33.09) | 49.61 (31.1, 78.45) |  | 89.16 (43.32, 154.29) | 144.59 (82.86, 239.14) |
| Paraguay | 109.02 (80.36, 149.22) | 334.47 (248.03, 443.95) |  | 373.07 (225.61, 565.01) | 852.48 (477.63, 1291.89) |
| Peru | 632.24 (450.27, 817.54) | 699.87 (496.97, 899.69) |  | 1479.02 (999.09, 2086.45) | 1257.94 (786.59, 1866.34) |
| Philippines | 1161.78 (907.26, 1455.92) | 2538.34 (1944.64, 3205.86) |  | 3248.11 (2363.53, 4392.02) | 4872.65 (3666.82, 6269.76) |
| Poland | 24227.95 (21633.37, 26744.49) | 47765.15 (42827.13, 52677.51) |  | 62649.53 (47197.41, 81368.91) | 119909.21 (92070.29, 153222.93) |
| Portugal | 2359.41 (2064.71, 2617.02) | 7590.51 (6939.57, 8247.41) |  | 4372.23 (3329.27, 5585.63) | 10014.33 (7835.58, 12473.81) |
| Qatar | 208.35 (144.16, 287.73) | 1116.8 (778.72, 1619.19) |  | 347.82 (222.68, 538.94) | 1472.57 (911.97, 2268.15) |
| Republic of Korea | 16177.1 (12818.66, 19529.31) | 44840.17 (38040.86, 52229.89) |  | 43748.7 (30765.91, 59490.26) | 78349.09 (59787.5, 101549.72) |
| Republic of Moldova | 148.96 (130.6, 170.89) | 665.87 (577.46, 768.35) |  | 730.57 (469.96, 1055.56) | 2476.13 (1496.19, 3642.29) |
| Romania | 7134.68 (6147.2, 8222.52) | 22451.13 (19562.64, 25606.47) |  | 24243.43 (16756.74, 32787.49) | 51895.48 (36260.98, 71768.33) |
| Russian Federation | 23558.66 (20957.78, 26012.89) | 97459.52 (86156.46, 107739.85) |  | 42880.31 (31603.42, 58360.72) | 115747.22 (84520.98, 155037.82) |
| Rwanda | 5.31 (2.7, 8.1) | 10.73 (5.55, 15.9) |  | 45.88 (22.34, 74.53) | 78.42 (37.62, 124.12) |
| Saint Lucia | 4.47 (3.64, 5.42) | 8.85 (7, 10.85) |  | 5.32 (3.38, 7.71) | 7.22 (4.29, 11.1) |
| Saint Vincent and the Grenadines | 2.85 (2.46, 3.32) | 5.04 (4.4, 5.78) |  | 6.15 (4, 8.66) | 6.93 (4.15, 10.61) |
| Samoa | 0.36 (0.22, 0.52) | 1.39 (1.03, 1.81) |  | 0.54 (0.3, 0.85) | 1.51 (1.02, 2.21) |
| Sao Tome and Principe | 0.3 (0.18, 0.48) | 0.87 (0.65, 1.16) |  | 1.32 (0.72, 2.19) | 3.42 (2.37, 4.85) |
| Saudi Arabia | 1340.14 (939.3, 1831.29) | 3906.6 (2979.01, 5115.37) |  | 2576.78 (1597.08, 4177.51) | 6531.8 (4004.18, 9822.48) |
| Senegal | 10.39 (7.77, 13.93) | 27.51 (19.76, 37.41) |  | 47.92 (33.75, 67.18) | 81.84 (56.46, 112.83) |
| Serbia | 2213.72 (1729.19, 2780.71) | 5653.46 (4371.67, 7067.04) |  | 5136.03 (3524.04, 7128.98) | 9134.53 (5952.5, 12868.22) |
| Seychelles | 6.24 (5.17, 7.57) | 20.31 (16.65, 24.73) |  | 18.42 (13.22, 25.13) | 40.44 (29.6, 51.79) |
| Sierra Leone | 1.32 (0.85, 1.81) | 3.64 (2.45, 5.16) |  | 7.93 (4.92, 12.16) | 13.24 (8.57, 20.13) |
| Singapore | 4158.37 (3728.94, 4614.32) | 7628.63 (6775.19, 8614.47) |  | 10672.67 (7951.77, 13496.68) | 13841.78 (10765.72, 17156.06) |
| Slovakia | 1135.79 (873.65, 1468.1) | 3814.4 (2916.81, 4756.29) |  | 1444.4 (1017.63, 1984.91) | 3669.76 (2449.94, 5015.96) |
| Slovenia | 1122.54 (915.71, 1325.12) | 2301.37 (1975.68, 2637.23) |  | 2365.97 (1754.21, 3169.52) | 3854.36 (2852.47, 5170.46) |
| Solomon Islands | 1.04 (0.73, 1.44) | 2.55 (1.86, 3.57) |  | 2.62 (1.68, 3.89) | 4.18 (2.79, 6) |
| Somalia | 1.69 (1.07, 2.8) | 5.86 (3.45, 12.06) |  | 13.99 (6.6, 25.86) | 39.93 (17.31, 90.64) |
| South Africa | 1811.94 (1577.39, 2068.75) | 4093.51 (3584.74, 4666.08) |  | 2372.67 (1848.29, 2972.26) | 3894.66 (3095.5, 4908.35) |
| Spain | 13785.19 (12157.44, 15329) | 45032.49 (41566.68, 48354.1) |  | 18186.9 (14228.74, 22921.62) | 44776.14 (36302.67, 55729.22) |
| Sri Lanka | 338.16 (224.45, 464.68) | 827.57 (512.07, 1160.73) |  | 777.08 (493.13, 1127.94) | 1346.19 (839.6, 1941.69) |
| Sudan | 33.3 (20.99, 52.36) | 85.05 (54.11, 131.84) |  | 72.29 (40.02, 130.15) | 106.46 (57.34, 177.5) |
| Suriname | 15.78 (11.9, 20.44) | 31.33 (23.13, 41.66) |  | 14.71 (9.33, 21.71) | 19.68 (11.36, 29.61) |
| Sweden | 7712.67 (6460.31, 8872.29) | 6640.19 (5609.9, 7744.41) |  | 12229.43 (9433, 15365.9) | 8407.54 (6652.67, 10427.16) |
| Switzerland | 8321.53 (7159.89, 9292) | 12659.44 (11381.49, 13921.9) |  | 10625.51 (8341.79, 12714.81) | 12237.3 (9985.54, 14668.35) |
| Tajikistan | 19.52 (11.12, 29.56) | 42.63 (27.76, 63.45) |  | 94.43 (53.08, 164.15) | 130.83 (71.09, 228.87) |
| Thailand | 6914.2 (5190.83, 8890.18) | 13291.23 (9978.33, 16928.72) |  | 11649.28 (7984.34, 16280.92) | 14777.5 (10414.01, 20276.89) |
| Timor-Leste | 4.63 (3.44, 6.16) | 10.7 (7.59, 14.74) |  | 2.62 (1.69, 3.85) | 4.74 (3.1, 6.89) |
| Togo | 2.64 (1.51, 3.87) | 7.42 (4.73, 10.63) |  | 11.08 (5.93, 17.2) | 21.6 (13.12, 33.01) |
| Tonga | 0.94 (0.68, 1.29) | 3.1 (2.38, 3.92) |  | 2.51 (1.63, 3.72) | 5.67 (3.92, 7.75) |
| Tunisia | 89.49 (61.7, 128.7) | 793.4 (520.58, 1127.81) |  | 144.06 (85.36, 234.44) | 760.72 (465.39, 1153.71) |
| Uganda | 17.96 (11.41, 25.95) | 19.75 (14.32, 27.2) |  | 62.27 (38.76, 92.76) | 46.06 (30.56, 65.82) |
| Ukraine | 1736.9 (1153.7, 2501.96) | 7881.54 (5116.94, 11355.3) |  | 3020.06 (1717.33, 4595.33) | 9087.82 (5364.25, 14268.43) |
| United Arab Emirates | 758.28 (500.48, 1115.01) | 2706.09 (1941.54, 4500.57) |  | 5284.43 (3019.71, 8536.14) | 9942.3 (6257.22, 16083.25) |
| United Kingdom | 48881.63 (45049.42, 51049.44) | 57202.67 (54293.26, 59141.11) |  | 79171.52 (64939.66, 96416.37) | 71274.5 (58579.9, 86580.19) |
| United Republic of Tanzania | 24.96 (17.19, 34.55) | 50.18 (33.67, 71.54) |  | 116.58 (75.27, 174.03) | 135.34 (81.71, 204.49) |
| United States of America | 406770.98 (370296.07, 428174.48) | 496512.32 (471816.05, 516558.78) |  | 614779.11 (491037.49, 739713.28) | 527855.88 (442149.11, 622705.1) |
| Uruguay | 451.24 (410.03, 494.63) | 1314.14 (1201, 1420.51) |  | 618.48 (474.04, 778.61) | 1477.06 (1165.2, 1845.08) |
| Uzbekistan | 160.29 (129.5, 194.82) | 382.34 (310.45, 461.04) |  | 509.23 (291.11, 794.32) | 917.07 (511.45, 1484.57) |
| Vanuatu | 0.44 (0.32, 0.63) | 1.59 (0.98, 2.7) |  | 1.67 (1.06, 2.52) | 3.56 (2.02, 6.63) |
| Viet Nam | 2560.92 (1881.23, 3353.96) | 6549.01 (4743.18, 8509.27) |  | 16623.84 (11401.12, 23023.89) | 29654.28 (20593.78, 40296.33) |
| Zambia | 17.5 (9.66, 30.2) | 36.57 (23.11, 75.81) |  | 41.13 (23.2, 67.67) | 59.75 (35.88, 118.48) |
| Zimbabwe | 15.04 (10.72, 21.26) | 20.53 (14.98, 27.68) |  | 23.36 (14.94, 34.76) | 28.43 (19.8, 41.76) |

Note: Data were available for 169 countries covering more than 99% of the world population. All future year estimates were discounted at a 3% annual discount rate.

**Supplementary Table 20** Estimated economic burden of tracheal, bronchus, and lung cancer by country through 2050 by sex, millions of 2023 US$. (E=1.0)

| Location | economic burden 2021 95%UI | |  | economic burden 2050 95%UI | |
| --- | --- | --- | --- | --- | --- |
|  | Female | Male |  | Female | Male |
| Afghanistan | 68.21 (37.85, 101.94) | 89.87 (51.65, 169.34) |  | 73.6 (32.98, 133.35) | 89.19 (36.2, 187.8) |
| Albania | 321.39 (225.38, 439.17) | 1023.27 (712.16, 1384.55) |  | 854.67 (499.85, 1305.88) | 1966.36 (1164.5, 2964.71) |
| Algeria | 387.67 (266.63, 525.33) | 1667.89 (1255.38, 2203.01) |  | 397.82 (239.67, 609.27) | 1264.3 (746.9, 1879.65) |
| Angola | 178.47 (117.3, 261.74) | 539.74 (380.14, 722.02) |  | 215.52 (134.55, 335.94) | 487.54 (331.92, 705.99) |
| Argentina | 7160.2 (6354.61, 7977.37) | 13886.39 (12531.95, 15277.98) |  | 12965.14 (9912.1, 16862.61) | 19877.99 (15432.3, 25479.71) |
| Armenia | 248.69 (215.71, 284.99) | 1240.29 (1092.06, 1410.46) |  | 736.58 (503.61, 1060.43) | 2605.48 (1725.18, 3814.21) |
| Australia | 15102.34 (13457.89, 16426.86) | 22173.5 (20208.78, 24337.92) |  | 29172.75 (22768.53, 36190.23) | 29140.31 (22877.98, 35408.55) |
| Austria | 8220.86 (7388.39, 8971.02) | 12028.52 (11064.06, 13214.58) |  | 10184.3 (8226.23, 12646.01) | 12288.91 (9755.11, 15091.47) |
| Azerbaijan | 519.7 (378.67, 700.72) | 2089.02 (1299.46, 2867.19) |  | 614.31 (388.11, 935.22) | 1697.55 (881.62, 2905.77) |
| Bahamas | 52.54 (42.18, 65.74) | 115.67 (91.84, 148.07) |  | 75.05 (50.19, 104.41) | 113.91 (70.65, 165.22) |
| Bahrain | 189.93 (146.08, 240.71) | 542.58 (408.12, 725.52) |  | 360.49 (231.78, 560.42) | 1301.14 (793.51, 1964.76) |
| Bangladesh | 1208.55 (776, 1831.82) | 3047.12 (2179.17, 4510.78) |  | 10191.58 (5912.28, 17073.39) | 15924.15 (9519.54, 25671) |
| Barbados | 20.85 (16.13, 26.43) | 36.59 (27.49, 46.61) |  | 13.01 (8.32, 18.59) | 13.76 (8.15, 20.63) |
| Belarus | 1063.99 (830.98, 1304.62) | 7390.05 (5695.4, 9113.96) |  | 1154.1 (746.08, 1642.59) | 5838.47 (3737.48, 8664.13) |
| Belgium | 10865.08 (9757.52, 11793.88) | 22832.97 (20647.5, 25092.5) |  | 16751.53 (13348.5, 20777.94) | 26823.99 (21192.44, 34003.4) |
| Belize | 8.35 (7.19, 9.62) | 18.06 (15.22, 21.09) |  | 19.61 (13.42, 27.62) | 27.78 (16.86, 41.77) |
| Benin | 23.41 (15.73, 33.8) | 61.54 (45.85, 82.42) |  | 163.89 (107.4, 247.63) | 259.39 (175.53, 356.43) |
| Bhutan | 10.97 (6.38, 17.01) | 20.71 (12.81, 30.58) |  | 68.55 (35.72, 117.41) | 107.86 (59.27, 185.3) |
| Bolivia (Plurinational State of) | 329.52 (190.28, 490.01) | 421.73 (296.07, 588.46) |  | 1739.23 (894.65, 2839.09) | 1835 (1118.27, 2961.22) |
| Bosnia and Herzegovina | 931.9 (727.75, 1183.38) | 2996.36 (2294.45, 3819.09) |  | 2737.31 (1758.28, 3981.41) | 6135.92 (3976.21, 9006.54) |
| Botswana | 102.41 (71.62, 140.51) | 245.97 (161.47, 330.45) |  | 378.93 (249.13, 551.78) | 656.95 (414.4, 913.5) |
| Brazil | 20446.18 (19007.03, 21685.43) | 26453.18 (24869.49, 27863.28) |  | 20604.42 (14140.34, 28562.55) | 21673.67 (13954.26, 32424.09) |
| Brunei Darussalam | 213.02 (169.06, 261.83) | 263.22 (206.6, 328.87) |  | 158.49 (111.22, 220.3) | 154.07 (108.62, 215.37) |
| Bulgaria | 3276.23 (2696.86, 3895.16) | 10157.77 (8483.46, 11878.02) |  | 7090.66 (5134.01, 9481.77) | 15539.37 (10952.49, 21558.46) |
| Burkina Faso | 22.92 (14.49, 34.27) | 69.27 (50.43, 104.8) |  | 150.31 (90.07, 240.6) | 283.13 (190.97, 414.38) |
| Burundi | 4.22 (2.64, 6.38) | 14.11 (9.79, 21.47) |  | 15.07 (9.01, 24.57) | 30.89 (19.45, 49.32) |
| Cabo Verde | 11.86 (8.84, 16) | 26.85 (19.87, 33.7) |  | 30.99 (21.02, 44.01) | 54.56 (36.75, 74.01) |
| Cambodia | 338.71 (233.64, 464.13) | 868.34 (609.62, 1177.6) |  | 1878.09 (1194.52, 2826.99) | 3875.06 (2448.65, 5590.58) |
| Cameroon | 88.09 (57.04, 127.16) | 237.73 (157.02, 337.43) |  | 388.05 (241.87, 592.43) | 678.92 (449.72, 971.48) |
| Canada | 38866.51 (35180.04, 42267.52) | 43319.88 (39830.01, 47054.1) |  | 58680.44 (47576.29, 72941.7) | 45202.06 (36608.5, 54681.04) |
| Central African Republic | 4.31 (3.04, 6.02) | 18.35 (10.37, 37.9) |  | 6.16 (3.69, 9.36) | 14.7 (6.89, 33.71) |
| Chad | 10.16 (6.4, 14.45) | 38.33 (26.53, 57.44) |  | 17.66 (10.52, 27.43) | 31.61 (19.75, 47.75) |
| Chile | 2741.87 (2461.9, 3030.04) | 3986.4 (3606.11, 4345.86) |  | 5920.89 (4675.7, 7315.73) | 6274.63 (5017.34, 7481.39) |
| China | 424485.55 (331530.79, 530352.7) | 894653.36 (652044.11, 1166488.12) |  | 1906726.35 (1312485.01, 2672770.59) | 2865539.33 (1965926.34, 3829529.21) |
| Colombia | 3018.59 (2536.06, 3555.41) | 3990.6 (3216.09, 4843.9) |  | 9894.82 (6801.41, 13497.42) | 10004.95 (6345.86, 14205.14) |
| Comoros | 3.54 (2.09, 5.22) | 6.62 (3.75, 9.86) |  | 10.09 (5.57, 15.55) | 14.98 (7.65, 23.16) |
| Congo | 37.61 (22.07, 58.48) | 83.41 (59.45, 114.82) |  | 17.07 (9.02, 27.62) | 31 (20.68, 45.76) |
| Costa Rica | 239.51 (208.69, 272) | 397.73 (344.15, 459.36) |  | 991.29 (693.8, 1385.62) | 1153.67 (773.1, 1635.78) |
| Croatia | 2697.75 (2243.62, 3228.27) | 6973.73 (5968.25, 8034.36) |  | 7301.69 (5249.84, 9953.99) | 15997.81 (11662.98, 20827.84) |
| Cyprus | 279.48 (211.4, 342.59) | 959.76 (767.24, 1161.73) |  | 604.05 (410.5, 838.33) | 1371.57 (979.16, 1889.98) |
| Czechia | 7810.12 (6745.67, 8902.01) | 15134.74 (13181.22, 17255.16) |  | 17964.22 (13158.29, 23714.43) | 29268.3 (21953.57, 38010.39) |
| Democratic Republic of the Congo | 75.14 (45.12, 114.4) | 203.3 (109.28, 400.39) |  | 813.9 (423.96, 1337.89) | 1639.76 (731.17, 3662.98) |
| Denmark | 10551.45 (9396.72, 11507.47) | 11052.77 (9989.18, 12237.48) |  | 21092.83 (16409.43, 25387.75) | 15836.1 (12614.77, 19309) |
| Djibouti | 7.27 (4.73, 10.43) | 25.12 (14.79, 38.34) |  | 62.04 (39.75, 95.09) | 127.76 (70.53, 200.27) |
| Dominican Republic | 769.4 (533.58, 1063.46) | 1391.26 (915.23, 1929.62) |  | 5370.29 (3129.02, 7958.74) | 7688.8 (4187.76, 12321.39) |
| Ecuador | 452.59 (356.79, 568.13) | 561.53 (423.56, 731.32) |  | 874.97 (598.13, 1241.69) | 797.43 (500.73, 1191.2) |
| Egypt | 2454.03 (1939.2, 3038.93) | 7219.7 (5618.04, 9190.11) |  | 7548.15 (4986.93, 10796.75) | 16399.95 (10543.85, 23754.49) |
| El Salvador | 196.55 (153.13, 253.2) | 204.19 (159.71, 262.51) |  | 738.07 (480.78, 1109.03) | 528.4 (320.79, 809.13) |
| Equatorial Guinea | 27.17 (14.98, 44.98) | 52.81 (34.32, 76.59) |  | 10.76 (6.01, 17.85) | 19.65 (12.23, 29.05) |
| Estonia | 543.03 (457.38, 627.29) | 1798.64 (1522.96, 2055.39) |  | 1484.73 (1034.57, 2074.33) | 3817.93 (2717.61, 5282.06) |
| Ethiopia | 121.46 (95.17, 154.21) | 484 (365.17, 621.27) |  | 2690.1 (1812.42, 3768.41) | 7713.04 (5254.58, 10816.96) |
| Fiji | 25.96 (19.1, 34.12) | 45.75 (33.77, 60.63) |  | 63.53 (40.12, 92.01) | 94.15 (62.96, 133.27) |
| Finland | 3742.07 (3306.41, 4131.18) | 6919.44 (6159.53, 7688.41) |  | 5257.88 (4043.22, 6452.24) | 7171.5 (5680.55, 9005.91) |
| France | 48262.66 (42431.72, 53622.94) | 115183.93 (104244.14, 125906.55) |  | 75107.08 (58970.48, 92772.27) | 140551.2 (114244.19, 172425.18) |
| Gabon | 46.81 (30.62, 69.12) | 136.93 (96.46, 189.92) |  | 68.98 (40.98, 104.43) | 124.36 (82.03, 175.93) |
| Gambia | 1.21 (0.7, 1.87) | 4.1 (2.92, 5.69) |  | 2.9 (1.65, 4.64) | 5.89 (3.74, 8.58) |
| Georgia | 324.29 (278.69, 377.57) | 2041.5 (1757.64, 2350.86) |  | 978.87 (700.8, 1368.73) | 7247.01 (5102.72, 10168.43) |
| Germany | 91943.61 (83646.4, 98960.99) | 155860.09 (143207.55, 169215.44) |  | 124420.56 (99171.09, 149848.81) | 164082.2 (135897.4, 195107.16) |
| Ghana | 99.55 (74.09, 132.36) | 339.28 (242.58, 437.58) |  | 315.42 (209.42, 461.85) | 847.37 (573.16, 1183.09) |
| Greece | 4657.6 (4206.92, 5049.46) | 15927.06 (14805.52, 17008.33) |  | 5027.32 (3868.27, 6389.7) | 12539.09 (9709.66, 15739.37) |
| Grenada | 5.19 (4.43, 5.98) | 11.1 (9.29, 13.02) |  | 14.07 (9.68, 19.82) | 19.88 (12.32, 30.64) |
| Guatemala | 223.72 (190.47, 261.44) | 228.25 (191.05, 271.99) |  | 659.03 (468.94, 903.3) | 478.27 (301.8, 682.01) |
| Guinea | 17.21 (9.88, 24.97) | 72.53 (50.32, 103.04) |  | 107.97 (63.75, 164.89) | 239.59 (156.91, 357.48) |
| Guinea-Bissau | 3.04 (2.13, 4.2) | 8 (5.6, 11.52) |  | 22.56 (13.87, 33.56) | 41.45 (26.26, 65.55) |
| Guyana | 44.39 (32.04, 59.79) | 67.6 (49.1, 88.92) |  | 518.86 (327.71, 756.73) | 532.63 (324.5, 815.35) |
| Haiti | 75.26 (48.93, 111.38) | 151.27 (84.3, 270.89) |  | 130.17 (69.96, 228.51) | 196.89 (91.99, 399.26) |
| Honduras | 278.97 (167.15, 460.42) | 251.39 (188.9, 342.77) |  | 1451.11 (747.41, 2613.41) | 920.5 (537, 1506.35) |
| Hungary | 12023.23 (10153.92, 13836.29) | 18217.73 (15694.91, 20898.66) |  | 24913.82 (17665.63, 33311.02) | 30237.49 (21134.27, 40449.91) |
| Iceland | 347.24 (305.95, 387.76) | 241.21 (213.45, 270.12) |  | 832.08 (648.73, 1027.04) | 558.89 (440.33, 685.74) |
| India | 14832.65 (12321.71, 17752.21) | 33354.32 (26548.66, 39921.26) |  | 63286.68 (41234.94, 90904.86) | 91029.07 (58436.6, 131537.88) |
| Indonesia | 184084.37 (104902.69, 264220.6) | 390688.81 (265852.94, 524820.63) |  | 537363.13 (285278.86, 802015.7) | 868937.76 (576994.73, 1191056.71) |
| Iran (Islamic Republic of) | 3328.46 (2797.53, 3806.89) | 7259.89 (6535.52, 8086.6) |  | 4824.69 (3137.82, 6984.97) | 7742.34 (5062.09, 10778.63) |
| Iraq | 745.54 (527.12, 1016.75) | 2008.94 (1413.32, 2570.53) |  | 408.51 (216.5, 648.58) | 837.66 (449.92, 1347.72) |
| Ireland | 5068.55 (4339.95, 5817.2) | 6051.24 (5473.66, 6634.16) |  | 41785.75 (32880.31, 52227.15) | 35442.65 (29324.53, 42564.99) |
| Israel | 1876.18 (1655.48, 2065.65) | 3929.44 (3550.42, 4316.88) |  | 3959.07 (2903.06, 5121.77) | 6143.59 (4715.81, 7923.87) |
| Italy | 42480.76 (37206.31, 46052.37) | 95497.16 (88890.79, 100517.2) |  | 54321.67 (42464.37, 69199.21) | 99715.54 (80861.44, 120707.98) |
| Jamaica | 99.39 (75.16, 127.09) | 281.21 (209.1, 376.24) |  | 192.06 (120.86, 279.26) | 379.23 (233.22, 576.6) |
| Japan | 72688.92 (58614.47, 80601.5) | 189293.64 (174955.99, 197562.09) |  | 66116.08 (47968.33, 85256.79) | 116886.54 (94163.08, 141787.27) |
| Jordan | 122.82 (82.33, 170.84) | 463.09 (347.98, 620.06) |  | 219.69 (128.98, 346.5) | 659.48 (414.98, 975.4) |
| Kazakhstan | 1207.71 (996.09, 1430.71) | 5158.48 (4375.07, 6025.55) |  | 2537.58 (1624.38, 3641.1) | 7277.96 (4646.51, 10613.36) |
| Kenya | 127 (84.74, 199.58) | 191.41 (146.43, 241.59) |  | 1851.93 (1183.03, 2902.47) | 1834.82 (1327.16, 2446.58) |
| Kiribati | 0.99 (0.64, 1.41) | 3.75 (2.73, 5.08) |  | 3.73 (2.18, 5.78) | 10.26 (6.72, 14.61) |
| Kuwait | 235.81 (200.11, 280.2) | 888.09 (703.23, 1114.43) |  | 215.77 (145.02, 297.54) | 702.85 (442.99, 1022.22) |
| Lao People's Democratic Republic | 188.13 (122.17, 308.84) | 462.45 (320.71, 642.43) |  | 1732.16 (1006.99, 3012.44) | 3246.5 (2202.48, 4710.4) |
| Latvia | 661.09 (551.63, 768.38) | 2350.11 (1962.49, 2678.52) |  | 1444.33 (1039.53, 1965.61) | 3923.21 (2787.53, 5268.08) |
| Lebanon | 379.2 (256.01, 512.97) | 883.45 (661.53, 1136.14) |  | 275.77 (166.03, 430.4) | 523.31 (331.81, 810.44) |
| Lesotho | 19.6 (10.96, 30.8) | 71.32 (47.54, 106) |  | 25.37 (13.28, 43.62) | 88.08 (57.73, 128.69) |
| Liberia | 3.66 (2.17, 5.32) | 9.06 (5.38, 15.56) |  | 21.41 (12.17, 33.44) | 36.66 (21.33, 59.41) |
| Libya | 282.2 (178.65, 414.34) | 1995.3 (1386.59, 2791.51) |  | 282.42 (163.65, 461.69) | 1590.73 (936.77, 2480.77) |
| Lithuania | 1062.45 (894.53, 1252.71) | 4356.58 (3696.71, 5007.21) |  | 3734.66 (2626.08, 5051.24) | 10334.74 (7380.04, 13970.26) |
| Luxembourg | 595.56 (530.03, 665.72) | 1235.31 (1093.36, 1378.89) |  | 985.27 (770.88, 1190.42) | 1630.71 (1298.47, 1970.94) |
| Madagascar | 28.9 (19.41, 40.63) | 54.44 (38.29, 73.89) |  | 64.31 (42.34, 94.34) | 82 (55.37, 117.6) |
| Malawi | 4.61 (2.59, 6.9) | 20.95 (14.63, 29.52) |  | 10.54 (5.89, 16.81) | 34.89 (22.58, 51.75) |
| Malaysia | 3271.67 (2726.71, 3921.82) | 8101.62 (6694.13, 9392.35) |  | 10000.97 (7114.07, 14176.94) | 16873.28 (12132.26, 22983.07) |
| Maldives | 8.56 (6.57, 10.73) | 24.12 (17.72, 30.42) |  | 89.85 (62.16, 126.94) | 271.21 (185.82, 370.32) |
| Mali | 23.65 (16.32, 33.11) | 42.37 (28.58, 58.26) |  | 122.88 (80.67, 186.44) | 140.36 (86.27, 209.8) |
| Malta | 156.01 (133.53, 181.59) | 435.91 (389.81, 483.53) |  | 455.57 (336.5, 583.22) | 1015.42 (759.15, 1297.98) |
| Mauritania | 20.15 (14.06, 27.91) | 37.91 (25.33, 57.08) |  | 78.29 (53.53, 114.21) | 95.99 (60.15, 141.49) |
| Mauritius | 126.56 (115.16, 138.07) | 309.81 (281.63, 335.98) |  | 279.31 (209.75, 361.78) | 485.24 (368.45, 619.65) |
| Mexico | 5236.63 (4435.83, 6053.98) | 7959.86 (6677.25, 9415.72) |  | 8244.6 (5992.21, 10899.38) | 8560.87 (5834.02, 12045.92) |
| Micronesia (Federated States of) | 2.47 (1.71, 3.39) | 6.39 (4.15, 9.57) |  | 4.08 (2.49, 6.15) | 7.62 (4.6, 11.99) |
| Mongolia | 119.24 (89.17, 152.32) | 447.27 (345.21, 579.7) |  | 251.25 (158.99, 371.24) | 705.7 (418, 1059.45) |
| Montenegro | 270.74 (210.34, 335.92) | 786.29 (594.34, 993.34) |  | 872.74 (594, 1268.38) | 1807.96 (1201.55, 2567.59) |
| Morocco | 278.47 (190.25, 388.27) | 3114.61 (2254.89, 3954.7) |  | 654.61 (387.74, 1044) | 4790.98 (2884.73, 7336.02) |
| Mozambique | 22.78 (14.17, 32.18) | 53.31 (40.21, 69.28) |  | 72.82 (44.67, 111.15) | 108.08 (75.71, 149.16) |
| Myanmar | 1233.07 (907.27, 1719.9) | 2191.09 (1535.13, 2976.42) |  | 1971.62 (1255.38, 2857.04) | 2763.21 (1860.36, 4054.2) |
| Namibia | 26.83 (17.38, 38.41) | 48.58 (35.9, 63.69) |  | 39.64 (26.01, 60.46) | 54.56 (39.16, 74.72) |
| Nepal | 156.63 (104.44, 244.91) | 289.51 (215.67, 393.39) |  | 894.73 (531.83, 1587.25) | 1147.2 (721.77, 1807.19) |
| Netherlands | 25342.49 (22729.07, 27505.33) | 33154.01 (30465.04, 35522.21) |  | 36657.19 (29427.99, 44479.72) | 39419.87 (31489.55, 48378.64) |
| New Zealand | 3381.42 (3051.25, 3708.02) | 3264.37 (2942.17, 3590.33) |  | 8823.31 (6830.55, 10923.05) | 6722.11 (5319.09, 8409.27) |
| Nicaragua | 45.08 (35.65, 56.92) | 56.68 (42.64, 73.01) |  | 185.96 (123.46, 266.44) | 187.09 (117.37, 286.74) |
| Niger | 8.23 (4.87, 14.81) | 23.15 (15.36, 38.36) |  | 24.04 (12.86, 41.74) | 43.35 (26.41, 73.47) |
| Nigeria | 188.31 (123.82, 272.1) | 316.4 (231.36, 431.97) |  | 522.19 (337.53, 765.82) | 571.87 (391.02, 818.98) |
| North Macedonia | 390.15 (298.85, 494.28) | 1535.71 (1141.3, 1929.14) |  | 1117.93 (690.83, 1679.61) | 3288.23 (2018.48, 4956.55) |
| Norway | 6486.57 (5843.99, 6927.62) | 7125.01 (6616.48, 7547.9) |  | 8357.77 (6942.73, 9826.82) | 7688.35 (6400.1, 8916.02) |
| Oman | 52.92 (34.72, 73.72) | 204.16 (148.49, 294.26) |  | 57.46 (34.38, 87.27) | 222.57 (131.62, 346.75) |
| Pakistan | 1339.22 (881.88, 1906.58) | 6161.51 (4387.42, 8365.76) |  | 5388.68 (3206.07, 8711.29) | 14964.52 (9004.25, 23525.76) |
| Panama | 274.45 (211.4, 334.29) | 457.91 (347.67, 564.55) |  | 2023.82 (1355.65, 2808.12) | 2364.91 (1514.83, 3489.27) |
| Papua New Guinea | 85.77 (49.12, 142.41) | 213.53 (133.87, 337.64) |  | 383.72 (186.42, 664.04) | 622.31 (356.64, 1029.24) |
| Paraguay | 240.8 (177.49, 329.6) | 738.76 (547.85, 980.59) |  | 824.01 (498.32, 1247.96) | 1882.91 (1054.97, 2853.46) |
| Peru | 1493.31 (1063.51, 1931) | 1653.06 (1173.83, 2125.03) |  | 3493.39 (2359.8, 4928.11) | 2971.21 (1857.89, 4408.22) |
| Philippines | 3221.67 (2515.87, 4037.32) | 7038.9 (5392.55, 8889.97) |  | 9007.12 (6554.16, 12179.22) | 13512.03 (10168.22, 17386.28) |
| Poland | 33817.16 (30195.67, 37329.73) | 66670.19 (59777.73, 73526.81) |  | 87445.66 (65877.74, 113574.02) | 167368.24 (128510.92, 213867.24) |
| Portugal | 3300.03 (2887.84, 3660.35) | 10616.6 (9706.15, 11535.38) |  | 6115.29 (4656.54, 7812.44) | 14006.72 (10959.37, 17446.71) |
| Qatar | 174.91 (121.02, 241.55) | 937.54 (653.73, 1359.3) |  | 291.99 (186.94, 452.43) | 1236.21 (765.59, 1904.09) |
| Republic of Korea | 19775.32 (15669.87, 23873.16) | 54813.84 (46502.17, 63847.22) |  | 53479.6 (37609.08, 72722.5) | 95776.04 (73085.86, 124137.11) |
| Republic of Moldova | 319.94 (280.5, 367.04) | 1430.13 (1240.25, 1650.23) |  | 1569.08 (1009.35, 2267.07) | 5318.11 (3213.45, 7822.73) |
| Romania | 10113.44 (8713.7, 11655.46) | 31824.61 (27730.15, 36297.32) |  | 34365.2 (23752.77, 46476.45) | 73562.13 (51400.14, 101732.01) |
| Russian Federation | 34545.72 (30731.87, 38144.53) | 142911.74 (126337.28, 157986.52) |  | 62878.41 (46342.32, 85578.43) | 169728.28 (123939.05, 227342.85) |
| Rwanda | 28.79 (14.63, 43.92) | 58.16 (30.07, 86.18) |  | 248.76 (121.14, 404.05) | 425.15 (203.94, 672.89) |
| Saint Lucia | 10.09 (8.21, 12.24) | 19.98 (15.81, 24.5) |  | 12.01 (7.64, 17.41) | 16.29 (9.68, 25.06) |
| Saint Vincent and the Grenadines | 6.21 (5.36, 7.24) | 11 (9.59, 12.6) |  | 13.41 (8.73, 18.89) | 15.11 (9.06, 23.13) |
| Samoa | 1.23 (0.76, 1.78) | 4.79 (3.54, 6.22) |  | 1.84 (1.04, 2.94) | 5.19 (3.53, 7.59) |
| Sao Tome and Principe | 1.29 (0.76, 2.04) | 3.73 (2.78, 4.97) |  | 5.67 (3.08, 9.39) | 14.66 (10.17, 20.78) |
| Saudi Arabia | 1559.9 (1093.33, 2131.59) | 4547.21 (3467.51, 5954.2) |  | 2999.33 (1858.98, 4862.55) | 7602.91 (4660.8, 11433.21) |
| Senegal | 45.15 (33.76, 60.5) | 119.49 (85.83, 162.51) |  | 208.14 (146.61, 291.8) | 355.5 (245.25, 490.1) |
| Serbia | 4108.55 (3209.29, 5160.86) | 10492.53 (8113.61, 13116.08) |  | 9532.21 (6540.44, 13231.03) | 16953.24 (11047.54, 23882.79) |
| Seychelles | 9.8 (8.12, 11.89) | 31.9 (26.14, 38.83) |  | 28.93 (20.76, 39.47) | 63.51 (46.49, 81.33) |
| Sierra Leone | 8.41 (5.43, 11.51) | 23.13 (15.59, 32.79) |  | 50.4 (31.25, 77.28) | 84.15 (54.46, 127.95) |
| Singapore | 3517.33 (3154.1, 3902.99) | 6452.62 (5730.74, 7286.48) |  | 9027.4 (6725.94, 11416.07) | 11707.97 (9106.11, 14511.33) |
| Slovakia | 1654.27 (1272.47, 2138.28) | 5555.66 (4248.32, 6927.52) |  | 2103.77 (1482.18, 2891.02) | 5344.99 (3568.33, 7305.74) |
| Slovenia | 1435.44 (1170.96, 1694.48) | 2942.86 (2526.38, 3372.33) |  | 3025.46 (2243.17, 4052.99) | 4928.72 (3647.56, 6611.67) |
| Solomon Islands | 5.38 (3.77, 7.42) | 13.13 (9.6, 18.38) |  | 13.49 (8.66, 20.03) | 21.52 (14.39, 30.93) |
| Somalia | 11.28 (7.15, 18.66) | 39.04 (23, 80.43) |  | 93.3 (44.01, 172.42) | 266.24 (115.41, 604.37) |
| South Africa | 4021.49 (3500.91, 4591.46) | 9085.29 (7956.1, 10356.07) |  | 5265.99 (4102.17, 6596.75) | 8643.95 (6870.26, 10893.78) |
| Spain | 18047.42 (15916.39, 20068.55) | 58956.04 (54418.64, 63304.66) |  | 23810.09 (18628.11, 30008.74) | 58620.44 (47527.06, 72960.09) |
| Sri Lanka | 754.46 (500.75, 1036.72) | 1846.35 (1142.46, 2589.65) |  | 1733.7 (1100.2, 2516.5) | 3003.42 (1873.2, 4332.01) |
| Sudan | 141.85 (89.39, 223.03) | 362.29 (230.51, 561.62) |  | 307.92 (170.47, 554.42) | 453.49 (244.27, 756.09) |
| Suriname | 35.23 (26.58, 45.64) | 69.97 (51.66, 93.04) |  | 32.85 (20.85, 48.48) | 43.96 (25.37, 66.13) |
| Sweden | 8209.57 (6876.53, 9443.9) | 7068 (5971.32, 8243.35) |  | 13017.33 (10040.73, 16355.87) | 8949.21 (7081.28, 11098.95) |
| Switzerland | 8052.62 (6928.52, 8991.73) | 12250.34 (11013.69, 13472.01) |  | 10282.15 (8072.22, 12303.92) | 11841.85 (9662.85, 14194.34) |
| Tajikistan | 72.25 (41.15, 109.42) | 157.81 (102.77, 234.89) |  | 349.57 (196.5, 607.7) | 484.33 (263.18, 847.29) |
| Thailand | 13676.38 (10267.53, 17584.9) | 26290.24 (19737.28, 33485.26) |  | 23042.44 (15793.14, 32203.89) | 29230.1 (20599.07, 40107.99) |
| Timor-Leste | 15.85 (11.8, 21.09) | 36.67 (25.99, 50.49) |  | 8.98 (5.8, 13.18) | 16.24 (10.62, 23.6) |
| Togo | 14.54 (8.31, 21.28) | 40.8 (26.02, 58.46) |  | 60.97 (32.63, 94.65) | 118.83 (72.2, 181.58) |
| Tonga | 2.97 (2.15, 4.05) | 9.78 (7.49, 12.36) |  | 7.91 (5.12, 11.72) | 17.87 (12.35, 24.42) |
| Tunisia | 225.82 (155.71, 324.77) | 2002.14 (1313.67, 2846.01) |  | 363.52 (215.4, 591.61) | 1919.66 (1174.39, 2911.36) |
| Uganda | 97.35 (61.85, 140.68) | 107.06 (77.65, 147.44) |  | 337.61 (210.12, 502.89) | 249.71 (165.71, 356.82) |
| Ukraine | 3933.35 (2612.64, 5665.89) | 17848.4 (11587.74, 25715.01) |  | 6839.19 (3889.04, 10406.52) | 20580.12 (12147.79, 32312.04) |
| United Arab Emirates | 728.14 (480.59, 1070.7) | 2598.54 (1864.37, 4321.69) |  | 5074.4 (2899.69, 8196.88) | 9547.15 (6008.53, 15444.02) |
| United Kingdom | 57932.9 (53391.09, 60502.12) | 67794.72 (64346.58, 70092.1) |  | 93831.47 (76964.34, 114269.51) | 84472.19 (69426.97, 102611.99) |
| United Republic of Tanzania | 125.81 (86.64, 174.12) | 252.93 (169.69, 360.56) |  | 587.58 (379.39, 877.16) | 682.15 (411.82, 1030.67) |
| United States of America | 406770.98 (370296.07, 428174.48) | 496512.32 (471816.05, 516558.78) |  | 614779.11 (491037.49, 739713.28) | 527855.88 (442149.11, 622705.1) |
| Uruguay | 791.15 (718.89, 867.21) | 2304.03 (2105.66, 2490.52) |  | 1084.35 (831.12, 1365.1) | 2589.67 (2042.9, 3234.88) |
| Uzbekistan | 459.65 (371.37, 558.66) | 1096.4 (890.24, 1322.07) |  | 1460.28 (834.77, 2277.8) | 2629.78 (1466.63, 4257.15) |
| Vanuatu | 1.98 (1.45, 2.84) | 7.13 (4.39, 12.11) |  | 7.49 (4.76, 11.3) | 15.98 (9.06, 29.78) |
| Viet Nam | 6474.04 (4755.77, 8478.84) | 16555.96 (11990.81, 21511.51) |  | 42025.23 (28822.14, 58204.64) | 74966.31 (52061.29, 101869.52) |
| Zambia | 77.45 (42.74, 133.7) | 161.88 (102.29, 335.56) |  | 182.07 (102.71, 299.54) | 264.51 (158.81, 524.47) |
| Zimbabwe | 84 (59.86, 118.77) | 114.66 (83.7, 154.59) |  | 130.48 (83.44, 194.17) | 158.81 (110.61, 233.24) |

Note: Data were available for 169 countries covering more than 99% of the world population. All future year estimates were discounted at a 3% annual discount rate.

**Supplementary section 1:** The methodology of decomposed analysis.

In the GBD study, Tracheal, bronchus, and lung cancer (TBL) cancer is categorized as an "age-related disease." This classification stems from the observation that the incidence rate of TBL cancer among adults aged over 25 years increases exponentially with age (1). To analyze the changes in age-related TBL cancer deaths and DALY, we adopted the decomposition method developed by Gupta (2). Specifically, all population data used in the decomposition—including total adult population size and age structure—were obtained directly from the GBD population estimates, which provide age- and sex-specific population counts for each location and year. Population aging was therefore operationalized through changes in the age distribution of the adult population rather than through aggregate aging indices. This approach allows for the disentangling of the overall change in TBL cancer deaths and DALYs into contributions from four factors:

1. Adult Population Size: This term represents the total number of individuals within the adult population, aged 25 years and older. Changes in the size of the adult population can significantly influence the number of TBL cancer cases and associated outcomes.
2. Age Structure of the Adult Population: The distribution of the adult population across different age cohorts is critical, as the risk of TBL cancer escalates with age. Variations in the age structure of the population can, therefore, alter the overall burden of TBL cancer.
3. Disease Incidence or Prevalence: This factor relates to the rate at which new cases occur (incidence) or the total number of existing cases (prevalence) of TBL cancer within the adult population over a specific period.
4. Disease Fatality and Severity: The Fatality and severity of TBL cancer, reflected by mortality rates and the degree of disability caused, are pivotal in determining the health burden.

The decomposition approach employs a formula that quantifies the contributions of these four terms to the overall changes in age-related TBL cancer deaths and DALY (3).

$$D\text{ea}th_{y}=\sum_{a} popsize_{y}(A)\cdot\frac{popage_{a,y}}{popsize_{y}}(B)\cdot\frac{case_{a,y}}{popage_{a,y}}(C)\cdot\frac{death_{a,y}}{case_{a,y}}(D)$$

$$DALY_{y}=\sum_{a} popsize_{y}(A)\cdot\frac{popage_{a,y}}{popsize_{y}}(B)\cdot\frac{\text{prevalence}_{a,y}}{popage_{a,y}}(C)\cdot\frac{DALY_{a,y}}{\text{prevalence}_{a,y}}(D)$$

where a is the 5-year age groups among population aged ≥ 25 years (up to the ≥ 90-year-old age group), and y is the calendar year. The terms of A, B, C, and D represent the size of adult population, age structure of adult population, TBL cancer incidence (for death) or prevalence (for DALY), and disease fatality (for death) and severity (for DALY), respectively.

Note: Decomposition components may occasionally exceed +100% or fall below −100%. This occurs when the numerical change attributed to an individual term moves in the opposite direction of the overall trend or is larger in magnitude than the net observed change. Such values reflect the mathematical partitioning of observed changes—rather than epidemiologic effect sizes or causal relationships—and indicate that one component more than offsets the total net change.

**Supplementary section 2:** The methodology of economic burden analysis.

We projected the global, regional, and national economic impact of TBL cancer up to the year 2050, employing the willingness-to-pay methodology. The value of statistical life (VSL) providing a method to quantify the balance between financial income and the mortality risk, which widely used in health economics research (4, 5). This approach enables the estimation of economic burdens by assessing how much societies are willing to invest to mitigate the risks associated with death, thereby offering a valuable metric for understanding the comprehensive economic implications of TBL cancer across different geographical and temporal scales. The VSL concept, when extended to encompass both mortality and morbidity, can be understood as the monetized equivalent of the full spectrum of measurable and intangible benefits associated with a healthy life (6). To generate worldwide estimates of the VSL and project the economic burden of TBL cancer, we utilized a methodology established in 2019 by an international consortium of benefit-cost analysis experts (7, 8). This model initiates with the baseline VSL Figure for the United States, valued at $11.2 million (2021 US dollars, adjusted for inflation from the 2015 estimate of $9.4 million by the U.S. Department of Health and Human Services (8, 9), applying a 3% discount rate). To calculate the VSL for all non-U.S. countries, we adopted the following formula:

${VSL}_{x}$=${VSL}_{USA}$× ($\frac{{GNIPC}_{x}}{{GNIPC}_{USA}}$)^E^

In this formula, ${VSL}_{x}$ represents the VSL for a given country, ${VSL}_{USA}$ is the base VSL from the United States of America, GNIPC is the 2021 Gross National Income (GNI) per capita, adjusted for Purchasing Power Parity (PPP) for the country or the United States of America, respectively. GNIPC data were obtained from the World Bank (10).E is the income elasticity of the VSL, reflecting how sensitive the VSL is to changes in income. We used an elasticity value of 1 for high-income countries and 1.5 for low- and middle-income countries following a previous meta-analysis study (8). World Bank 2023 definitions of country income group classifications were used throughout our analysis, including for future years (10).

In this study, we leveraged the Value of a Statistical Life Year (VSLY) to quantify the economic impact due to TBL cancer. The VSLY methodology involves apportioning the overall VSL across the expected remaining lifespan of individuals within a specific demographic, thereby estimating the monetary value of mortality risk reduction attributable to various policies or interventions (8). Data on median age and corresponding life expectancy were sourced from the World Population Prospects and World Health Organization life Tables (11, 12), facilitating the allocation of VSL uniformly across an individual’s remaining years, without applying discount rates. This methodology reflects practices in health economics where each DALY is equated to a VSLY loss. Such practices are prevalent in assessments across various health economic evaluations, including neonatal sepsis, immunization initiatives for children, enhancements in air quality, and surgery (13-17). Consequently, the cumulative economic impact of TBL cancer was calculated by summing the VSLYs lost across the population.

We forecasted the future VSLY for a given country by scaling the VSLY calculated for 2021 in accordance with anticipated income growth. This calculation involved determining the average annual growth rate of GNI per capita over the period 2012 to 2021 from the World Bank (10). It was hypothesized that the VSLY would increase annually at the same rate, culminating in the estimation of distinct VSLY Figures for the specified country for the years 2030, 2040, and 2050. Future estimates of the TBL cancer burden are derived from the GBD 2021 forecasting. Using mixed-effects models, the GBD 2021 provide cause-specific forecasts of DALYs by age and sex for 204 countries and territories, as well as globally, from 2022 to 2050. ^(18)^ Finally, the future economic burden associated with TBL cancer was estimated by multiplying the projected VSLY values for various countries by the corresponding DALY estimates. The 95% uncertainty intervals for economic burden estimates were generated using the uncertainty intervals of GBD DALYs. To account for uncertainty in income–VSL relationships, we evaluated two additional scenarios in which income elasticities of E = 1 and E = 1.5 were assumed for all countries.

**Reference**

1. Global burden of 87 risk factors in 204 countries and territories, 1990-2019: a systematic analysis for the Global Burden of Disease Study 2019. Lancet. 2020;396(10258):1223-49.

2. Chang AY, Skirbekk VF, Tyrovolas S, Kassebaum NJ, Dieleman JL. Measuring population ageing: an analysis of the Global Burden of Disease Study 2017. Lancet Public Health. 2019;4(3):e159-e67.

3. Wang C, Chang Y, Ren J, Wu Z, Zheng Y, Luo Z, et al. Modifiable risk-attributable and age-related burden of lung cancer in China, 1990-2019. Cancer. 2023;129(18):2871-86.

4. Viscusi WK, Aldy JE, National Bureau of Economic R. The value of a statistical life : a critical review of market estimates throughout the world. Cambridge, Mass: National Bureau of Economic Research; 2003.

5. Masterman CJ, Viscusi WK. Income Elasticities and Global Values of a Statistical Life. Journal of Benefit-Cost Analysis. 2017;8(2):226-50.

6. Nandi A, Counts N, Chen S, Seligman B, Tortorice D, Vigo D, et al. Global and regional projections of the economic burden of Alzheimer's disease and related dementias from 2019 to 2050: A value of statistical life approach. EClinicalMedicine. 2022;51:101580.

7. Robinson LA, Hammitt JK, Jamison DT, Walker DG. Conducting Benefit-Cost Analysis in Low- and Middle-Income Countries: Introduction to the Special Issue. J Benefit Cost Anal. 2019;10(Suppl 1):1-14.

8. Hammitt JK, O’Keeffe L, Robinson LA. Valuing Mortality Risk Reductions in Global Benefit-Cost Analysis. Journal of Benefit-Cost Analysis. 2019;10(S1):15-50.

9. Robinson LAaH, James K. and Cecchini, Michele and Chalkidou, Kalipso and Claxton, Karl and Cropper, Maureen L. and Eozenou, Patrick and de Ferranti, David and Deolalikar, Anil B. and Campos Guanais de Aguiar, Frederico and Jamison, Dean T. and Kwon, Soonman and Lauer, Jeremy Addison and O'Keeffe, Lucy and Walker, Damian and Whittington, Dale and Wilkinson, Thomas and Wilson, David and Wong, Brad. Reference Case Guidelines for Benefit-Cost Analysis in Global Health and Development. 2019.

10. World Bank. DataBank| The World Bank. 2023. [Available from: https://databank.worldbank.org/home.aspx.

11. WHO. World Health Organization Life Tables. 2020.

12. United Nations, Department of Economic and Social Affairs, Population Division. World population prospects. 2022

13. Sylvia LR, Benjamin CW, Steven JS. Economic burden of neonatal sepsis in sub-Saharan Africa. BMJ Global Health. 2018;3(1):e000347.

14. Watts E, Sim SY, Constenla D, Sriudomporn S, Brenzel L, Patenaude B. Economic Benefits of Immunization for 10 Pathogens in 94 Low- and Middle-Income Countries From 2011 to 2030 Using Cost-of-Illness and Value-of-Statistical-Life Approaches. VALUE IN HEALTH. 2021;24(1):78-85.

15. Yin H, Brauer M, Zhang J, Cai W, Navrud S, Burnett R, et al. Population ageing and deaths attributable to ambient PM2·5 pollution: a global analysis of economic cost. The Lancet Planetary Health. 2021;5(6):e356-e67.

16. Daniel Scott C, Blake CA, Dan P, John GM, Mark GS. Economic valuation of the impact of a large surgical charity using the value of lost welfare approach. BMJ Global Health. 2016;1(4):e000059.

17. Ranganathan K, Singh P, Raghavendran K, Wilkins EG, Hamill JB, Aliu O, et al. The Global Macroeconomic Burden of Breast Cancer: Implications for Oncologic Surgery. Ann Surg. 2021;274(6):1067-72.

18. Burden of disease scenarios for 204 countries and territories, 2022-2050: a forecasting analysis for the Global Burden of Disease Study 2021. Lancet. 2024;403(10440):2204-56.
